# Elaboration of newly synthesized tetrahydrobenzo[*b*]thiophene derivatives and exploring their antioxidant evaluation, molecular docking, and DFT studies

**DOI:** 10.1038/s41598-024-74275-x

**Published:** 2024-11-09

**Authors:** Mina G. Balamon, Ashraf A. Hamed, Eman A. El-Bordany, Ahmed E. Swilem, Naglaa F. H. Mahmoud

**Affiliations:** Department of Chemistry, Faculty of Science, Ain Shams University, Cairo, 11566 Egypt

**Keywords:** Antioxidant, Docking, DFT, 2-amino thiophene-3-carbonitrile, Molecular medicine, Chemistry

## Abstract

Herein, 2-amino-6-(tert-butyl)-4,5,6,7-tetrahydrobenzo[*b*]thiophene-3-carbonitrile **(1)** was synthesized in excellent yield through gewald reaction in multi components one pot reaction. Compound **1** was utilized as a building block to synthesize a new group of tetrahydro benzo[*b*] thiophene congeners. The chemical structure of all the novel tetrahydro benzo[*b*]thiophene derivatives were elucidated through the melting point, elemental analysis, FT-IR, ^1^H-NMR, and mass spectroscopy. Furthermore, the total antioxidant capacity (TAC) of all the newly synthesized heterocyclic derivatives was evaluated according to the phosphomolybdenum method using ascorbic acid as standard. The findings revealed that compounds **1**, **16**, and **17** demonstrated significant antioxidant potency comparable to that of ascorbic acid. This suggests the potential of these heterocycles as promising candidates for antioxidant drugs in the treatment of oxidative stress-related diseases. Finally, molecular docking was conducted to study the binding affinity for the most potent antioxidant compounds **1**, **16, 17** and ascorbic acid inside the interactions of compounds **1**, **16**, and **17** with the Keap1 (Kelch-like ECH-associated protein 1) protein (PDB: 7C5E), compared to the co-crystallized ligand triethylene glycol (PGE) and ascorbic acid as a reference drug for antioxidants. DFT calculations and global descriptors were calculated for the most potent compounds to correlate the relation between chemical structure and reactivity.

## Introduction

**Fig. 1:**
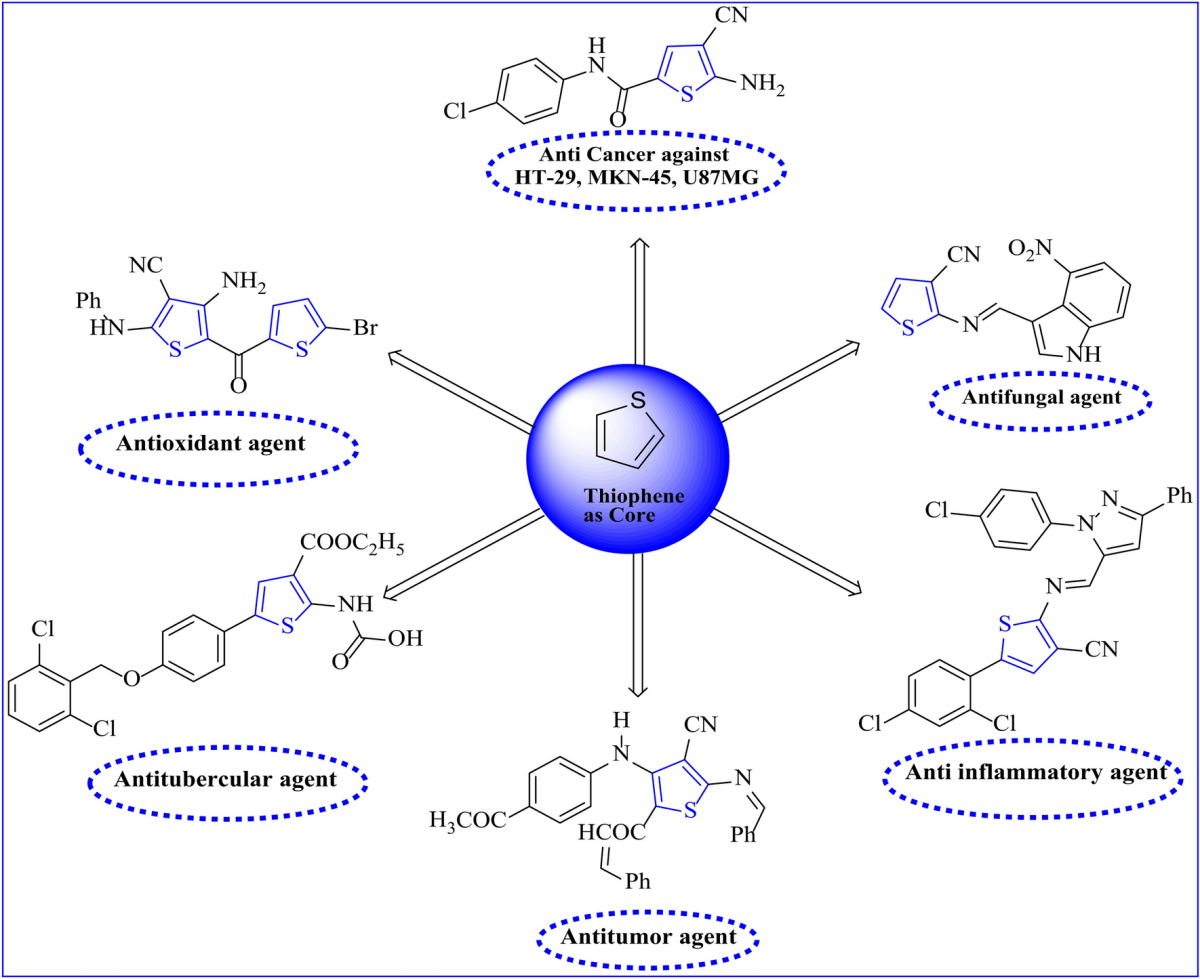
Various biological activities based on thiophene ring.

From medicinal chemistry to material science, S-heterocyclic cores, particularly those based on thiophene, are recognized for their paramount significance in diverse domains, including pharmaceuticals^1^, dyes^2^, and agrochemicals^3^. There are a wide range of biologically active products^4^, many of which demonstrate antifungal activity^5^, anticancer activity toward the six cancer cell lines (A549, H460, HT-29, MKN-45, U87MG, and SMMC)^6^, anti-inflammatory^7^, antioxidant^8^, antitumor^9^, antitubercular^10^ as demonstrated in **(**Fig. 1**)**, anticoagulant and antithrombotic activities^11^. The 2-Amino thiophene derivatives stand out as crucial intermediates in organic synthesis, giving rise to diverse heterocyclic systems with useful applications^12,13^. For The synthesis of thiophen-2-amines, involving the challenging introduction of an amino group into an existing thiophene moiety, has garnered attention in organic chemistry. Numerous methodologies for synthesizing 2-aminothiophenes have been reported over the past three decades, with a focus on their applications in pharmaceuticals, agriculture, pesticides, and dyes. A series of reviews have been published dealing with the latest accomplishments of 2-aminothiophenes^14^. Gewald’s versatile, synthetic method developed by him has brought much attention to the chemistry of 2-aminothiophenes due to the convenience of availability^15^. Gewald method was known as the most well-established approach for preparing 2-amino thiophenes, relies on a three-component reaction involving an α-ketone, an activated nitrile, and elemental sulfur in the presence of a basic catalyst^16–19^.

The core structure is formed in the multi-component reaction between α-ketone or an aldehyde, an activated nitrile, and sulfur. This method has been universally adopted for the synthesis of substituted 2-amino thiophenes, since its introduction in 1961, But still, the research and generation of new compounds with this method is rapidly expanding due to its easy adaptability in the field of pharmaceutical and material chemistry. While the one-pot Gewald synthesis is widely accepted, a step-wise procedure involving the preparation of α,β-unsaturated nitrile through the condensation of α-ketone or aldehyde with an activated nitrile, followed by a base-promoted reaction with sulfur, promises a more comprehensive understanding of the reaction mechanism^14,15,20^. Notably, among thiophene derivatives, 2-amino thiophenes emerge as versatile materials with applications spanning various scientific disciplines. They play pivotal roles in exhibiting potent biological activities, such as serving as allosteric enhancers of adenosine receptors^21^ and glucagon receptor antagonists^22^. Additionally, their applications extend to materials science, including usage in dyes^23^, conductivity-based sensors^24^, and bio-diagnostics^15,25^.

**Fig. 2:**
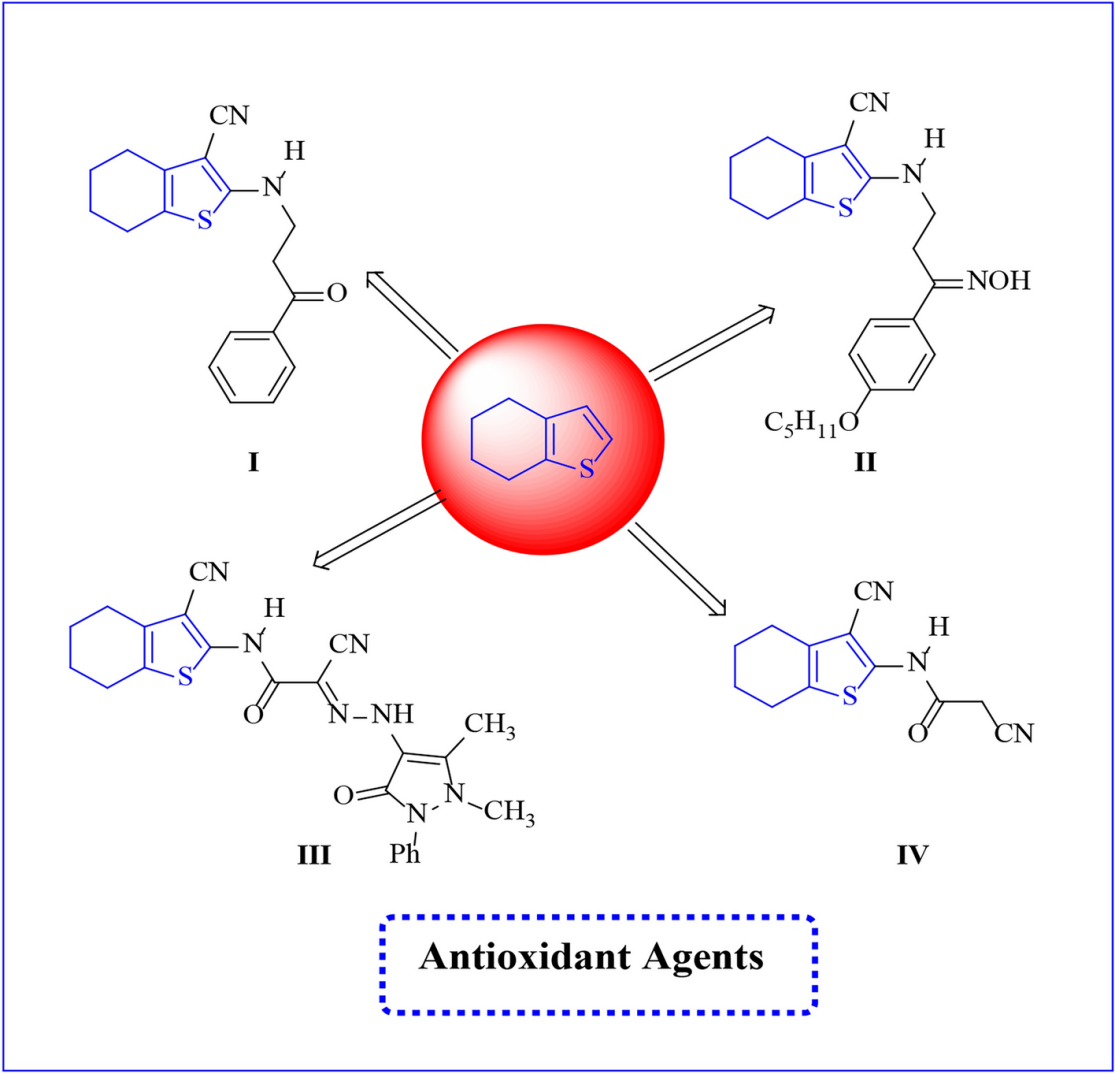
Some reported tetrahydro benzo[*b*]thiophene derivatives such as antioxidant.

Additionally, Tetrahydrobenzo[*b*]thiophene derivatives display a range of biological activities, notably significant anti-inflammatory properties^26^. Also, the compounds **I**, **II**, **III**, and **IV** which containing benzo[*b*]thiophene nucleus exhibit potent antioxidant activity^27,28^ as shown in **(**Fig. 2**)**, demonstrating the capability to inhibit free radical-induced lipid oxidation and the formation of lipid peroxides, with inhibition rates ranging from approximately 19 to 30%^27^.

**Fig. 3:**
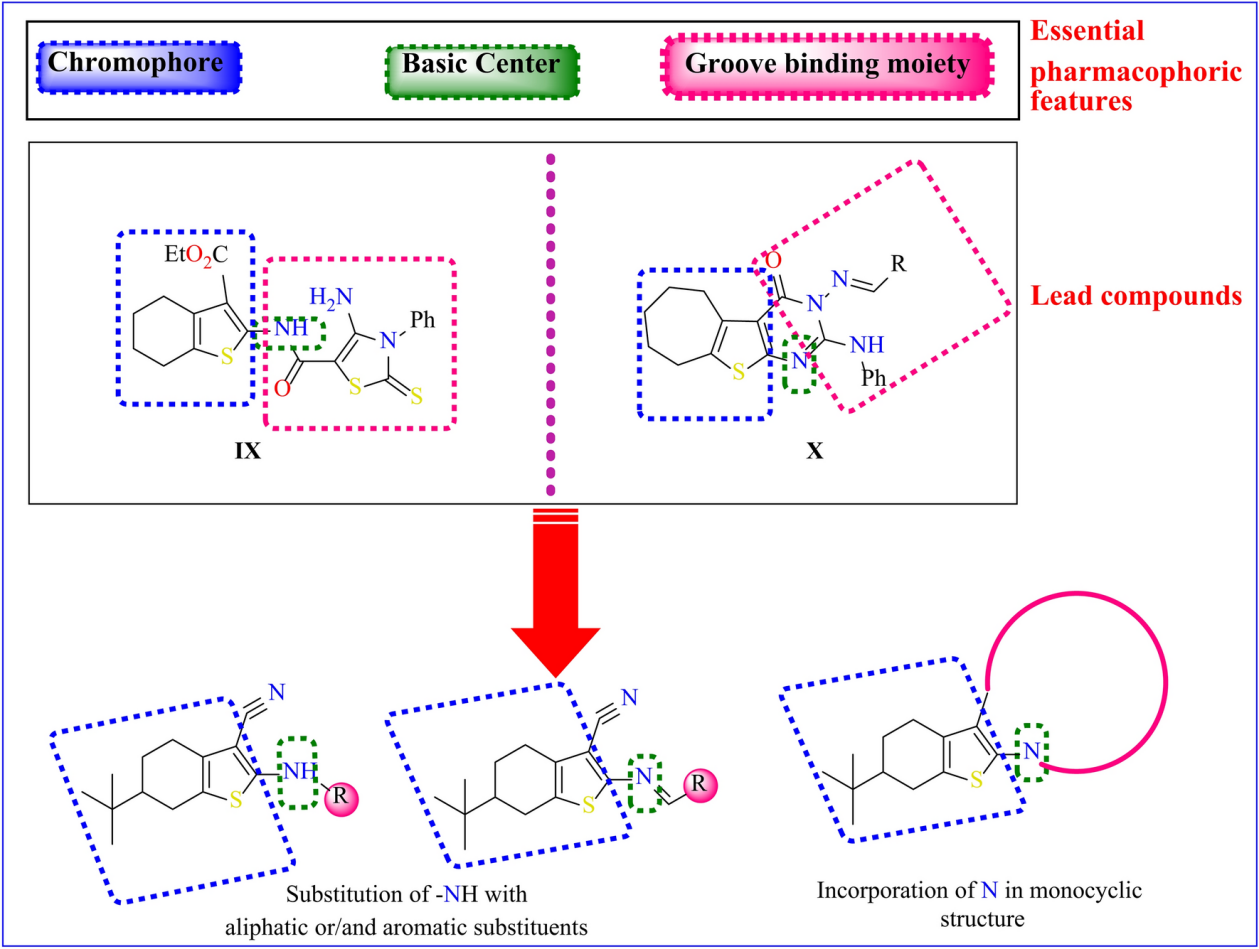
The suggested design rationale of the target candidates 2–21 based on tetrahydro benzo[*b*]thiophene moiety.

As depicted in Fig. 3, tetrahydrobenzo[*b*]thiophene derivative **IX** and tertahydrohepta[*b*]thiophene derivative **X** are recognized as significant fused thiophene derivatives, demonstrating potent antioxidant activities^29,30^. Based on the structural features of the **IX** and **X**, and as an extension of our previous research on the design of new heterocyclic pharmacophores^31–44^. Herein, we describe the design rationale of the target compounds, synthesis of a new group of tetrahydrobenzo*[b]*thiophene congeners, and in vitro antioxidant evaluation according to the phosphomolybdenum method using ascorbic acid as standard. A variety of analytical techniques, such as elemental analysis, ^1^HNMR, IR, and mass spectroscopy, were employed to elucidate the structures of these new heterocyclic derivatives. The total antioxidant capacity (TAC) assay was employed to assess the synthesized compounds’ ability to combat oxidative stress. Additionally, the study delved into the structure–activity relationship (SAR) and explored potential mechanisms of action for these derivatives.

## Results and discussion

### Chemistry

**Scheme 1:**
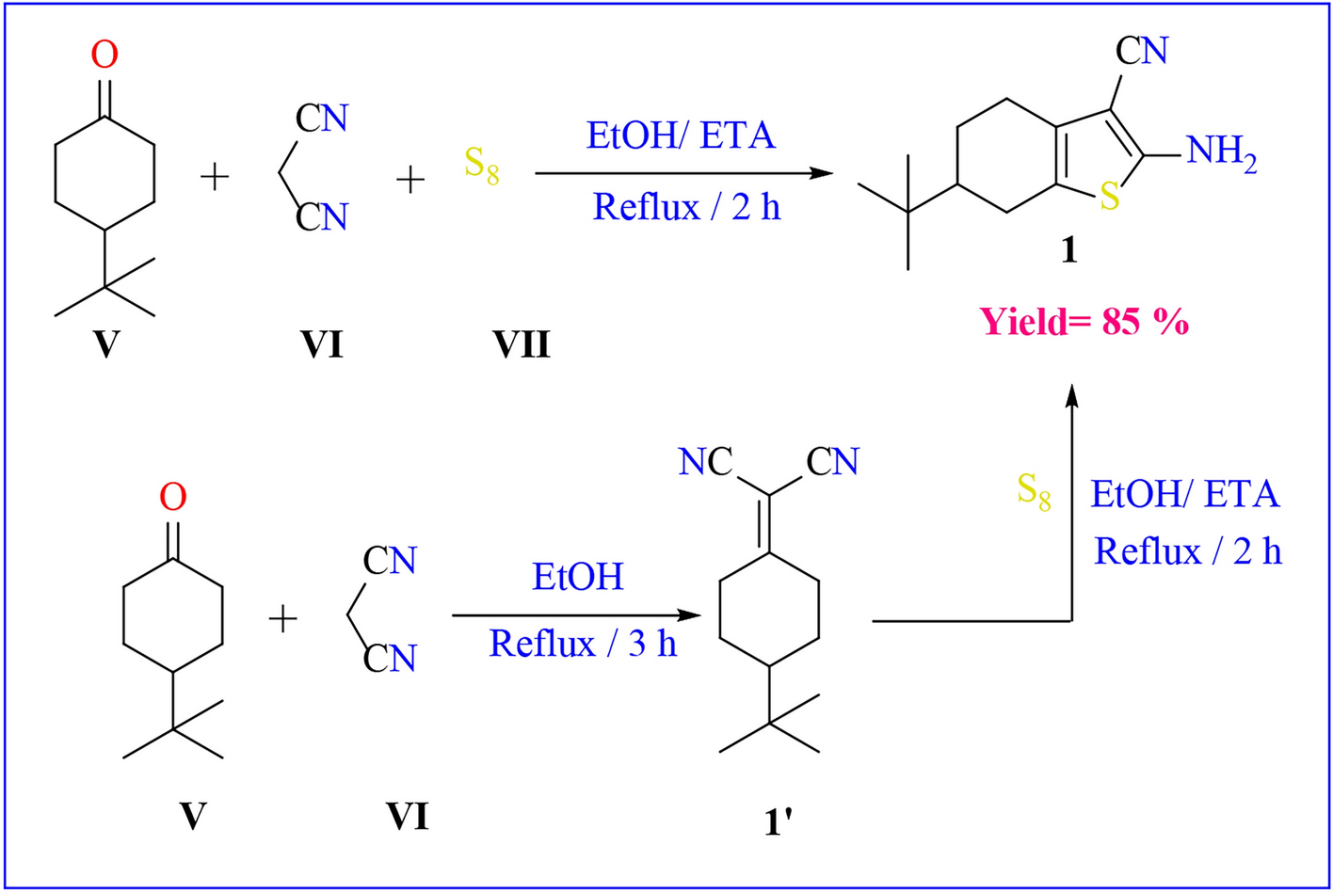
One pot and multi steps Synthesis of starting material.

**Scheme 2:**
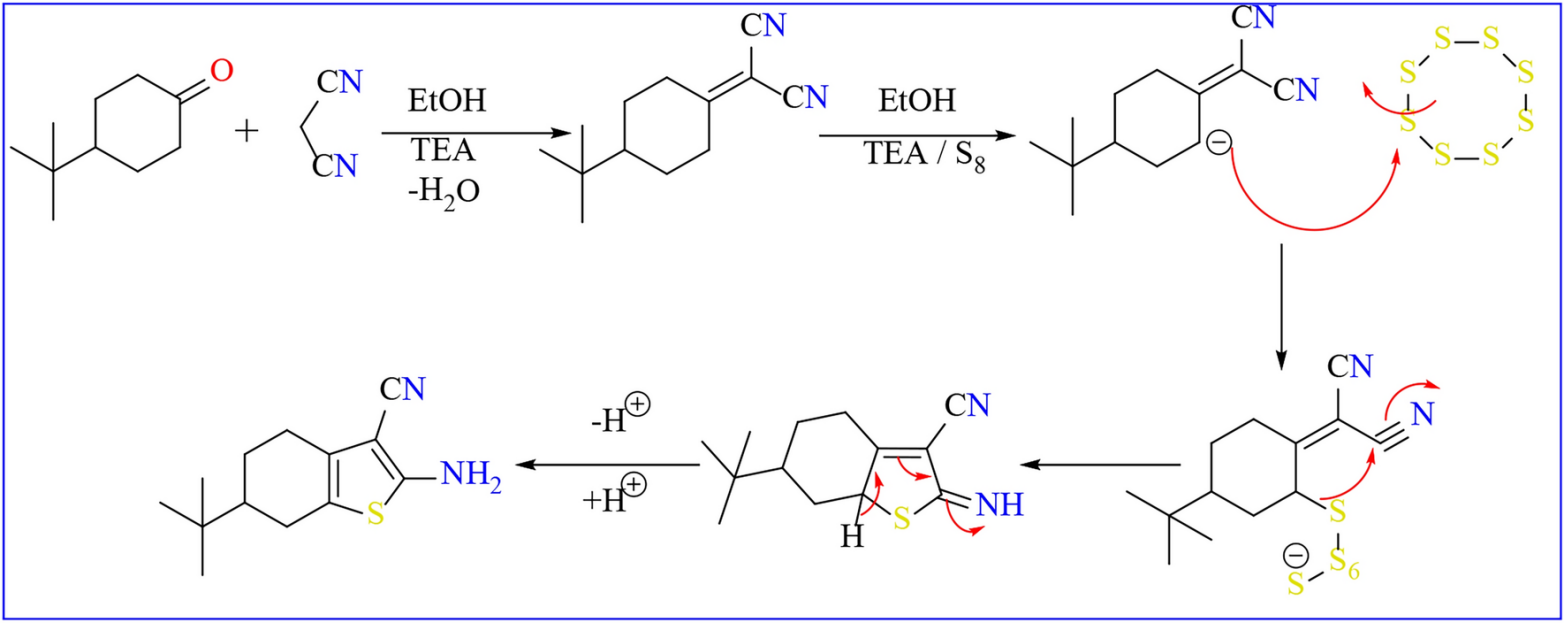
Mechanistic illustration for the formation of compound **1.**

The thiophene ring incorporating an enaminonitrile functional group, specifically 2-amino-6-(tert-butyl)-4,5,6,7-tetrahydrobenzo[*b*]thiophene-3-carbonitrile **1**, was synthesized via the Gewald reaction in a one-pot procedure. This involved heating a mixture of malononitrile, *p*–t-butyl cyclohexanone, and elemental sulfur in absolute ethanol containing a catalytic amount of triethyl amine. The initial compound **1** was obtained through a sequential process, starting with the preparation of α,β-unsaturated nitrile through the condensation of α-ketone with malononitrile **1’**, followed by a base-promoted reaction with sulfur **(**Scheme 1**)**. Compound **1** was previously prepared according to the literature procedure with different conditions and techniques^45^. The FT-IR spectrum of compound **1** showed a strong absorption band at *ν* = 3323–3428 cm^−1^ corresponding to the amino group and band at *ν* = 2201 cm^−1^ for cyano group. Moreover, the ^1^H-NMR spectra showed δ = 0.89 ppm for t-butyl group and D_2_O-exchangeable broad singlet peak due to the amino group at δ = 6.93. A plausible mechanism for the formation of compound **1** is the Michael addition of the active methylene of malononitrile on the α-ketone**,** followed by nucleophilic attack of active methylene of cyclohexanone on sulfur lattice, and then intramolecular nucleophilic attack as shown in **(**Scheme 2**)**.

**Scheme 3:**
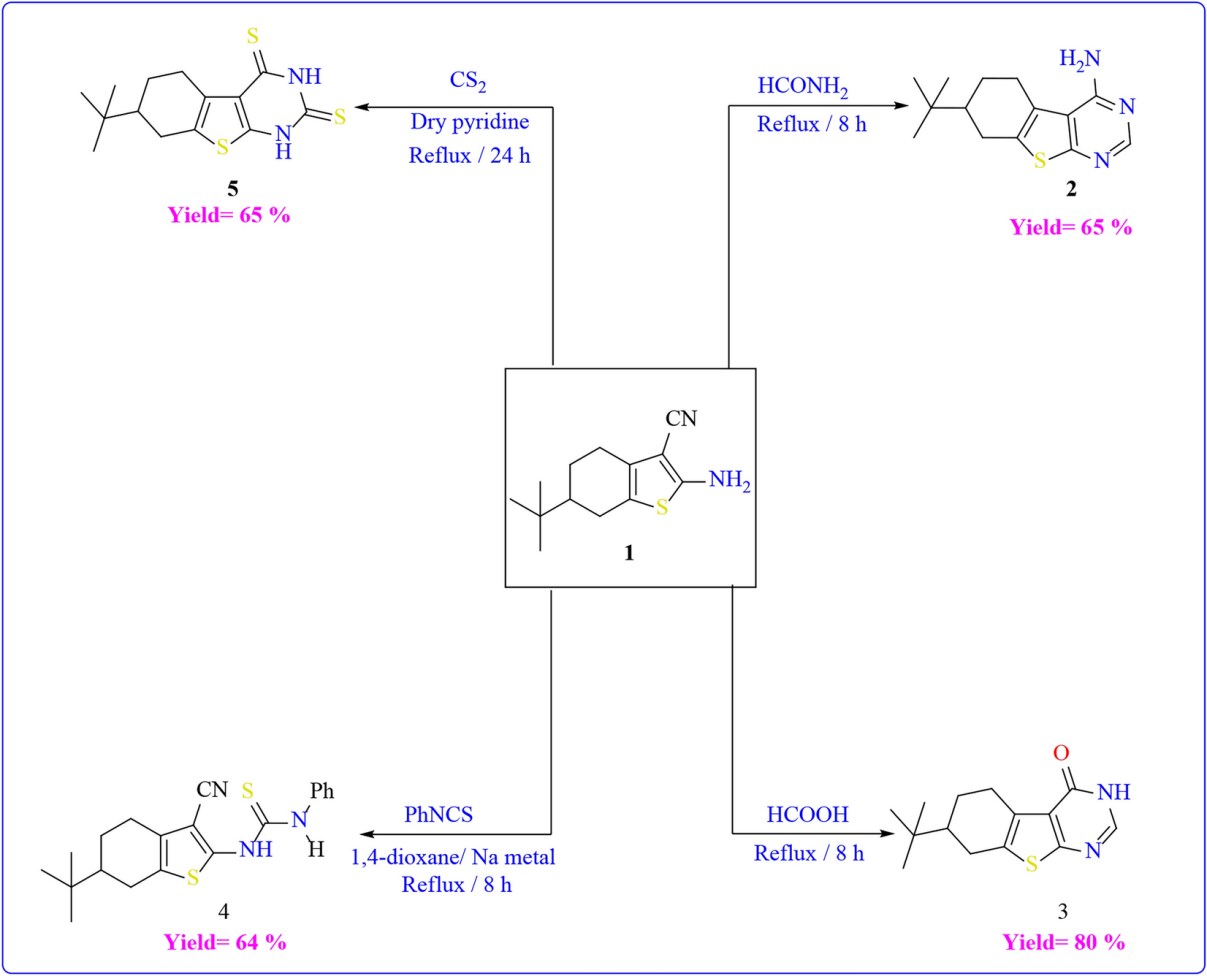
Reactions of compound **1** with different electrophile reagents.

In this study, compound **1** served as a foundational element for generating a variety of heterocyclic frameworks containing NSO heteroatoms. The combination of compound **1** with formamide and/or formic acid resulted in the production of the respective pyrimidine derivatives, namely **2** and **3** (Scheme 3). These compounds had been synthesized earlier following a method outlined in the literature^46^. The FT-IR spectrum of compound **2** indicated the absence of the CN absorption band, replaced by an absorption band at υ = 3313–3424 cm^−1^ attributed to NH_2_ stretching. Additionally, its ^1^H-NMR spectrum exhibited a D_2_O-exchangeable singlet peak at δ = 4.61 ppm, corresponding to the NH_2_ group in the pyrimidine ring, and a singlet peak at δ = 8.32 ppm for the pyrimidine CH proton.

On the other hand, the FT-IR spectrum of compound **3** showcased an absorption band at υ = 1658 cm^−1^, attributed to the C=O in the pyrimidinone ring, along with a newly appearing absorption band at υ = 3157 cm^−1^ due to NH stretching. The ^1^H-NMR spectrum of compound **3** revealed a singlet peak at δ = 7.98 ppm for the pyrimidine CH proton and a D_2_O-exchangeable singlet peak at δ = 12.29 ppm corresponding to the NH group in the pyrimidine ring.

Also, Compound **1** underwent reactions with phenyl isothiocyanate and carbon disulfide, resulting in the formation of the thiophen-2-yl thiourea derivative **4** and the thieno[2,3-d]pyrimidine-2,4(1H,3H)-dithione derivative **5**, respectively (Scheme 3). The FT-IR-spectrum of compound **4** showed the appearance of an absorption band at υ = 3208 and 3288 cm^−1^ due to 2NH stretching, and a band at υ = 2205 cm^−1^ due to the cyano group. In addition, the ^1^H-NMR analysis indicated a D_2_O-exchangeable singlet peak at δ = 8.12 ppm and δ = 8.19 ppm due to the 2NH. However, the FT-IR-spectrum of compound **5** showed the lack of an absorption band for the cyano group and the existence of absorption bands at υ = 3108 and 3379 cm^−1^ due to the 2NH of the pyrimidine ring. Its ^1^H-NMR spectrum exhibited a D_2_O-exchangeable singlet peak at δ = 12.29 ppm and δ = 13.23 ppm due to the 2NH.

**Scheme 4:**
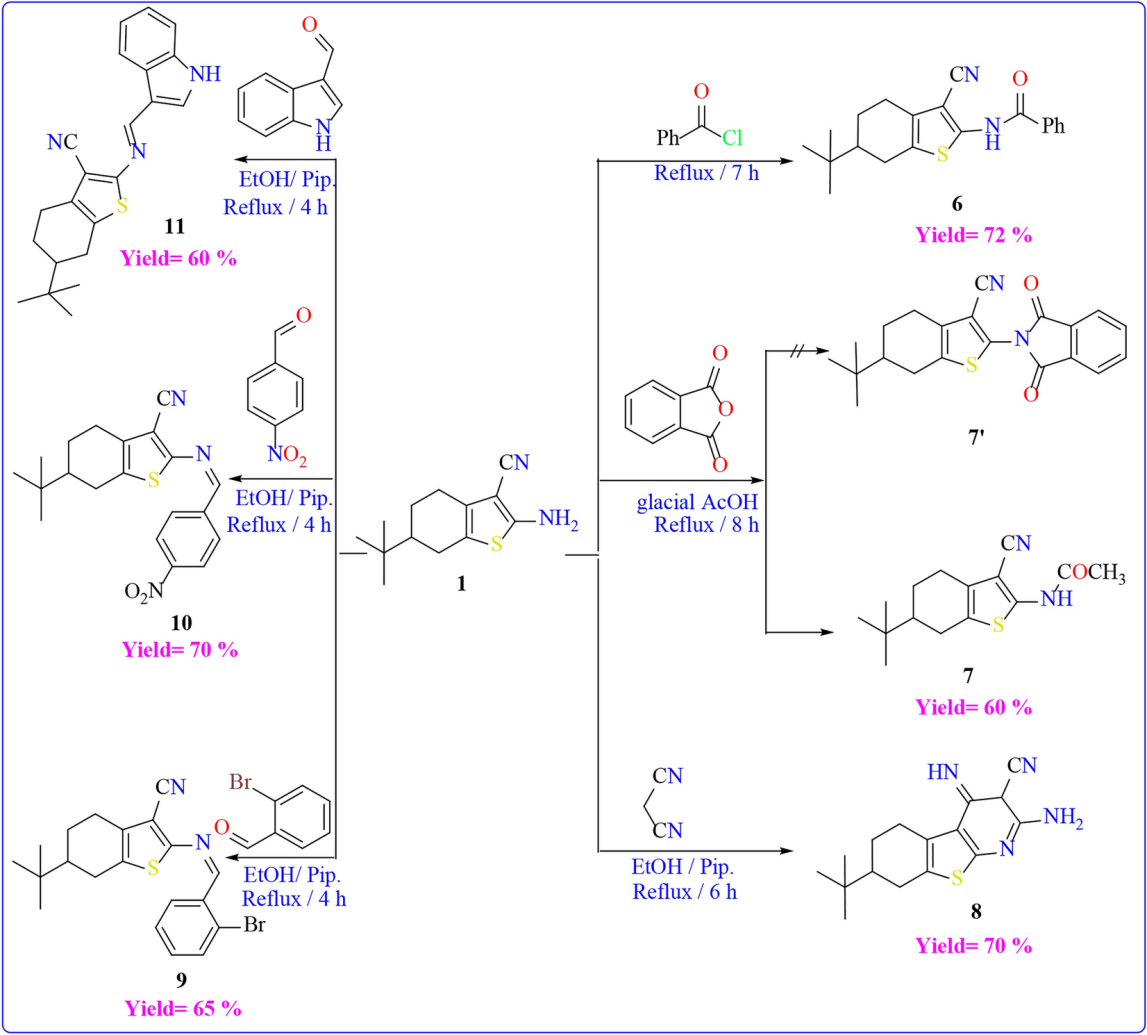
Reactions of compound **1** with different electrophile reagents.

In contrast to this, compound **1** was allowed to react with different electrophilic reagents as outlined in (Scheme 4**)**. First, The acylation of compound **1**, achieved by treating it with benzoyl chloride, resulted in the formation of the corresponding N-(3-cyanothiophen-2-yl)benzamide **6**. The FT-IR spectrum of compound **6** confirmed the emergence of an absorption band at υ = 1667 cm^−1^ due to the carbonyl group of benzamide. In addition, the ^1^H NMR showed multiplet peaks at range δ = 7.55–7.96 ppm due to the aromatic CH proton, and D_2_O-exchangeable singlet peak at δ = 11.70 ppm due to the NH. Upon the reaction of compound **1** with phthalic anhydride in glacial acetic acid, the formation of two potential products was anticipated. However, both elemental analysis and spectral data confirmed the structure of **7** and ruled out the formation of structure **7'** due to the absence of CH aromatic in ^1^H NMR spectrum. The FT-IR spectrum of compound **7** exhibited the existence of an absorption band at υ = 1697 cm^−1^ due to the carbonyl group. Its ^1^H NMR indicated a singlet peak at δ = 2.16 ppm due to the methyl group, and a D_2_O-exchangeable singlet peak at δ = 11.49 ppm due to the NH. The thieno[2,3-b]pyridine-5-carbonitrile derivative **8** was synthesized by subjecting starting material **1** to reflux conditions with malononitrile in absolute ethanol, featuring a catalytic amount of piperidine. The mass spectrum of compound **8** showed its molecular ion peak at m/z = 300 (M^+.^). Moreover, its ^1^H NMR spectrum indicated a singlet peak at δ = 2.17 ppm due to pyridine CH proton, D_2_O-exchangeable singlet peak at δ = 6.92 and δ = 11.50 ppm due to the amino group and the NH group respectively.

Moreover, Compound **1** underwent condensation with *o*-bromo benzaldehyde, *p*-nitro benzaldehyde, and indole-3-carboxaldehyde in an ethanolic solution under reflux conditions, resulting in the formation of the respective Schiff’s bases **9**, **10**, and** 11** respectively. as shown in (Scheme 7). The structures of the synthesized compounds were determined through spectroscopic and elemental analyses. The absence of the characteristic band of the amino group was observed in the FT-IR spectrum of the compounds. Additionally, the FT-IR spectrum of compound **11** exhibited bands at υ = 3328 cm^−1^, indicating the presence of NH groups. The ^1^H-NMR analysis of the synthesized compounds **9**, **10**, and **11** revealed the presence of aromatic CH protons within the range of δ = 7.23–8.40 ppm, in addition to compound **11** its ^1^H-NMR showed a singlet peak at δ = 8.71 ppm due CH proton of pyrrole ring and D_2_O-exchangeable singlet peak at δ = 12.09 ppm corresponding to the NH group.

**Scheme 5:**
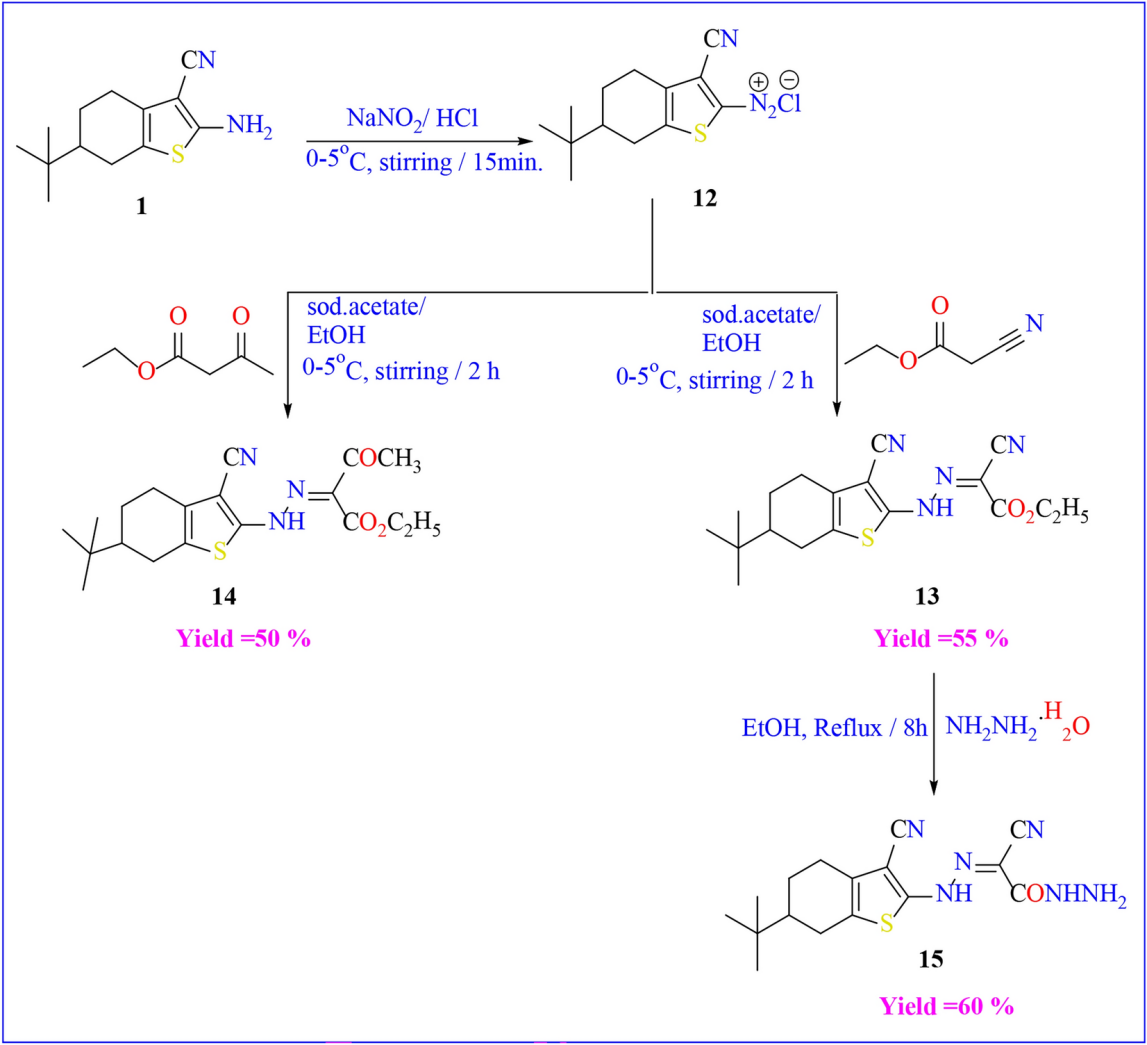
Reactions of diazonium salt of compound 1 with active methylene.

Furthermore, the amino group in compound **1** akin to a primary aromatic amine, demonstrating the ability to produce the corresponding diazonium salt when exposed to nitrous acid within a temperature range of 0 to 5 °C. Additionally, it can engage in coupling reactions with various nucleophilic reagents, including ethyl cyanoacetate and/or ethyl acetoacetate to afford the corresponding coupling products **13** and **14** respectively (Scheme 5). The structures of the synthesized compounds were determined through spectroscopic and elemental analyses. The FT-IR spectrum of compounds **13**, and **14** revealed characteristic bands at υ = 3424 and 3363 cm^−1^ indicating the presence of NH groups respectively. Also appeared absorption bands at υ = 1732 and 1740 cm^−1^ respectively due to the carbonyl groups of esters. The mass spectrum of compounds **13 and 14** showed their molecular ion peak (M^+^) at m/z = 358 and 375 respectively.

The creation of aceto hydrazide derivative **15** was facilitated through the reaction of compound **13** with hydrazine hydrate in absolute ethanol. The structural validation was achieved via spectral analysis. The FT-IR displayed a lack of the characteristic absorption band for the carbonyl ester and the existence of the NH_2_ and NH groups at a range of υ = 3198–3314 cm^-1^. Its ^1^H-NMR exhibited that D_2_O-exchangeable singlet peak at δ = 5.42, 7.64, and 12.41 ppm due to the amino group and the two NH groups respectively.

**Scheme 6:**
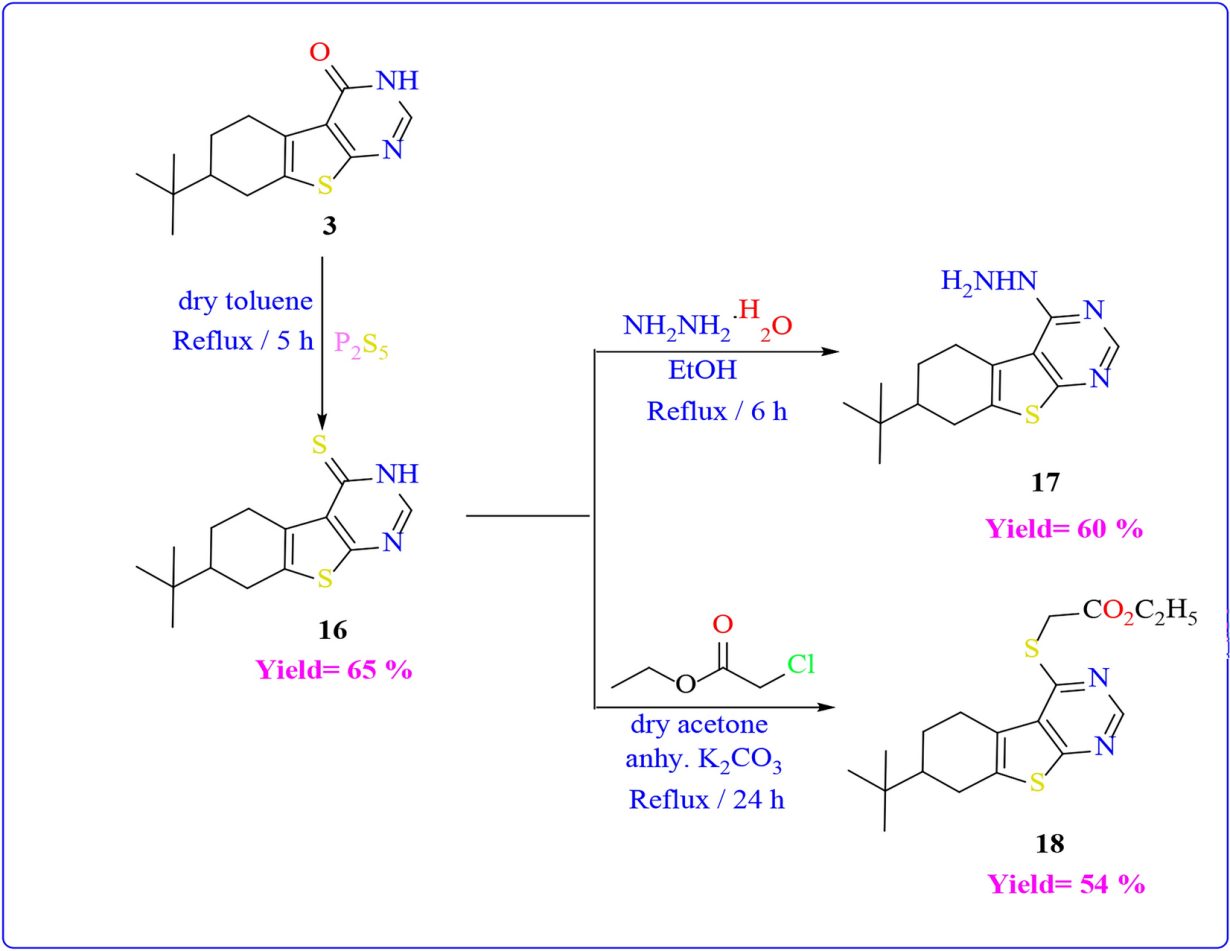
The reactions of compound **16** with various reagents.

Compound **3**, specifically the thieno[2,3-d]pyrimidine-4(3H)-one derivative, served as a valuable key intermediate for the subsequent synthesis of novel pyrimidin-thione derivatives. Firstly, it underwent treatment with phosphorous pentasulfide in dry toluene, yielding the corresponding pyrimidine-4(3H)-thione derivative **16** in only one step. On the other hand, compound **16** has been previously prepared in literature^46^ on two steps by reaction of pyrimidinone **3** with POCl_3_/PCl_5_ followed by reaction the product with thiourea. The FT-IR spectrum of **16** revealed the absence of the carbonyl group of pyrimidinone and the presence of an absorption band at υ = 1362 cm^−1^ corresponding to the C=S group, Meanwhile, its mass spectrum exhibited a peak at m/z = 278 (M^+.^). Subsequently, compound **16** underwent reactions with hydrazine hydrate and ethyl chloroacetate, resulting in the formation of 4-hydrazinylthieno[2,3-d]pyrimidine derivative **17** and ethyl 2-(thieno[2,3-d]pyrimidin-4-ylthio)acetate derivative **18** respectively, as illustrated in (Scheme 6**)**. The FT-IR spectrum of compound **17** showed the bands at υ = 3196 and 3313–3392 cm^−1^, indicating the presence of NH and NH_2_ groups. In addition, the ^1^H-NMR analysis indicated a D_2_O-exchangeable singlet peak at δ = 4.61 ppm and δ = 7.88 ppm due to the NH_2_ and NH group respectively. The FT-IR spectrum of compound **18** revealed absorption bands at υ = 1746 cm^-1^ corresponding to the stretching vibrations of the C=O ester group. Its ^1^H-NMR analysis exhibited a triplet peak at a range δ = 1.17–1.21 ppm for CH_3_ and multiplet peak at a range δ = 4.10–4.16 ppm due to CH_2_–S, and CH_2_ of ester.

**Scheme 7:**
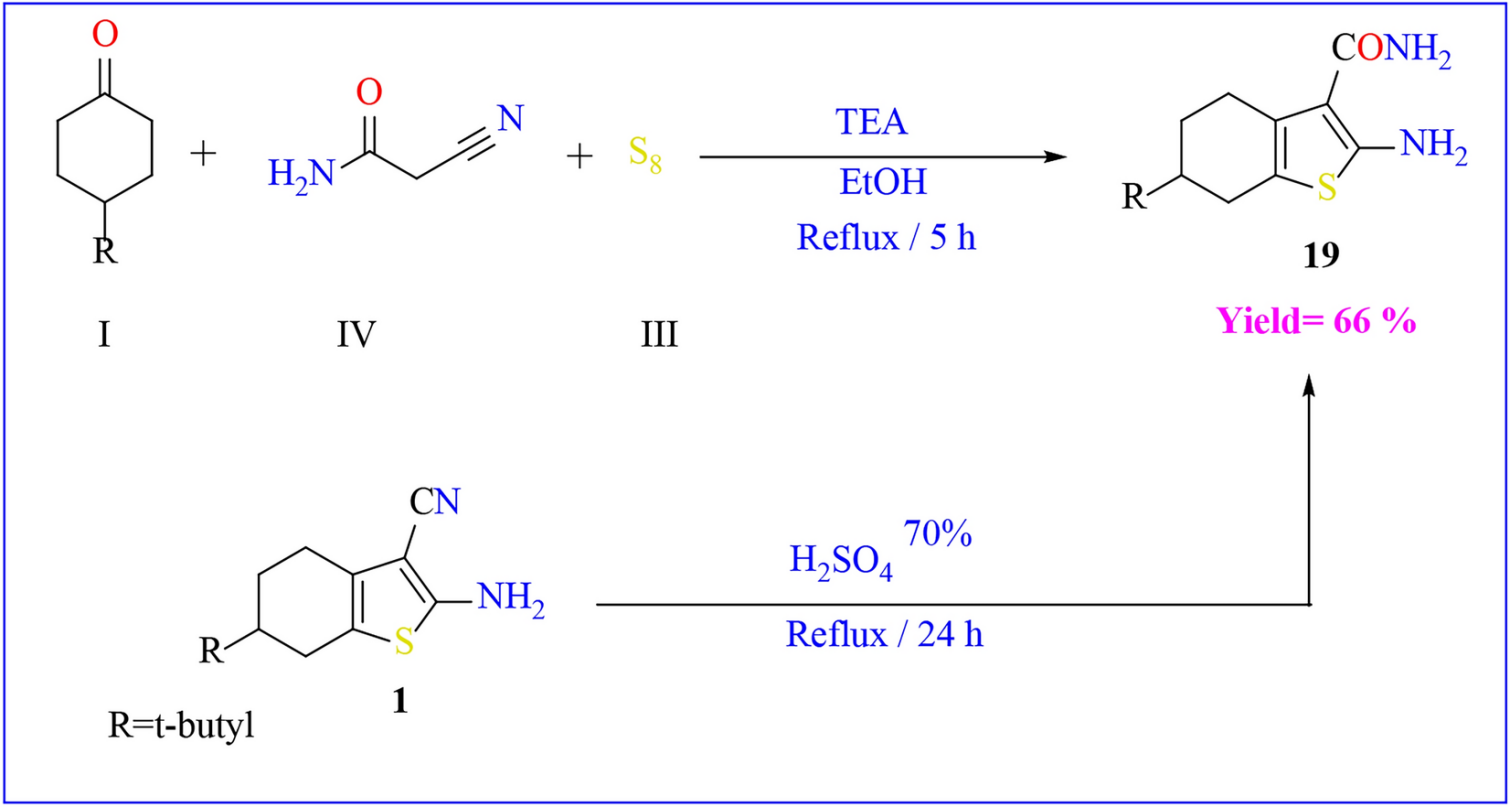
Synthesis of compound **19**.

Additionally, the synthesis of compound **19** is achievable by subjecting compound **1** to hydrolysis in 70% sulfuric acid under reflux on a water bath. An alternative method involves preparing it through the reaction of *p*-t-butyl cyclohexanone with cyano acetamide and a sulfur element in the presence of triethyl amine, as outlined in **(**Scheme 7**)**. The FT-IR spectrum of compound **19** displayed the absence of the cyano group and the appearance of absorption bands at a range of υ = 3322–3497 cm^−1^, indicating the presence of two amino groups. In addition, the ^1^H-NMR analysis indicated a D_2_O-exchangeable singlet peak at δ = 7.19 ppm and δ = 7.88 ppm due to the 2NH_2_ groups.

**Scheme 8:**
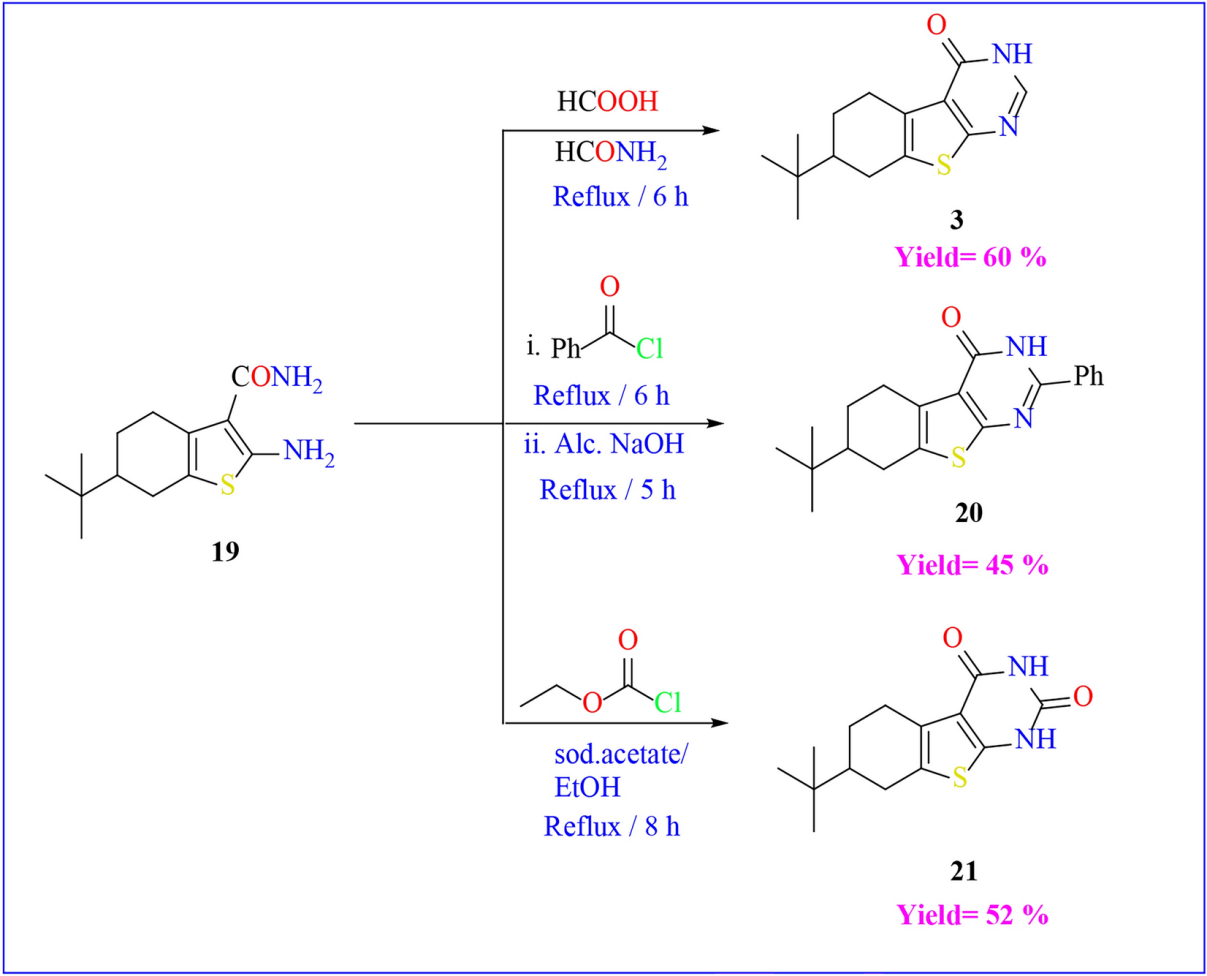
Reactions of compound **19** with different electrophilic reagents.

Treating derivative **19** of 2-aminothiophene-3-carboxamide with formic acid yielded the same product obtained from the reaction of compound **1** with formic acid under reflux conditions **(refer to **Scheme 7**)**. Subsequently, derivative **19** underwent condensation with *p*-nitrobenzaldehyde, benzoyl chloride, and ethyl chloroacetate, resulting in the formation of Schiff base product **20**, pyrimidin-4(3H)-one derivative **21**, and pyrimidine-2,4(1H,3H)-dione derivative **22 (as depicted in **Scheme 8). The identification of the structures of these compounds was confirmed through spectroscopic and elemental analysis. Notably, the FT-IR spectrum of compound **20** exhibited an absence of absorption bands indicative of amino groups. Additionally, its mass spectrum displayed a molecular ion peak at m/z = 518. The FT-IR spectra of compounds **21** and **22** revealed distinctive absorption bands corresponding to carbonyl groups at υ = 1679 and 1657 cm^−1^, and NH groups within the range of υ = 3169–3207 cm^−1^, respectively. In the ^1^H-NMR analysis of compound **21**, a D_2_O-exchangeable singlet peak at δ = 12.99 ppm was observed, attributed to the NH group. Similarly, the ^1^H-NMR spectrum of compound **22** displayed a D_2_O-exchangeable singlet peak at δ = 11.32, and = 12.24 ppm for the 2NH groups.

### Antioxidant activity

#### Determination of total antioxidant capacity (TAC)

The synthesized heterocyclic compounds **1–21** were evaluated for their in *vitro* antioxidant activity. The results of this antioxidant activity were compared with those of ascorbic acid, which was used as a standard reference drug. Compounds **1**,** 16,** and **17** displayed high antioxidant properties, comparable to that of ascorbic acid. Meanwhile, Compounds **2**, **7**,** 9, 10, 11, 15**, **18**, **19**,** 20** and **21** reported moderate antioxidant activities. This suggests the presence of free amino and NH groups in compounds **1, 16, and 17** respectively. Which enhances the antioxidant activity by increasing their hydrogen donor capacity^47,48^. Also, it can be concluded that compounds **1**, **2**, **7**, **9, 10, 11**, **15**, **16**, **17**, **18, 19**, **20** and **21** have potent to moderate antioxidant activity in comparison with ascorbic acid, which might be beneficial to develop new therapeutic agents for the treatment of oxidative stress-associated diseases.

#### Docking study

Molecular docking serves as a prevalent computational technique extensively applied in molecular biology and drug discovery. It is utilized to anticipate how a small molecule ligand binds to a protein receptor. The primary objective of this method is to pinpoint the most energetically advantageous alignment and positioning of the ligand within the receptor’s binding site. This process is vital for understanding the interactions between a ligand and its receptor, which is essential for developing new drugs and designing effective drug therapies by combining structural and computational approaches^48,49^. Furthermore, Kelch-like ECH-associated protein 1 (Keap1) is a therapeutic target for conditions linked to oxidative stress and inflammation. Currently, there are three covalent Keap1-binding drugs available; however, noncovalent inhibitors that disrupt the Keap1 interaction with nuclear factor erythroid 2-related factor 2 (Nrf2) present a promising alternative approach. Both types of inhibitors function by preventing the degradation of Nrf2, which subsequently induces the expression of antioxidant and anti-inflammatory proteins^50^. So, we evaluated the interactions of compounds **1**, **16**, and **17** with the Keap1 (Kelch-like ECH-associated protein 1) protein (PDB: 7C5E), compared to the co-crystallized ligand triethylene glycol (PGE) and ascorbic acid as a reference drug for antioxidants. The docking study of the synthesized compounds was performed with Keap1 to predict if these compounds bind with Keap1 to activate Nrf2. Previously, it has been reported that the inhibition of Keap1 is a strategy that can lead to the activation of Nrf2 (nuclear factor erythroid 2-related factor 2), resulting in increased expression of antioxidant and detoxification genes. Therefore, inhibiting Keap1 activates the Nrf2-mediated antioxidant response, leading to increased expression of genes that protect cells from oxidative stress and promote cellular survival. This pathway is a promising target for therapeutic interventions aimed at enhancing cellular defense mechanisms^51^. The binding free energies (∆G) of synthesized candidates, ascorbic acid, and the co-crystallized ligand (PGE) in comparison to Keap1 have been summarized in (Table 1).

**Table 1:** Docking binding free energies (∆G) of the synthesized candidates with (Keap1) protein.

Compound	Docking score (Kcal/mol)	RMSD	Interaction	Binding site		Distance (Å)	E (kcal/mol)
				Ligand	Receptor		
1	−6.04	1.43	H-donor	S (14)	LEU557	4.12	−0.5
			H-donor	N (18)	ILE 559	3.16	−1.4
			pi-H	5-ring	GLY 605	4.92	−1
16	6.48-	1.51	H-acceptor	S (1)	VAL 418	3.63	−2.5
			H-acceptor	S (1)	VAL 465	3.69	−2.3
			H-acceptor	S (1)	VAL 465	3.93	−0.5
			pi-H	5-ring	ILE 559	4.79	−1.4
17	−6.94	2.18	H-donor	S (24)	VAL 604	3.34	−0.5
			H-donor	N (30)	VAL 512	3.04	−1.2
			pi-H	5-ring	ILE 559	4.9	−1.8
Ascorbic acid	5.21-	1.8	H-donor	O (1)	GLY 367	3.32	−1
			H-donor	O (12)	VAL 512	2.88	−2.3
			H-donor	O (17)	GLY 367	0.133	−1.4
			H-acceptor	O (12)	VAL 465	3.31	−0.8
			H-acceptor	O (14)	VAL 418	0.273	−0.8
			H-acceptor	O (14)	VAL 465	3.22	−0.5
PGE	5.398-	1.47	H-acceptor		VAL 465		−2.1
			H-acceptor		GLY 367		−2.5
			H-acceptor		VAL 606		−1.4
			H-acceptor		ILE 559		−0.5

A re-docking validation step was successfully carried out to guarantee the precision of the docking procedure. The most active candidates (**1, 16,** and **17**) were submitted to a docking process into the Keap1 (7C5E) sites to better understand the pattern by which the studied compounds bonded to the active site. According to the docking score values and binding mode, the affinities of the most potent newly synthesized ligands (**1, 16** and **17**) and ascorbic acid towards the target proteins were contrasted (Table 1) showed that ascorbic acid gives a binding score of −5.21 kcal/mol while compounds **1**, **16** and **17** have a higher binding affinity than ascorbic acid with binding free energy −6.04, −6.48 and −6.94 kcal/mol respectively. The analysis of 2D and 3D interaction figures (Table 2) reveals that compounds **1**, **16**, and **17** exhibit binding with the Keap1 protein at the same site as the co-crystallized ligand (PGE) with different interactions. However, these compounds display distinct interactions. Specifically, the thiophene ring of compounds **16**,** and 17** interacts with Keap1 protein by Pi-H bond with ILE 559 amino acid. Additionally, the amino group of compound **1** binds with the same amino acid ILE 559 by an H-donor bond. Notably, this amino group is the site to which the OH group of the co-crystallized ligand (PGE) binds. This observation helps elucidate the heightened reactivity of the synthesized compound compared to ascorbic acid, as detailed in (Table 1). 

**Table 2:** 2D and 3D pictures representing the interaction of compounds (1, 16, 17 and Ascorbic acid) with (Keap1) binding pockets.

Compound	2D interaction	3D interaction
1		
16		
17		
Ascorpic acid		
PGE		

#### DFT calculations

The chemical and biological characteristics of molecules are fundamentally understood by theoretical DFT calculations^52^. Within DFT, electron density plays a crucial part in determining the chemical reactivity features and holds information about the molecular properties. To comprehend the reactivity and selectivity at a specific atomic site inside a molecule, several DFT-based local reactivity descriptors using electron density were proposed^53,54^. As a result, theoretical computations yield a large number of quanta chemical parameters. The critical chemical activities of the molecules are explained by the computed parameters. Molecule calculations are performed by numerous programs. Gaussian09 RevD.01 and Gauss View 6.0 are these programs^55,56^. Moreover, the global reactivity descriptors can be obtained using the energies of LUMO & HOMO for the most potent antioxidant synthesized thiophene moiety **1**, **16**, **17** were determined by DFT using the B3LYP method along at the 6–31 G (d, p) basis sets^57^ and tabulated in (Table 3).

**Table 3:** Quantum chemical parameters of the selected compounds with density functional theory (DFT) at B3LYP/6-31G (d,p) basis set.

Compound	1	16	17	Ascorbic acid
*E* HOMO (eV)	−5.1200	−5.8667	−5.9358	−6.3715
*E* LUMO (eV)	−0.9583	−1.8190	−1.1104	−1.0304
(∆*E*) Energy gap (eV)	4.1616	4.0476	4.8253	5.3410
(*I*) Ionization energy (eV)	5.1200	5.8667	5.9358	6.3715
(A) Electron affinity (eV)	0.9583	1.8190	1.1104	1.0304
(*η*) Chemical hardness (eV)	2.0808	2.0238	2.4126	2.6705
(χ) Electronegativity (eV)	3.0392	3.8429	3.5231	3.7010
(*S*) Chemical softness (eV^-1^)	0.4805	0.4941	0.4144	0.3744
(*µ*) Electronic chemical potential (eV)	−3.0392	−3.8429	−3.5231	−3.7010
(ω) Electrophilicity index (eV)	2.2195	3.6485	2.5724	2.5645
(µ) Dipole moment (D)	4.4164	1.6939	3.8743	8.2249

**Fig. 4:**
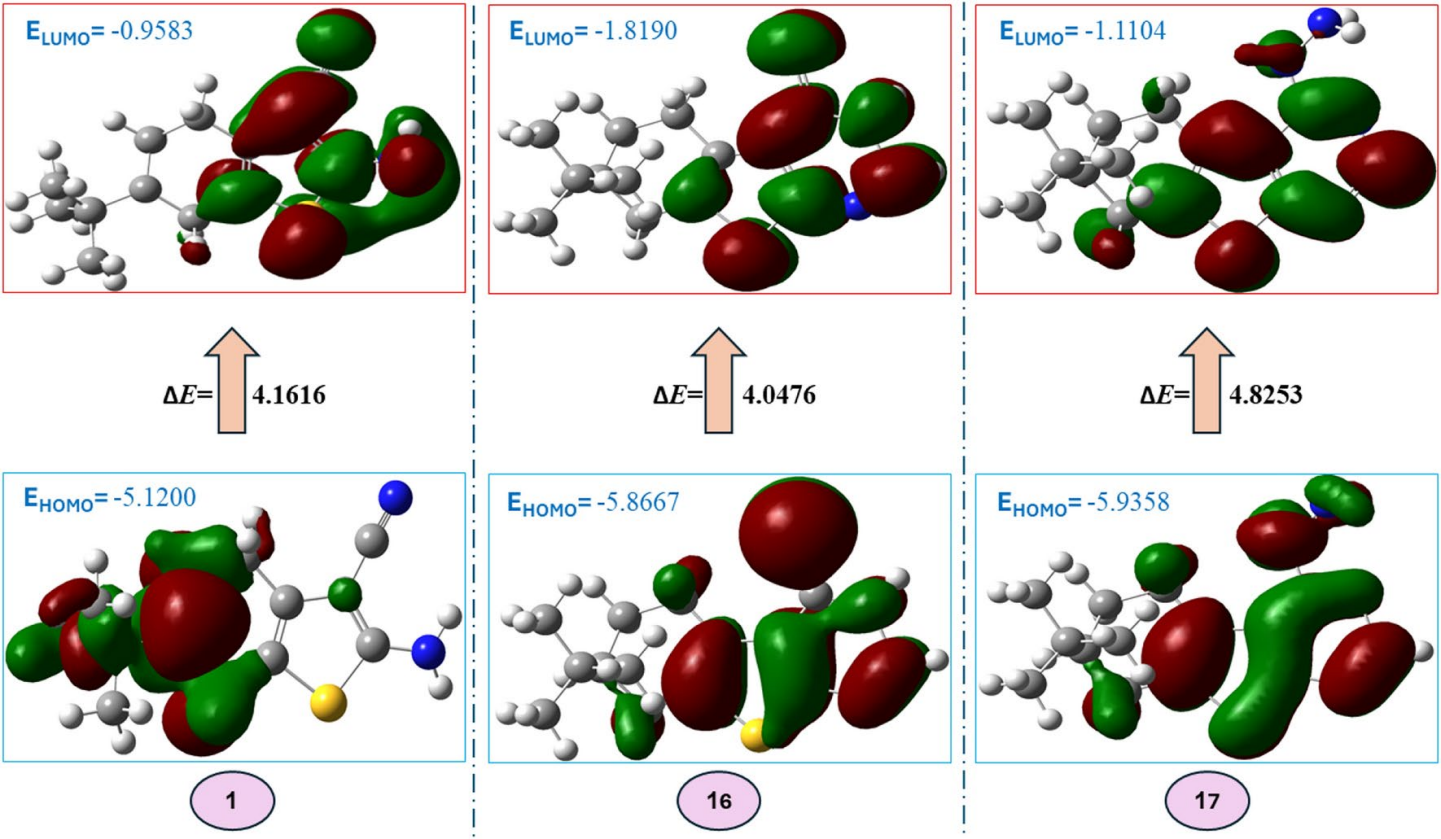
Representation of HOMO and LUMO coefficient distribution and the energy gap in eV of compounds 1, 16, 17.

Furthermore, it has been demonstrated that The Ionization potential (I), Electron affinity (A), Electronegativity (χ), Chemical potential (P), Chemical hardness(η), and Chemical softness (S) are excellent descriptors of biological activity and are thought to be indications of global reactivity^36^. A compound’s chemical stability is largely determined by its energy gap (the difference between its HOMO and LUMO states). Compounds with smaller energy gaps are thought to be more stable, and more likely to donate electrons (HOMO) or accept electrons (LUMO) that making it a potentially effective antioxidant, with lower redox potential which indicates a higher tendency to undergo oxidation or reduction reactions and can readily undergo electronic transitions While this is desirable for antioxidants that need to participate in redox reactions. According to the results listed in Table 3 and shown in (Fig. 4**)**, compound **16** > **1** > **17** > **ascorbic acid** in an antioxidant activity where compounds **16** and **1** are close in their value of **∆E** and all produced compounds have an energy gap smaller than that of ascorbic acid. The activity and Convergence in values for compound **1** and **13** is consistent with the experimental results listed in (Table 4).

**Table 4:**
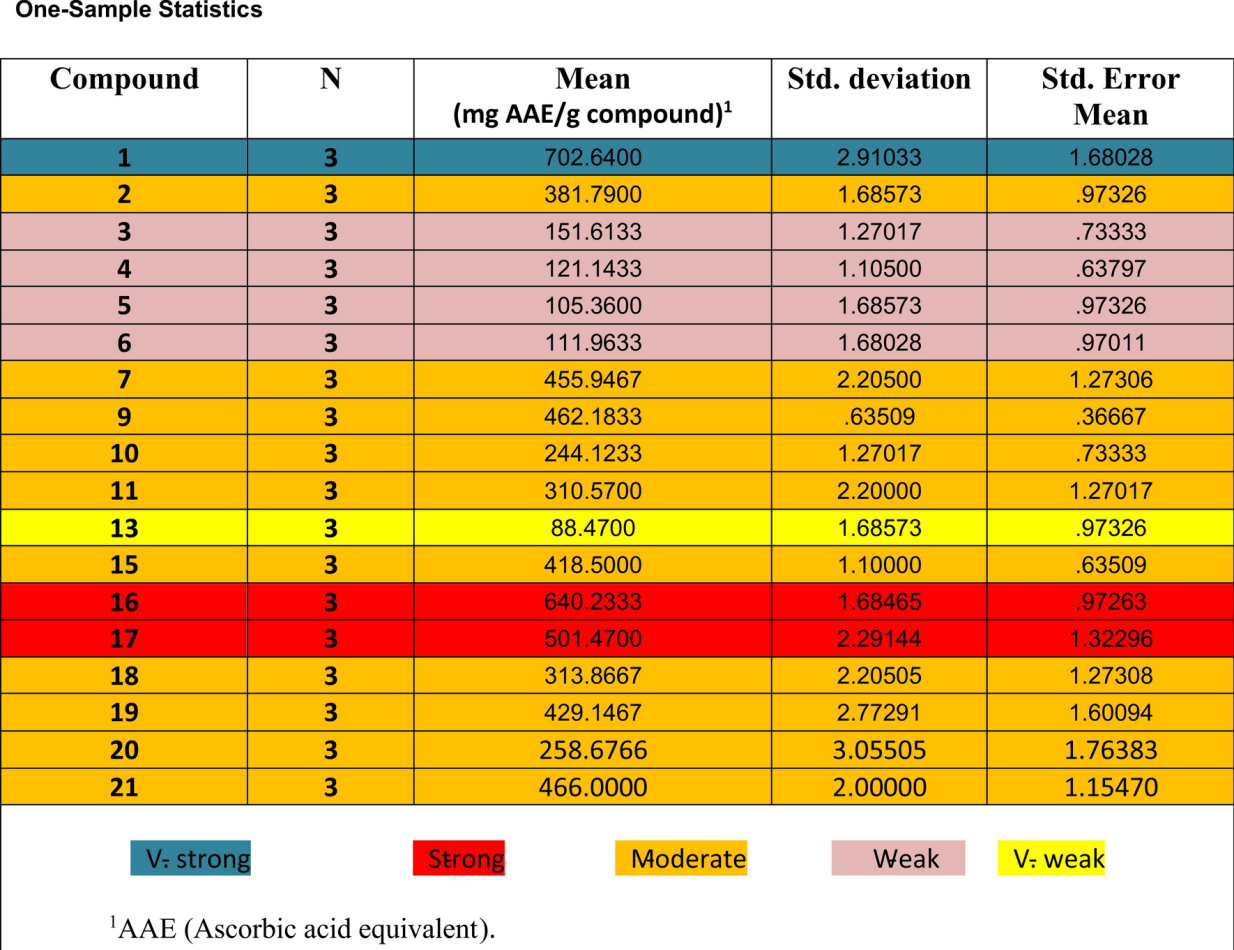
Total antioxidant capacity (TAC) of the synthesized compounds.

Moreover, the ionization potential (*I*) is a crucial parameter for understanding the redox properties and antioxidant potential of a molecule. A lower ionization potential generally indicates a molecule’s enhanced ability to donate electrons, making it more effective in combating oxidative stress. Analyzing the ionization potential, along with other parameters like the HOMO–LUMO gap, provides a comprehensive picture of the electron transfer capabilities of antioxidants. Based on the value of the ionization potential listed in Table 3, it was shown that compound **1** < **16** < **17** in ionization potential and this means that compound **1** > **16** > **17** as antioxidant and this is completely consistent with the practical results in Table 4. In the context of studying antioxidants, chemical softness (S) and chemical hardness (η) can offer valuable insights into the molecule’s response to changes in its environment and its potential as a reactive species scavenger. Values reflect the polarization of the molecule; a higher softness value is more polarizable than the hard one and exhibits higher chemical reactivity due to its lower energy requirement for the excitation process. Consequently, the chemical softness of compounds **1**,** 16,** and** 17** in Table 3** reflects** their elevated chemical softness than ascorbic acid, rendering them more polarized. A reciprocal correlation exists between the chemical potential (*µ*) and electronegativity (χ) concerning their influence on electron donation capability. As the chemical potential rises and electronegativity declines, there is an augmentation in the compound’s capacity to donate electrons. The analysis of (*µ*) and (χ) reveals that compound **1** displays lower electronegativity and greater electron-donating potential than compounds **16** and **17** Which is proven practically in (Table 4).

#### Fukui function

One significant theoretical framework for determining the features of quantum chemistry is provided by density functional theory, or DFT^58,59^. Within DFT, the electron density plays a crucial part in determining the chemical reactivity features and holds information about the molecular properties. To comprehend the reactivity and selectivity at a specific atomic site inside a molecule, several DFT-based local reactivity descriptors including electron density were presented^53,54^. Numerous studies have revealed that Fukui functions^60–64^ are an effective intramolecular reactivity descriptor. They represent the local reactivity of the studied compounds. it is considered that the condensed Fukui functions can give relevant information regarding the reactive sites and the type of biochemical reaction in which they participate. For reactions with radicals’ reaction site indices, fukui function **f**_**k**_^**0**^ is proposed. This function governing radical attack and given by^65^:

f_k_^0^ = [q_k_(N + 1)-q_k_(N-1)]/2.

where:

q_k_(N + 1): electronic population of k atom in an anionic molecule.

q_k_(N − 1): electronic population of k atom in a cationic molecule.

In the present study, the values of fukui function were calculated. If **f**_**k**_^**0**^** > 0,** then the site is prone to radical attack. The positive values of fukui function were collected and tabulated in (Table 5).

**Table 5:** Fukui Function value for a radical attack location.

Compound	Atoms	f_k_^0^
1	C_3_	0.4599035
	C_8_	0.085998
	C_17_	0.0033245
	C_25_	0.1025715
	C_32_	0.1672675
	C_33_	0.00386
16	C_2_	0.1414435
	C_3_	0.050735
	C_6_	0.089216
	C_15_	0.123285
	C_22_	0.1508965
	C_24_	0.0603975
17	C_1_	0.1719425
	C_5_	0.387511
	C_6_	0.0477135
	C_15_	0.0453615
	C_22_	0.081895
	C_23_	0.039035

**Fig. 5:**
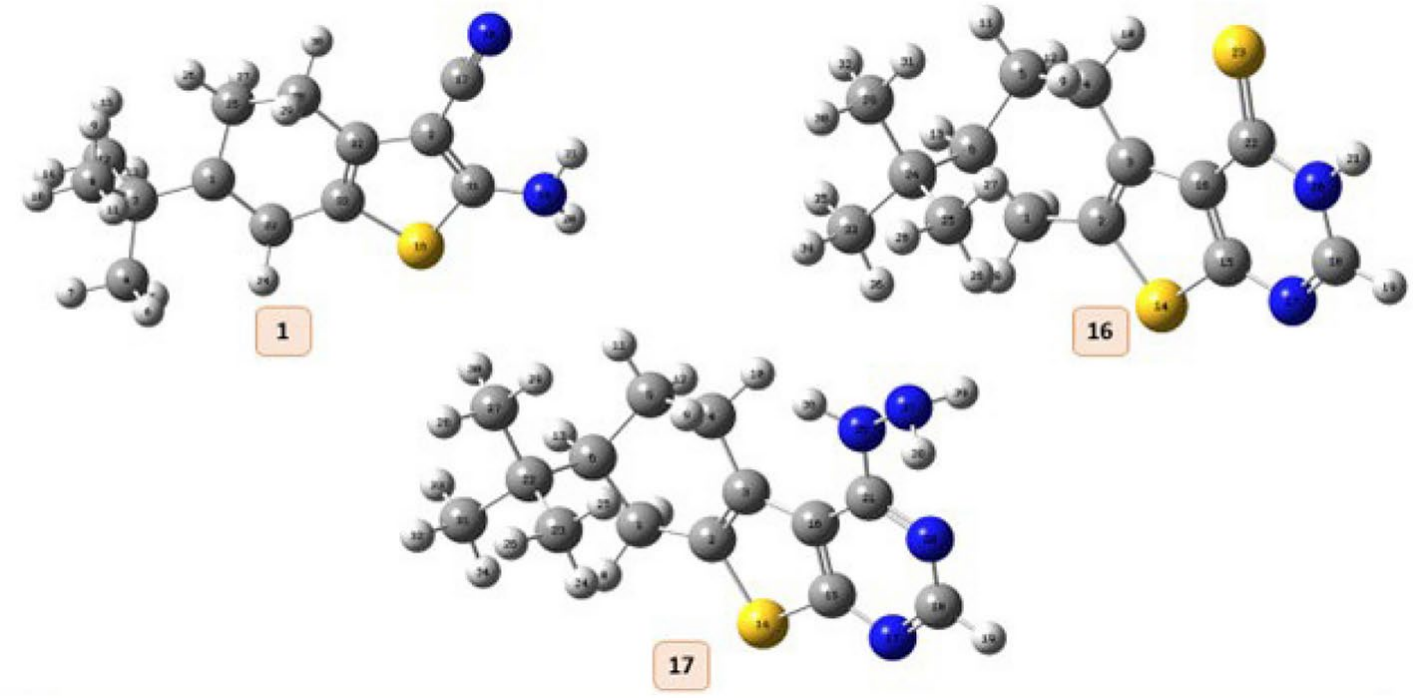
Optimized geometric structure with atoms numbering of compounds 1, 16, and 17.

The calculated **f**_**k**_^**0**^ values predict that the possible sites for the radical attack in compound **1** at C_3_, C_25,_ and C_32_ are labeled in (Fig. 5**)**_._ The computations show that the C_3_ atom has the highest value between all the compounds Which proves the highest practical result of this compound as an antioxidant in (Table 4).

Moreover, it has been observed that C_2_, C_15_ and C_22_ are prone to radical attack in compound **16** because they show a higher positive value than other atoms. Also, compound **17** has been shown high tendency for radical attack, especially at atoms C_1_ and C_5_ Which explains its antioxidant activity_._

#### Structure activity relationship (SAR)

**Fig. 6:**
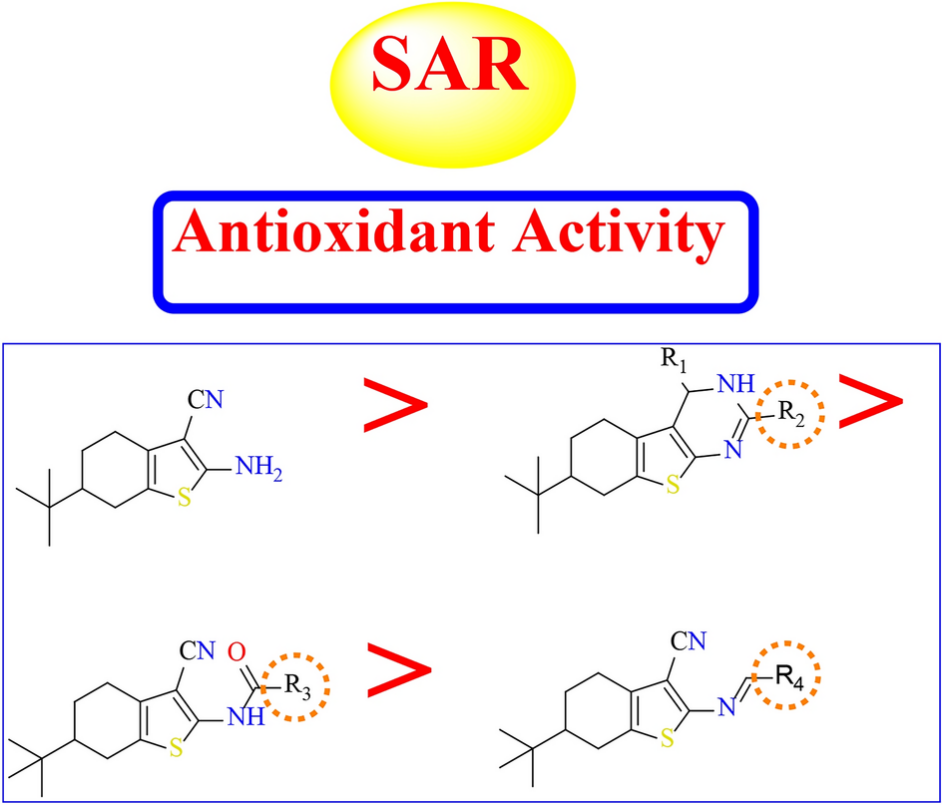
Antioxidant activity of tetrahydro benzo[*b*]thiophene derivatives related to their chemical structures.

Generally, the starting material of tetrahydro benzo[*b*]thiophene-3-carbonitrile was exhibited greater antioxidant activity compared to the other prepared derivatives, as shown in (Fig. 6).

#### For pyrimidine derivatives

It was found that the reactivity of pyrimidine ring as antioxidants was increased when pyrimidine ring was substituted with electro donating group in position four as in compound **16** (R_1_ = SH, R_2_ = H) and in compound **17** (R_1_ = NHNH_2_, R_2_ = H). By replacement of electro donating group to carbonyl group in the pyrimidine ring as in compound **20** (R_1_ = CO, R_2_ = Ph) and in compound **21** (R_1_ = R_2_ = CO) the oxidant activity was decreased. Also, the antioxidant activity was decreased by the replacement of carbonyl group into thiocarbonyl group as in compound **5** (R_1_ = CS, R_2_ = SH).

#### For N-substituted derivatives

It was found that the incorporation of amino group with alkyl as in compound **7** (R_3_ = CH_3_) was stronger in antioxidant activity than aryl group as in compound **6** (R_3_ = Ph). Also, the substitution of amino group with R_3_ = -N = (CN)CONHNH_2_ as in compound **15** was increased the antioxidant activity than the substitution with R_3_ = -N = (CN)CO_2_C_2_H_5_ as in compound **13**.

Furthermore, the condensation of amino group with aryl group containing electro donating group the antioxidant was increased as in compounds **9** (R_4_ = *o*-bromo phenyl) and **11** (R_4_ = 3-indolyl) than compound **10** (R_4_ = *p*-nitro phenyl).

## Experimental

### Materials

*P*-t-butyl cyclohexanone (98%) and malononitrile (99%) were supplied from Acros Organics and Alfa Aesar, respectively. Other chemicals and solvents used in syntheses and analytical assays were of analytical grade and were purchased from Sigma-Aldrich. Solvent drying was carried out according to standard techniques.

### Instruments

Melting points were determined using a Griffin melting point apparatus. Fourier Transform Infrared (FT-IR) spectra were recorded from 400 to 4000 cm^−1^ using a Pye Unicom SP2000 infrared spectrophotometer with the KBr disc technique. Electron ionization mass spectra (EI-MS) were obtained using an AE1MS 902 mass spectrometer. Proton nuclear magnetic resonance (^1^H NMR) spectra were recorded on a Varian Gemini instrument at 400 MHz, and the chemical shifts (δ) were reported in parts per million (ppm) relative to tetramethyl silane (TMS) as an internal standard in deuterated dimethyl sulfoxide (DMSO-d6). Total Antioxidant Capacity Assay is a method used to measure the overall antioxidant capacity of the synthesized compounds. Antioxidants play a crucial role in protecting cells from damage caused by oxidative stress. Elemental analysis (C, H, N, and S) was performed at the Microanalytical Data Center at the Faculty of Science, Cairo University, Egypt. The progress of all reactions was monitored by TLC (thin layer chromatography, Merck) and spots were detected using a UV lamp (254 nm). Compounds **17** and **18** were synthesized previously as reported in the literature^46^.

### Synthetic procedures

#### Synthesis of 2-(4-(tert-butyl)cyclohexylidene)malononitrile (1’)

To a solution of *p*–t-butyl cyclohexanone in absolute ethanol, (0.01 mol, 0.66 mL) of malononitrile was added, and the mixture was heated under reflux for 3 h. The reaction mixture was poured into ice. The formed precipitate was filtered off, dried, and recrystallized from ethanol to give **1’**. Yield (75%); mp_=_ 80–82 °C (ethanol); FT-IR (KBr) (υ cm^−1^):1592 (C=C), 2954 (CH olefinic), 2228(CN); 202(M^+^, 10.12%). Anal. Calcd. For C_13_H_18_N_2_ (202): C, 77.18; H, 8.97; N, 13.85. Found: C, 77.20; H, 9.00; N, 13.83.

#### 2-amino-6-(tert-butyl)-4,5,6,7-tetrahydrobenzo[*b*]thiophene-3-carbonitrile (1)

A mixture of t-butyl cyclohexanone (0.01 mol, 1.54 g), malononitrile (0.01 mol, 0.66 g), and Sulphur element (0.01 mol, 0.32 g) in absolute ethanol (20 mL) containing (0.5 mL) of triethyl amine was heated under reflux condition for 2 h. the reaction mixture was poured into crushed ice / HCl with stirring. The formed precipitate was filtered off, dried, and recrystallized from ethanol to give **1.**

Yield (85%); mp_=_170–172 °C (ethanol); FT-IR (KBr) (υ cm^-1^):1625 (C = C), 2959 (CH olefinic), 2201(CN), 3323–3428 (NH_2_); ^1^H-NMR (DMSO-d_6_) δ_H_(ppm): 0.89 (s, 9H, t-butyl), 1.15–2.47(m, 7H, H-cyclohexane), 6.93(br.s., 2H, NH_2_, D_2_O exchangeable); 234(M^+^, 12.45%).Anal. Calcd. For C_13_H_18_N_2_S (234): C, 66.62; H, 7.74; N, 11.95. Found: C, 66.65; H, 7.71; N, 11.93.

#### 7-(tert-butyl)-6,7,8,9-tetrahydrobenzo[4,5]thieno[3,2-d]pyrimidin-4-amine (2)

Treatment of compound **1** (0.01 mol, 2.34 g) with formamide (20 mL) was heated under reflux for 8 h. The reaction mixture was poured into crushed ice. The formed precipitate was filtered off, dried and recrystallized from benzene to obtain **2.**

Yield (65%); mp _=_ 228–230 °C (benzene); FT-IR(KBr) (υ cm^−1^): 1644 (C = N), 2958 (CH olefinic), 3313–3424 (NH_2_); ^1^H-NMR (DMSO-d_6_) δ_H_ (ppm): 0.93(s, 9H, t-butyl), 1.28–3.10(m, 7H, H-cyclohexane), 4.61 (br.s., 2H, NH_2_, D_2_O exchangeable), 8.32 (s, 1H, H-pyrimidine); MS, m/z(%): 261(M^+^, 14.33%). Anal. Calcd. For C_14_H_19_N_3_S (261): C, 64.33; H, 7.33; N, 16.08. Found: C, 64.30; H, 7.35; N, 16.05.

#### 7-(tert-butyl)-6,7,8,9-tetrahydrobenzo[4,5]thieno[3,2-d]pyrimidin-4(3H)-one (3)

To a solution of compound **1** (0.01 mol, 2.34 g) in formic acid (20 mL) was heated under reflux for 8 h. The reaction mixture was poured into crushed ice and neutralized with sodium carbonate solution. The formed precipitate was filtered off, dried and recrystallized from benzene to obtain **3**.

Yield (80%); mp_=_ 252–254 °C (benzene); FT-IR (KBr) (υ cm^−1^): 1593(C = N), 1658(C=O), 2947 (CH olefinic), 3157(NH); ^1^H-NMR (DMSO-d_6_) δ_H_ (ppm): 0.92 (s, 9H, t-butyl), 1.24–3.20(m, 7H, H-cyclohexane), 7.98 (s, 1H, H-pyrimidinone) 12.29 (br.s, H, NH, D_2_O exchangeable); MS, m/z(%): 262(M^+^, 16.41%). Anal. Calcd. For C_22_H_13_Cl_2_N_3_ (262): C, 64.09; H, 6.92; N, 10.68. Found: C, 64.06; H, 6.90; N, 10.70.

#### 1-(6-(tert-butyl)-3-cyano-4,5,6,7-tetrahydrobenzo[*b*]thiophen-2-yl)-3-phenylthiourea (4)

Treatment of compound **1** (0.01 mol, 2.34 g) with phenyl isothiocyanate (0.01 mol, 1.35 mL) in dioxane (20 mL) containing sodium metal (0.25 g) was heated under reflux 8 h. The reaction mixture was poured into crushed ice and neutralized with hydrochloric acid. The formed precipitate was filtered off, dried, and recrystallized from benzene/ethanol to afford **4**.

Yield (64%), mp _=_ 210–212 °C, (benzene/ethanol); FT-IR (KBr) (υ cm^−1^): 1613(C = C),1242(C = S), 2205(CN), 2956 (CH olefinic), 3208(NH), 3288(NH); ^1^H-NMR (DMSO-d_6_) δ_H_ (ppm): 0.93 (s, 9H, t-butyl), 1.23–2.95 (m, 7H, H-cyclohexane), 7.24–7.77(m, 5H, H-Ar), 8.12 (br.s, H, NH, D_2_O exchangeable), 8.19 (br.s, H, NH, D_2_O exchangeable); MS, m/z: 369(M^+^, 79.91%). Anal. Calcd. For C_20_H_23_N_3_S_2_ (369): C, 65.00; H, 6.27; N,11.37. Found: C, 65.03; H, 6.25; N, 11.40.

#### 7-(tert-butyl)-5,6,7,8-tetrahydrobenzo[4,5]thieno[2,3-d]pyrimidine-2,4(1H,3H)-dithione (5)

The reaction of compound **1** (0.01 mol, 2.34 g) with carbon disulfide (5 mL) in dry pyridine (20 mL) was heated under reflux on a water bath for 24 h. Upon cooling, the precipitated solid was collected, dried, and recrystallized from benzene to afford **5**.

Yield (65%), mp _=_ 290–292 °C, (benzene); FT-IR (KBr) (υ cm^−1^): 1600(C=C), 1235 (C=S), 3108(NH), 3379(NH); ^1^H-NMR (DMSO-d_6_) δ_H_ (ppm): 0.92 (s, 9H, t-butyl), 1.24–3.20 (m, 7H, H-cyclohexane), 12.29 (br.s, H, NH, D_2_O exchangeable), 13.23 (br.s, H, NH, D_2_O exchangeable); MS, m/z: 310(M^+.^, 33.58%). Anal. Calcd. For C_14_H_18_N_2_S_3_ (341): C, 54.16; H, 5.84; N, 9.02. Found: C, 54.13; H, 5.81; N, 9.05.

#### N-(6-(tert-butyl)-3-cyano-4,5,6,7-tetrahydrobenzo[*b*]thiophen-2-yl)benzamide (6)

A mixture of compound **1** (0.01 mol, 2.34 g) and benzoyl chloride (20 mL) was heated under reflux for 7 h. After evaporation of the solvent, wash the formed precipitate with petroleum ether (80C-100C) and recrystallized from benzene to give **6**.

Yield (72%), mp_=_ 180–182 °C, (benzene); FT-IR (KBr) (υ cm^-1^):1667 (C = O), 2216 (CN), 3245 (NH); ^1^H-NMR (DMSO-d_6_) δ_H_ (ppm): ^1^H-NMR (DMSO-d_6_) δ_H_ (ppm): 0.93 (s, 9H, t-butyl), 1.27–2.75 (m, 7H, H-cyclohexane), 7.55–7.96 (m, 5H, H-Ar), 11.70 (br.s, H, NH, D_2_O exchangeable); MS, m/z: 338(M^+.^, 11.23%). Anal. Calcd. For C_20_H_22_N_2_OS (338): C, 70.97; H, 6.55; N,8.28. Found: C, 80.00; H, 6.52; N, 8.25.

#### 6-(tert-butyl)-2-(1,3-dioxoisoindolin-2-yl)-4,5,6,7-tetrahydrobenzo[*b*]thiophene-3-carbonitrile (7)

A mixture of compound **1** (0.01 mol, 2.34 g) and phthalic anhydride (0.01 mol, 1.48 g) in glacial acetic acid (20 mL) was refluxed for 8 h. The reaction mixture was poured into crushed ice. The formed precipitate was filtered off, dried, and recrystallized from toluene to afford **7**.

Yield (60%); mp = 242–244 °C (toluene); FT-IR (KBr) (υ cm^-1^): 1697 (C = O), 2217 (CN), 2948 (CH olefinic), 3225 (NH); ^1^H-NMR (DMSO-d_6_) δ_H_ (ppm): 0.90 (s, 9H, t-butyl), 1.22–2.66 (m, 7H, H-cyclohexane), 2.16 (s, 3H, CH_3_), 11.49 (br.s, H, NH, D_2_O exchangeable); MS, m/z: 276 (M^+.^, 21.22%). Anal. Calcd. For C_15_H_20_N_2_OS (276): C, 65.18; H, 7.29; N, 10.14. Found: C, 65.15; H, 7.31; N, 10.11.

#### 2-amino-7-(tert-butyl)-4-imino-3,4,5,6,7,8-hexahydrobenzo[4,5]thieno[2,3-b]pyridine-3-carbonitrile (8)

To a solution of compound **1** (0.01 mol, 2.34 g) in absolute ethanol (25 mL) containing drops of piperidine (3 drops), malononitrile (0.1 mol, 0.66 g) was added and then the reaction mixture was allowed to reflux for 7 h. the reaction mixture was poured into crushed ice/ HCl. The formed precipitate was filtered off, dried and recrystallized from ethanol to obtain **8**.

Yield (70%); mp = 230–232 °C (ethanol); FT-IR (KBr) (υ cm^-1^): 1625 (C=N), 2215 (CN), 3221 (NH), 3268–3321 (NH_2_); ^1^H-NMR (DMSO-d_6_) δ_H_ (ppm): 0.88–0.90 (s, 9H, t-butyl), 1.04–2.67 (m, 7H, H-cyclohexane), 2.17 (s, 1H, CH pyridine), 6.92 (br.s, 2H, NH_2_, D_2_O exchangeable), 11.50 (br.s, H, NH, D_2_O exchangeable); MS, m/z: 300(M^+.^, 13.65%). Anal. Calcd. For C_16_H_20_N_4_S (300): C, 63.97; H, 6.71; N, 18.65. Found: C, 64.00; H, 6.69; N, 18.67.

#### General method for compounds 9, 10 and 11

To a hot solution of compound **1** (0.01 mol, 2.34 g) in ethanol (20 mL) containing a few drops of piperidine (5 drops), *o*-bromo benzaldehyde, and /or *p*-nitro benzaldehyde and/ or indole-3-carboxaldehyde (0.01 mol) was added, and the reaction mixture was heated under reflux for 4 h. The reaction mixture was poured into crushed ice and neutralized with hydrochloric acid. The formed precipitate was filtered off, dried, and recrystallized from proper organic solvent to afford the corresponding Schiff’s base **9, 10, and 11.**

#### 3-((2-bromobenzylidene)amino)-6-(tert-butyl)-4,5,6,7-tetrahydrobenzo[*b*]thiophene-2-carbonitrile (9)

Yield (65%); mp_=_162–164 °C (toluene); FT-IR (KBr) (υ cm^−1^): 1582(C=C), 1629(C=N), 2219(CN); ^1^H-NMR (DMSO-d_6_) δ_H_ (ppm): 0.93 (s, 9H, t-butyl), 1.27–2.83(m, 7H, H-cyclohexane), 7.50–8.16 (m, 4H, H-Ar), 8.76(s, 1H, olefinic N=CH); MS, m/z: 401(M^+.^, 2.92%). Anal. Calcd. For C_20_H_21_BrN_2_S (401): C, 59.85; H, 5.27; N, 6.98. Found: C, 59.82; H, 5.30; N, 6.95.

#### 6-(tert-butyl)-3-(4-nitrostyryl)-4,5,6,7-tetrahydrobenzo[*b*]thiophene-2-carbonitrile (10)

Yield (70%); mp_=_ 216–218 °C (ethanol); FT-IR (KBr) (υ cm^-1^): 1557(C = C), 1598(C = N), 2218(CN); ^1^H-NMR (DMSO-d_6_) δ_H_ (ppm): 0.88–0.93 (s, 9H, t-butyl), 1.29–2.84(m, 7H, H-cyclohexane), 8.19–8.21 (d, 2H, H-Ar), 8.36–8.38 (d, 2H, H-Ar), 8.77(s, 1H, olefinic N = CH); MS, m/z: 367(M^+^, 34.65%). Anal. Calcd. For C_20_H_21_N_3_O_2_S (367): C, 65.37; H, 5.76; N, 11.44. Found: C, 65.40; H, 5.73; N, 11.46.

#### 3-(((1H-indol-3-yl)methylene)amino)-6-(tert-butyl)-4,5,6,7-tetrahydrobenzo[*b*]thiophene-2-carbonitrile (11)

Yield (60%); mp _=_ 210–212 °C (toluene); FT-IR (KBr) (υ cm^−1^): 1564(C=C), 1596(C=N), 2212(CN), 3328(NH); ^1^H-NMR (DMSO-d_6_) δ_H_ (ppm): 0.88–0.93 (s, 9H, t-butyl), 1.29–2.75(m, 7H, H-cyclohexane), 7.23–8.40 (m, 5H, H-Ar), 8.71(s, 1H, olefinic N = CH), 12.09 (br.s., H, NH_,_ D_2_O exchangeable); MS, m/z: 361(M^+.^, 25.53%). Anal. Calcd. For C_22_H_23_N_3_S (361): C, 73.09; H, 6.41; N, 11.62. Found: C, 73.11; H, 6.44; N, 11.65.

#### General method for compounds 13 and 14

The corresponding diazonium chloride **12** was prepared in situ from 2-aminothiophene-3-carbonitrile derivative **1** (0.01 mol, 2.34 g) in conc. HCl (10 mL) and cold solution of sodium nitrite (0.01 mol, 0.69 g in 15 mL of H_2_O) with continuous stirring. To cold mixture of ethyl cyanoacetate (0.01 mol, 1.13 mL) and/or ethyl acetoacetate (0.01 mol, 1.30 mL) in 20 mL ethanol and sodium acetate (0.02 mol, 1.6 g), a cold aqueous solution of diazonium salt **12** was added dropwise with stirring at 0-5C for 2 h. The solid product obtained was filtered off, dried, and recrystallized from benzene to afford compounds **13 and 14** respectively.

#### Ethyl-2-(2-(6-(tert-butyl)-3-cyano-4,5,6,7-tetrahydrobenzo[*b*]thiophen-2-yl)hydrazono)-2-cyanoacetate (13)

Yield (55%); mp_=_174–176 °C (benzene); FT-IR (KBr) (υ cm^-1^):1622(C=N), 1732(C=O), 2224(CN), 3424(NH); MS, m/z: 358(M^+^, 12.41%). Anal. Calcd. For C_18_H_22_N_4_O_2_S(358) : C, 60.31; H, 6.19; N, 15.63. Found: C, 60.35; H, 6.21; N, 15.65.

#### Ethyl-2-(2-(6-(tert-butyl)-3-cyano-4,5,6,7-tetrahydrobenzo[*b*]thiophen-2-yl)hydrazono)-3-oxobutanoate (14)

Yield (50%); mp_=_108–110 °C (benzene); FT-IR (KBr) (υ cm^-1^): 1632(C=N), 1696(C=O), 1740(C=O), 2225(CN), 3363(NH); MS, m/z: 375(M^+^, 25.38%). Anal. Calcd. For C_19_H_25_N_3_O_3_S (375): C, 60.78; H, 6.71; N, 11.19. Found: C, 60.80; H, 6.73; N, 11.21.

#### N-(6-(tert-butyl)-3-cyano-4,5,6,7-tetrahydrobenzo[*b*]thiophen-2-yl)-2-hydrazinyl-2-oxoacetohydrazonoyl cyanide (15)

A mixture of compound **13** (0.01 mol, 3.58 g), and hydrazine hydrate (0.02 mol, 1 mL) in absolute ethanol (20 mL) was heated under reflux for 6 h. The reaction mixture was cooled, and the formed precipitate was filtered off, dried, and recrystallized from ethanol to afford **15**.

Yield (60%); mp_=_ 156–158 °C (ethanol); FT-IR (KBr) (υ cm^-1^):1586 (C = N), 1623 (C = O), 2199 (CN), 3198–3314 (2NH, NH_2_); ^1^H-NMR (DMSO-d_6_) δ_H_ (ppm): 0.89 (s, 9H, t-butyl), 1.15–2.47 (m, 7H, H-cyclohexane), 5.42 (br.s, 2H, NH_2_, D_2_O exchangeable), 7.64 (br.s, 1H, NH, D_2_O exchangeable), 12.41 (br.s, 1H, NH, D_2_O exchangeable); MS, m/z: 344(M^+^, 42.12%). Anal. Calcd. For C_16_H_20_N_6_OS(344): C, 55.79; H, 5.85; N, 24.40. Found: C, 55.76; H, 5.81; N, 24.43.

#### 7-(tert-butyl)-6,7,8,9-tetrahydrobenzo[4,5]thieno[3,2-d]pyrimidine-4(3H)-thione (16)

The reaction of compound **3** (0.01 mol, 2.62 g) with phosphorus pentasulfide (0.01 mol, 4.44 g) in dry toluene was heated under reflux at 5 h and left to cool. The formed precipitate was collected and recrystallized from ethanol to afford compound **16**.

Yield (65%); mp_=_236–238 °C (ethanol); FT-IR (KBr) (υ cm^−1^): 1206(C=S), 1617(C=N), 3125(NH); MS, m/z: 278(M^+^, 21.73%). Anal. Calcd. For C_14_H_18_N_2_S_2_ (278): C, 60.39; H, 6.52; N, 10.06. Found: C, 60.41; H, 6.55; N, 10.09.

#### 7-(tert-butyl)-4-hydrazinyl-6,7,8,9-tetrahydrobenzo[4,5]thieno[3,2-d]pyrimidine (17)

To a solution of compound **16** (0.1 mol, 2.78 g) in 20 mL absolute ethanol, hydrazine hydrate (2 mL) was added and refluxed for 6 h. After cooling, the reaction mixture was poured into ice/ hydrochloride acid then the formed solid was filtered off, dried, and recrystallized from toluene to give compound **17**.

Yield (60%); mp _=_ 180–182 °C (ethanol); FT-IR (KBr) (υ cm^-1^): 1571(C = C), 1629 (C = N), 3196(NH), 3313–3392 (NH_2_); ^1^H-NMR (DMSO-d_6_) δ_H_ (ppm): 0.93 (s, 9H, t-butyl), 1.28–3.10(m, 7H, H-cyclohexane), 4.61 (br.s., 2H, NH_2_, D_2_O exchangeable), 7.88 (br.s., 1H, NH, D_2_O exchangeable), 8.32(s, 1H, H-pyrimidine); MS, m/z: 276(M + ., 9.20%). Anal. Calcd. For C_14_H_20_N_4_S (276): C, 60.84; H, 7.29; N, 20.27. Found: C, 60.81; H, 7.31; N, 20.25.

#### Ethyl 2-((7-(tert-butyl)-6,7,8,9-tetrahydrobenzo[4,5]thieno[3,2-d]pyrimidin-4-yl)thio)acetate (18)

A mixture of compound **16** (0.01 mol, 2.78 g) and ethyl chloro acetate (0.01 mol, 1.22 mL) in the presence of dry acetone containing anhydrous potassium carbonate (0.01 mol, 1.38 g) was heated under reflux on a water bath for 24 h. Evaporation of the solvent and washing of the formed precipitate with petroleum ether and then filtered off, dried, and recrystallized from ethanol to obtain compound **18**.

Yield (54%); mp = 98–100 °C (ethanol); FT-IR (KBr) (υ cm^−1^): 1561(C = C), 1595(C = N), 1746(C = O), 2944 (CH olefinic); ^1^H-NMR (DMSO-d6) δH (ppm): 0.93 (s, 9H, t-butyl), 1.17–1.21(t, 3H, CH_3_), 1.32–3.28(m, 7H, H-cyclohexane), 4.10–4.16(q, 2H, CH_3_), 8.65(s, 1H, H-pyrimidine); MS, m/z: 364(M^+.^, 26.88%). Anal. Calcd. For C_18_H_24_N_2_O_2_S_2_ (364): C, 59.31; H, 6.64; N, 7.69. Found: C, 59.34; H, 6.61; N, 7.72.

#### 2-amino-6-(tert-butyl)-4,5,6,7-tetrahydrobenzo[*b*]thiophene-3-carboxamide (19)

A mixture of t-butyl cyclohexanone (0.01 mol, 1.54 g), cyano acetamide (0.01 mol, 0.84 g) and Sulphur element (0.01 mol, 0.32 g) in absolute ethanol (20 mL) containing (0.5 mL) of triethyl amine was heated under reflux condition for 5 h. the reaction mixture was poured into crushed ice / HCl with stirring. The formed precipitate was filtered off, dried and recrystallized from ethanol to give **19.**

Yield (66%); mp. = 218–220 °C (ethanol); FT-IR (KBr) (υ cm^−1^): 1650 (C = O), 3322–3497(2 NH_2_); ^1^H-NMR (DMSO-d6) δH (ppm): 0.89 (s, 9H, t-butyl), 1.15–2.47(m, 7H, H-cyclohexane), 7.19(br.s., 2H, NH_2_, D_2_O exchangeable), 7.88(br.s., 2H, NH_2_, D_2_O exchangeable); MS, m/z: 252(M + ., 20.48%). Anal. Calcd. For C_13_H_20_N_2_OS (252): C, 61.87; H, 7.99; N, 11.10. Found: C, 61.84; H, 8.02; N, 10.13.

### Another method for synthesis of 19

To a solution of compound **1** (0.01 mol, 2.34 g) in sulphuric acid 70% (15 mL) was heated in water bath for 24 h. The reaction mixture was poured into crushed ice with continuous stirring. The formed precipitate was filtered off, dried, and recrystallized from ethanol to give **19.**

#### 7-(tert-butyl)-6,7,8,9-tetrahydrobenzo[4,5]thieno[3,2-d]pyrimidin-4(3H)-one (3)

Compound **19** (0.01 mol, 2.52 g) was added to a mixture of Formic acid/ Formamide (10:5 mL). the reaction mixture was allowed to reflux for 6 h and cooled. The precipitate that formed was filterd off, dried, and recrystallized from benzene to give **3**. Yield (60%).

#### 7-(tert-butyl)-2-phenyl-5,6,7,8-tetrahydrobenzo[4,5]thieno[2,3-d]pyrimidin-4(3H)-one (20)

Fusion of compound **19** (0.01 mol, 2.52 g) with benzoyl chloride (10 mL) was heated under reflux on the hot plate for 6 h. The reaction mixture was left to evaporate the solvent, and then (15 mL) of aqueous alcoholic sodium hydroxide (25%) was added and complete refluxing for 5 h. The formed precipitate was washed with petroleum ether 60–80 to afford compound **20**.

Yield (45%); mp. = 114–116 °C (toluene); FT-IR (KBr) (υ cm^−1^): 1580(C=C), 1601(C=N), 1679(C=O), 3059 (CH aromatic) 3169 (NH); ^1^H-NMR (DMSO-d6) δH (ppm): 0.87,0.91 (s, 9H, t-butyl), 1.21–2.28(m, 7H, H-cyclohexane), 7.47–7.96(m, 5H, Ar–H), 12.99 (br.s., 1H, NH, D_2_O exchangeable); MS, m/z: 338(M + ., 21.35%). Anal. Calcd. For C_20_H_22_N_2_OS (338): C, 70.97; H, 6.55; N, 8.28. Found: C, 71.00; H, 6.58; N, 8.25.

#### 7-(tert-butyl)-5,6,7,8-tetrahydrobenzo[4,5]thieno[2,3-d]pyrimidine-2,4(1H,3H)-*dione* (21)

A mixture of compound **19** (0.01 mol, 2.52 g) and ethyl chloro formate (0.01 mol, 1.08 mL) in absolut**e** ethanol (20 mL) containing fused sodium acetate (0.01 mol, 0.82 g) was heated for 8 h. The reaction mixture was poured into crushed ice/ HCl. The formed precipitate was filtered off, dried, and recrystallized from ethanol to afford **21**.

Yield (52%); mp. = 128–130 °C (ethanol); FT-IR (KBr) (υ cm^−1^): 1657(2 C=O), 3207(2 NH); ^1^H-NMR (DMSO-d6) δH (ppm): 0.84, 0.92 (s, 9H, t-butyl), 1.14–2.32 (m, 7H, H-cyclohexane), 11.32 (br.s., 1H, NH, D_2_O exchangeable), 12.42 (br.s., 1H, NH, D_2_O exchangeable); MS, m/z: 278(M + ., 35.86%). Anal. Calcd. For C_14_H_18_N_2_O_2_S (278): C, 60.41; H, 6.52; N, 10.06. Found: C, 60.44; H, 6.49; N, 10.09.

### Determination of total antioxidant capacity (TAC)

The antioxidant activity of each compound was determined according to the phosphomolybdenum method using ascorbic acid as standard. This assay is based on the reduction of Mo (VI) to Mo (V) by the sample analyte and the subsequent formation of a green-colored [phosphate = Mo (V)] complex at acidic pH with maximal absorption at 695 nm. In this method, 0.5 ml of each compound (200 µg /ml) in methanol was combined in dried vials with 5 ml of reagent solution (0.6 M sulfuric acid, 28 mM sodium phosphate, and 4 mM ammonium molybdate). The vials containing the reaction mixture were capped and incubated in a thermal block at 95 °C for 90 min. After the samples had cooled at room temperature, the absorbance was measured at 695 nm against a blank. The blank consisted of all reagents and solvents without the sample, and it was incubated under the same conditions. All experiments were carried out in triplicate. The antioxidant activity of the sample was expressed as the number of ascorbic acid equivalents (AAE)^66–70^.

### Molecular docking

To clarify the mode of action and predict the potency of the tested drugs, molecular docking studies were performed, emphasizing the interactions between ligands and receptors. Chemdraw 20.0 (CambridgeSoft) from Perkin Inc. was used for drawing ligands and tested compounds. To enhance the fitting and docking results, the Wave Function Spartan v 14.0 program^71^ was utilized to optimize the geometries and minimize the global energies of both the ligands and the tested compounds.

In the docking study, the crystal structure of the binding protein. The X-ray crystal structure of the Keap1 (Kelch-like ECH-associated protein 1) protein was retrieved from the Protein Data Bank (PDB: 7C5E). Auto Dock Vina was employed to perform the molecular docking study, focusing on the interaction between ascorbic acid, and the tested compounds **1**, **16, 17** with Keap1 (Kelch-like ECH-associated protein 1). To prepare the protein receptor, necessary steps such as 3D hydrogenation and energy minimization were carried out^72,73^. The visualization process was conducted using the PyMOL program^74^.

### DFT calculations

The Gaussian 09W program^75^ was employed for Density Functional Theory (DFT) calculations. The calculations were performed at the B3LYP level, a hybrid exchange functional that combines Becke’s three-parameter method (local, non-local, and Hartree–Fock) with the Lee–Yang–Parr correlation functional^76,77^.

For the full geometry optimization of the studied compounds, the 6–31 + G(d,p) basis set was employed. This basis set includes ‘d’ polarization functions for heavy atoms and 'p' polarization functions for hydrogen atoms. Additionally, diffuse functions were incorporated for both hydrogen and heavy atoms to improve accuracy in describing the optimized structures and ground state properties.

To assess the chemical reactivity of compounds 1, 16, 17 and reference drugs, several theoretical descriptors based on conceptual Density Functional Theory (DFT) were determined. These descriptors include the Lowest Unoccupied (vacant) Molecular Orbital (LUMO) energy (ELUMO), the Highest Occupied Molecular Orbital (HOMO) energy (EHOMO), the electronegativity (χ), the global softness (S), and the hardness (η). It is important to note that these descriptors were calculated based on the optimized molecular structures. It should be emphasized that the descriptors associated with the frontier molecular orbitals (FMO) were determined using a simplified approach within the framework of the Koopmans approximation^78^.

## Conclusions

Various heterocyclic frameworks, including pyrimidine-thione, pyrimidinone, and pyridine derivatives, were effectively synthesized from the 2-amino thiophene-3-carbonitrile derivative **1** through reactions with diverse nitrogen and carbon nucleophiles. The chemical structures of all the synthesized compounds were verified through elemental and spectral analyses. The ultimate heterocyclic product was notably influenced by the reaction conditions, such as the reaction medium. The investigation of the total antioxidant capacity (TAC) revealed that compound **1** displays a robust antioxidant effect, comparable to that of ascorbic acid. Moreover, compounds **16** and **17** show substantial activity, exceeding the potency of the first compound. In conclusion, the gathered data indicate the capability of compound **1** to produce novel heterocyclic frameworks with strong antioxidant properties, suggesting potential applications in the treatment of diseases associated with oxidative stress. Furthermore, future research should include in vivo antioxidant experiments and additional in *vitro* antioxidant assays, such as NO and O2·–, to validate the antioxidant potential of these novel heterocyclic frameworks. In silico studies, involving molecular docking and DFT calculations were conducted for the most antioxidant-active compounds, namely **1**, **16**, and **17**, and were compared with ascorbic acid as a reference antioxidant.

## Supplementary Information

Supplementary Information.

## Data Availability

The data that supports the findings of this study are available in the supplementary material of this article.
