# Supplementary material for: Elaboration of newly synthesized tetrahydrobenzo[b]thiophene derivatives and exploring their antioxidant evaluation, molecular docking, and DFT studies
Source: Sci Rep. 2024 Nov 9;14:27339. doi: 10.1038/s41598-024-74275-x (PMC11550804; doi:10.1038/s41598-024-74275-x)
Supplement: Supplementary file 1 — Supplementary Information. [file 41598_2024_74275_MOESM1_ESM.docx]

**
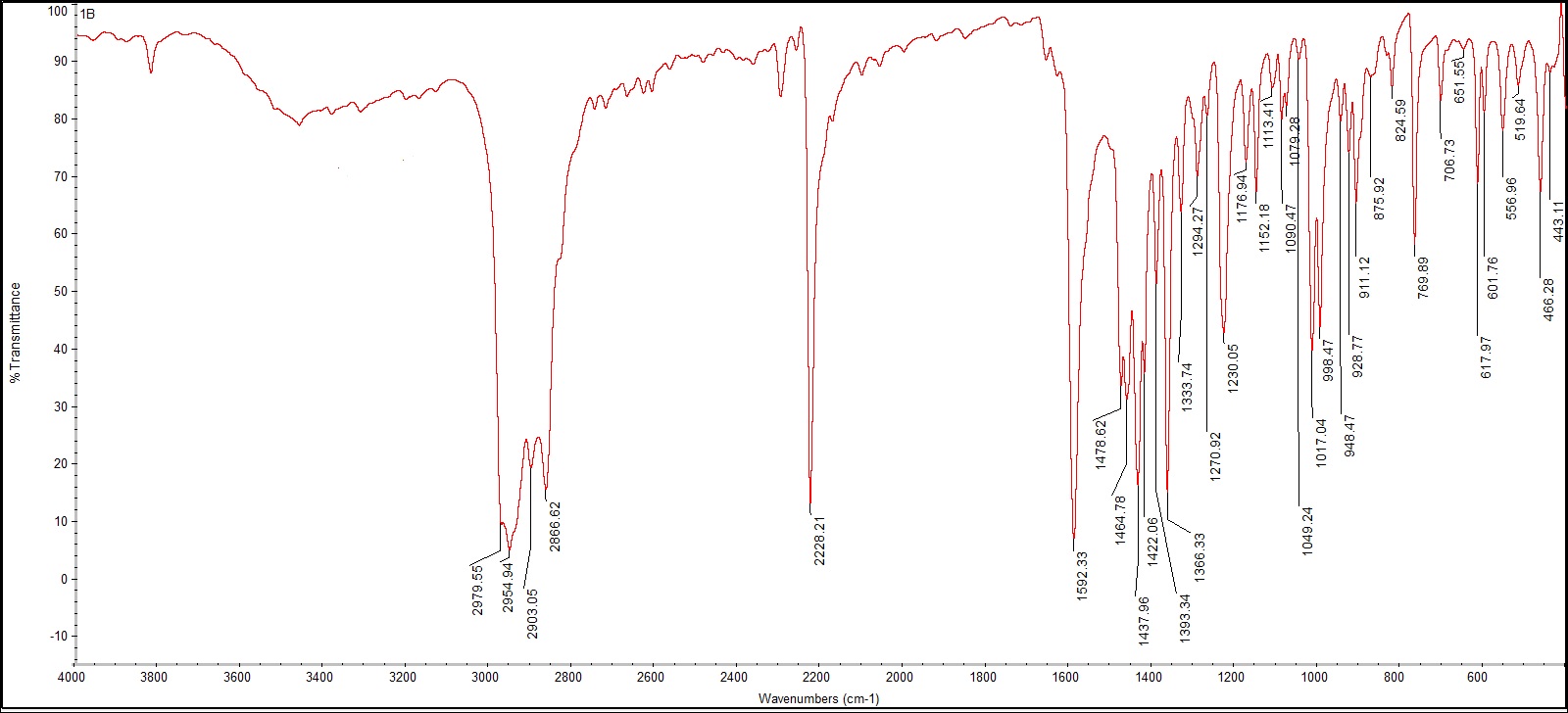
**

**Figure S1**: IR spectrum of compound **1’**

**
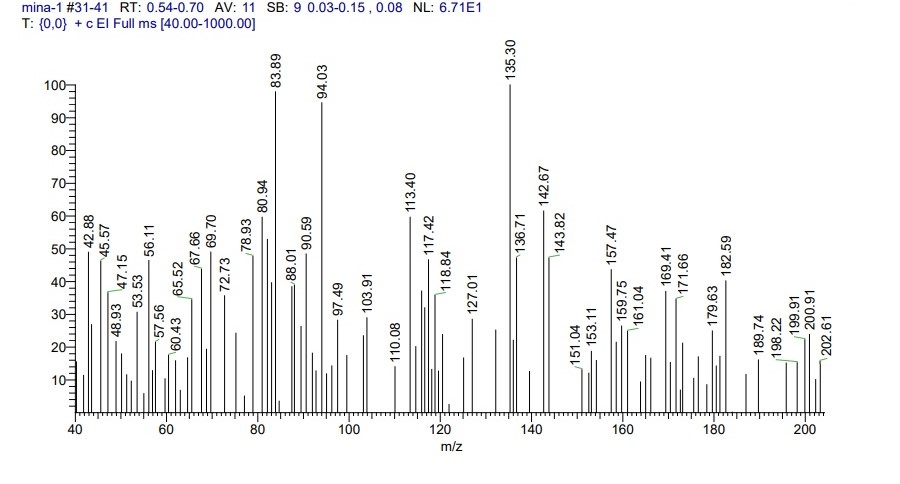
**

**Figure S2**: Mass spectrum of compound **1’**

**
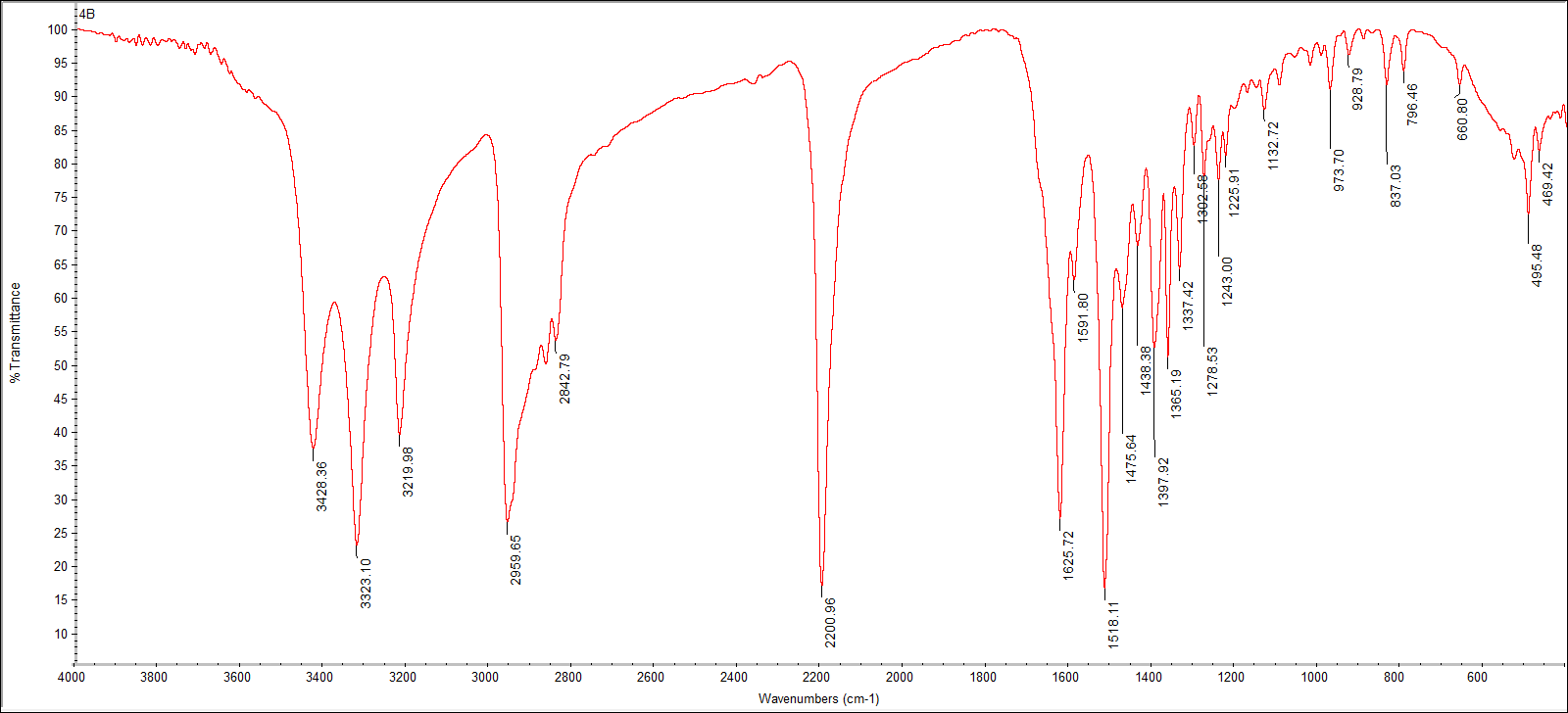
**

**Figure S3**:IR spectrum of compound **2**

**
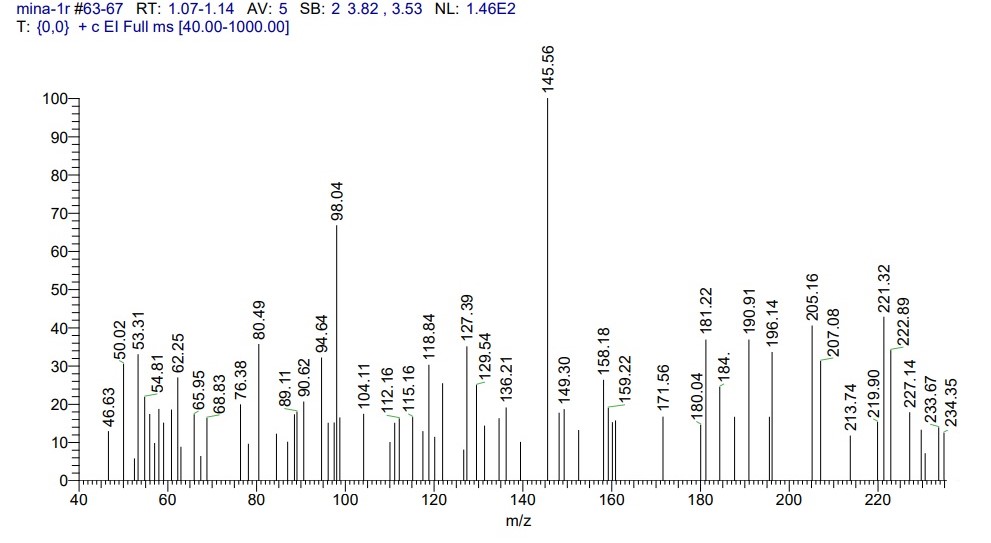
**

**Figure S4**: Mass spectrum of compound **2**

**
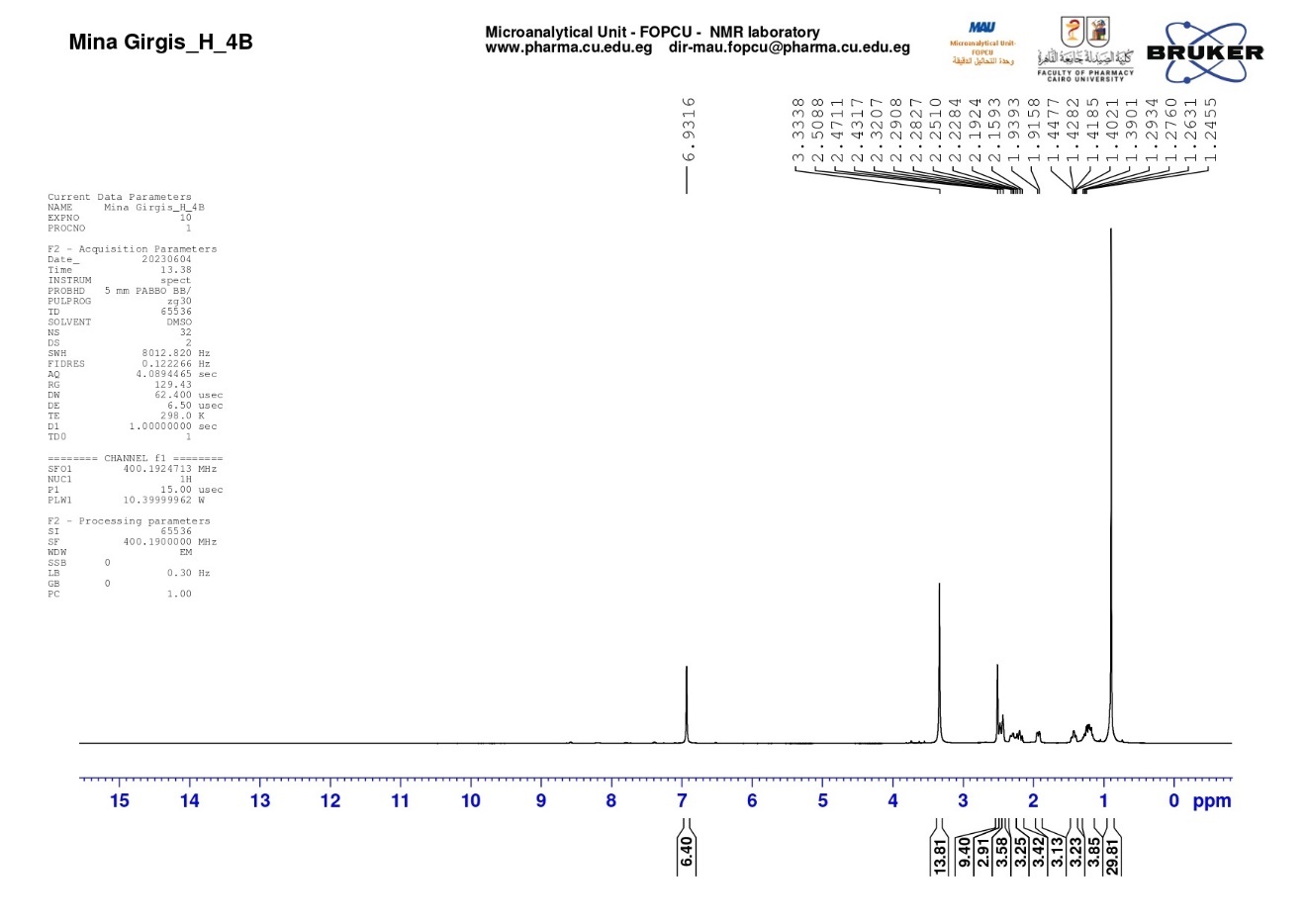
**

**Figure S5**: ^1^H-NMR (DMSO-*d*6) spectrum of compound **2**

**
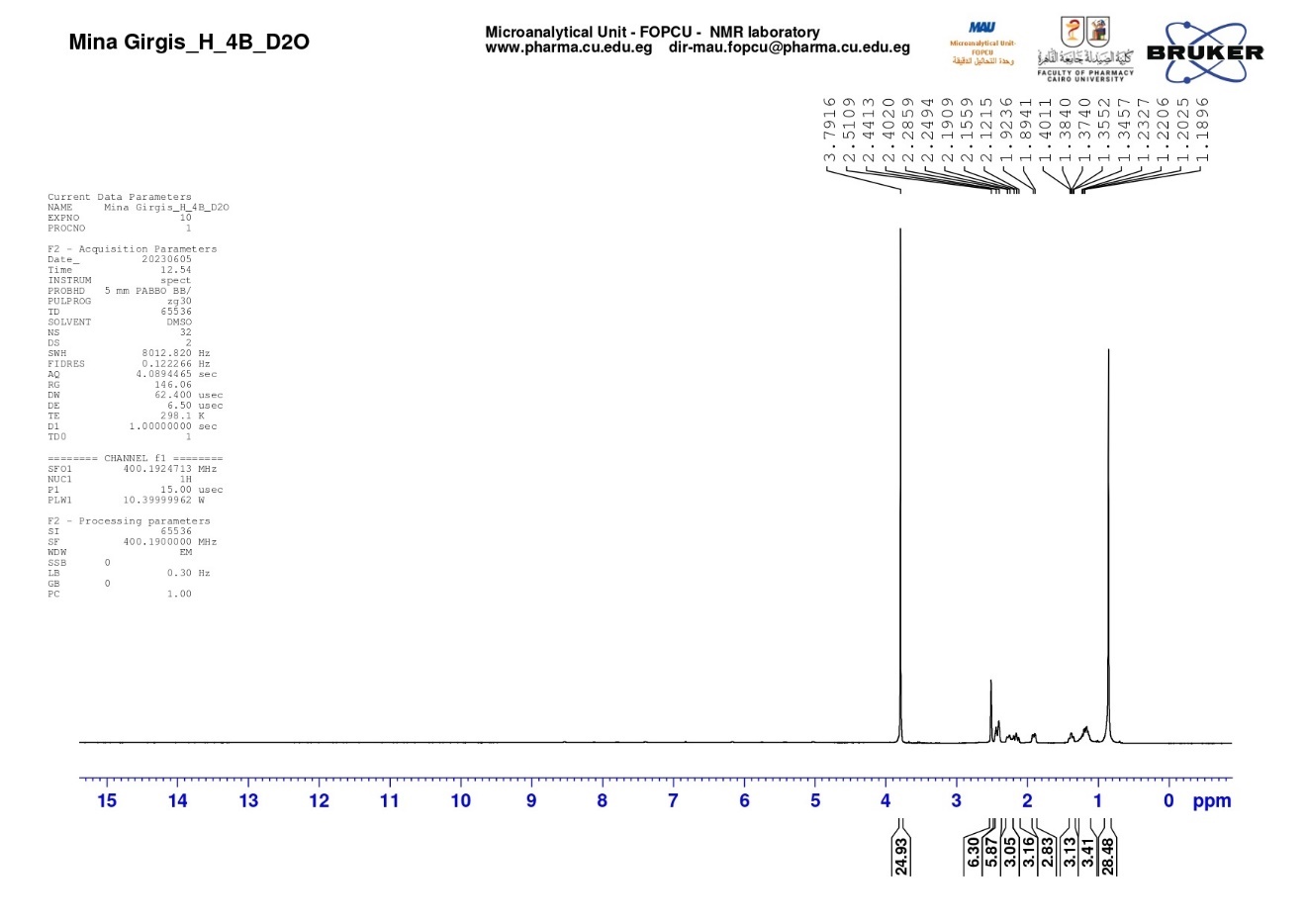
**

**Figure S6**: ^1^H-NMR (DMSO-*d*6 +D_2_O) spectrum of compound **2**

**
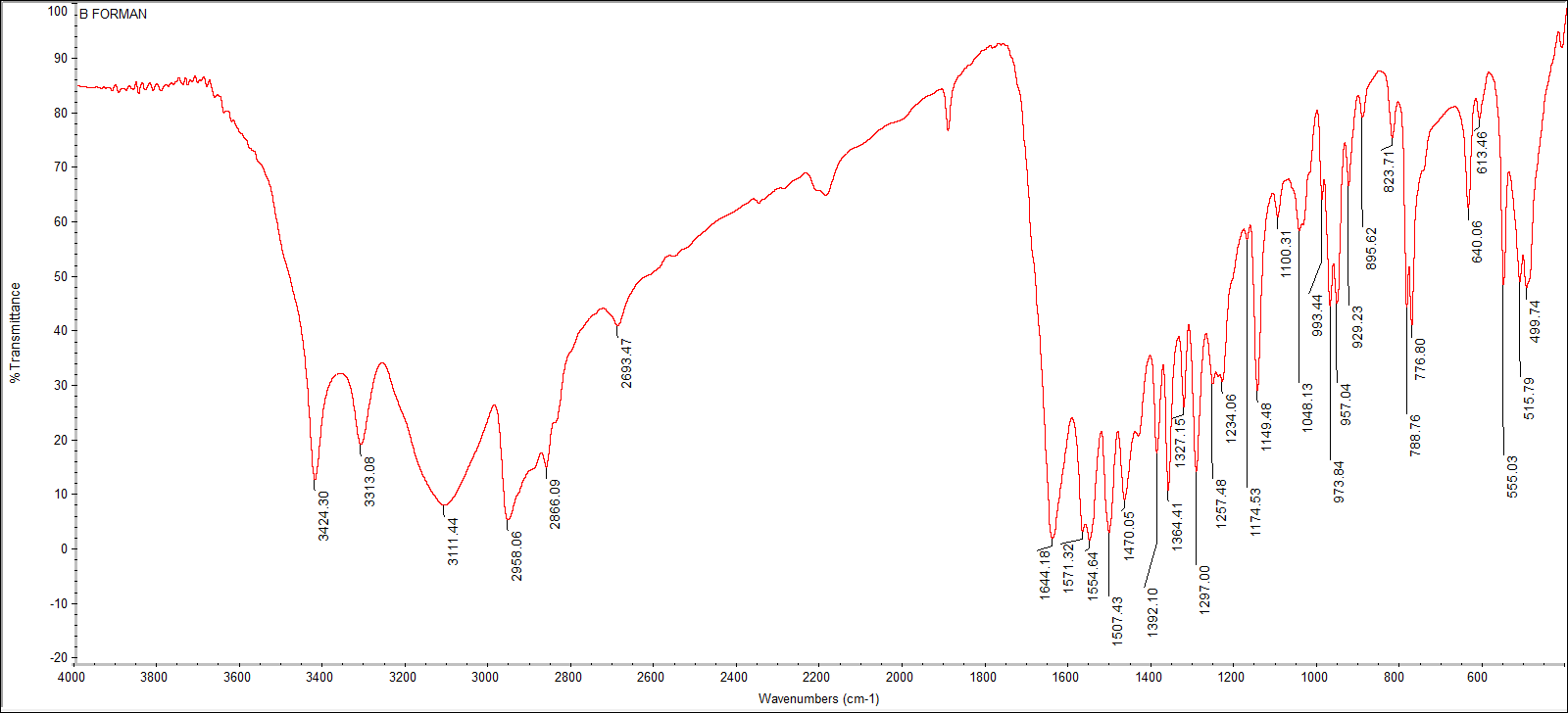
**

**Figure S7**: IR spectrum of compound **2**

**
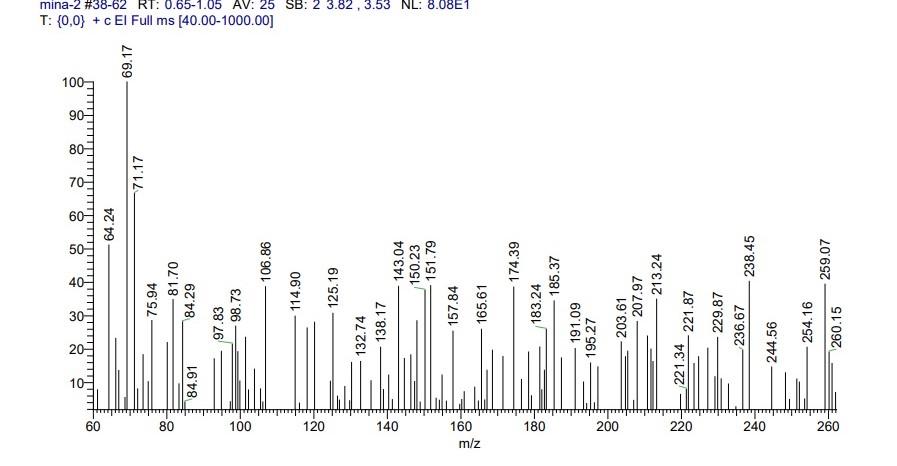
**

**Figure S8**: Mass spectrum of compound **2**

**
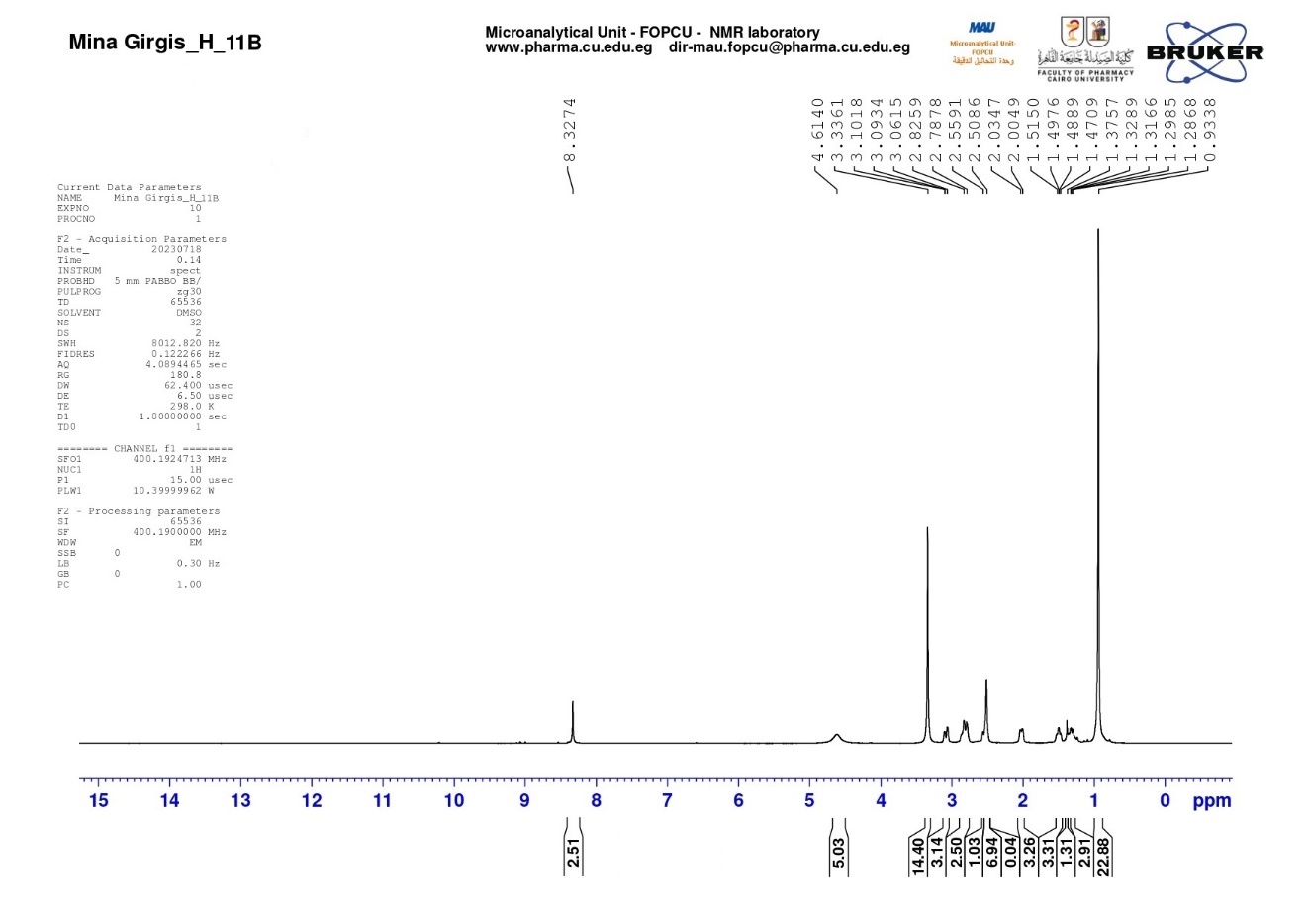
**

**Figure S9**: ^1^H-NMR (DMSO-*d*6) spectrum of compound **2**

**
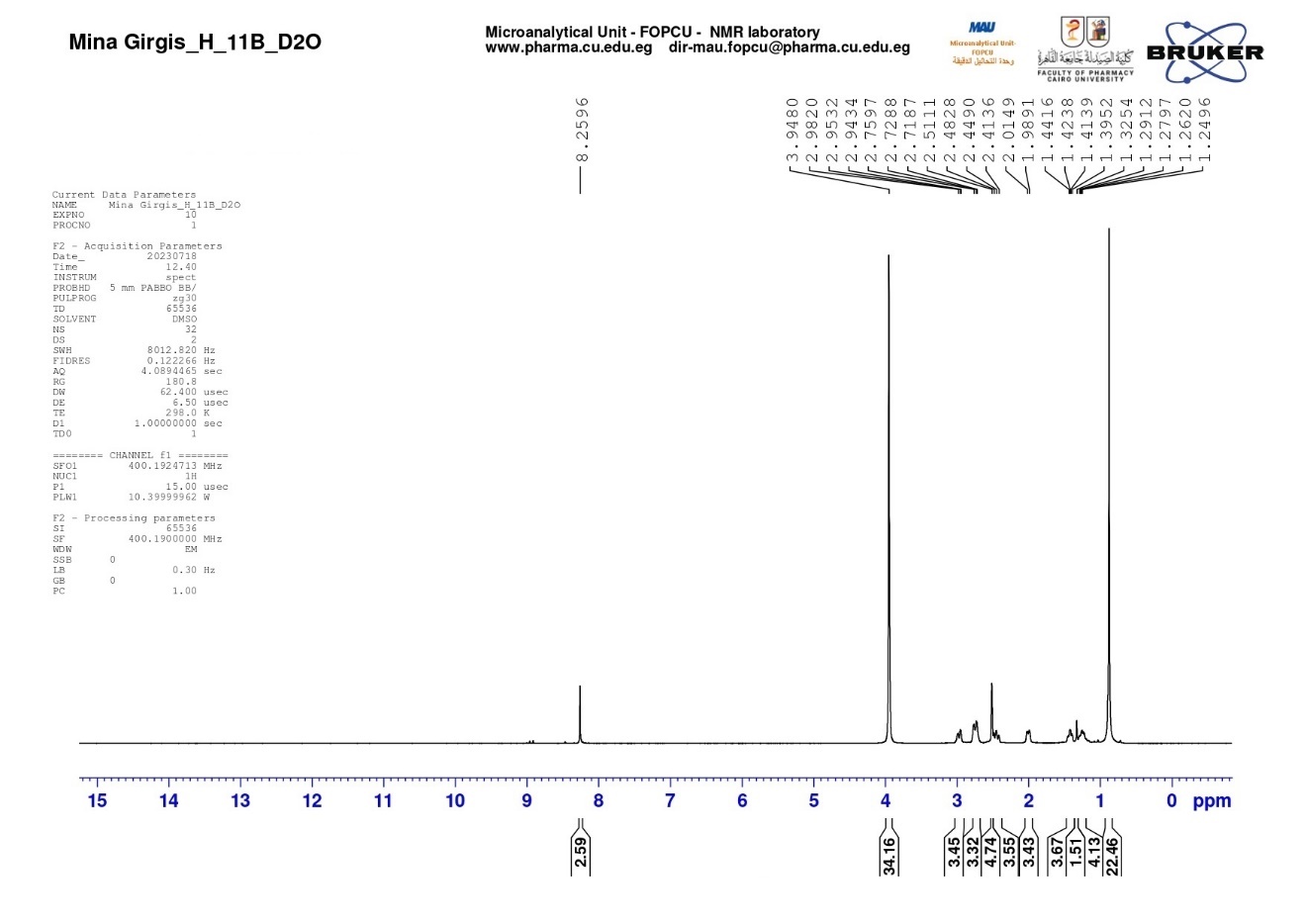
**

**Figure S10**: ^1^H-NMR (DMSO-*d*6 +D_2_O) spectrum of compound **2**

**
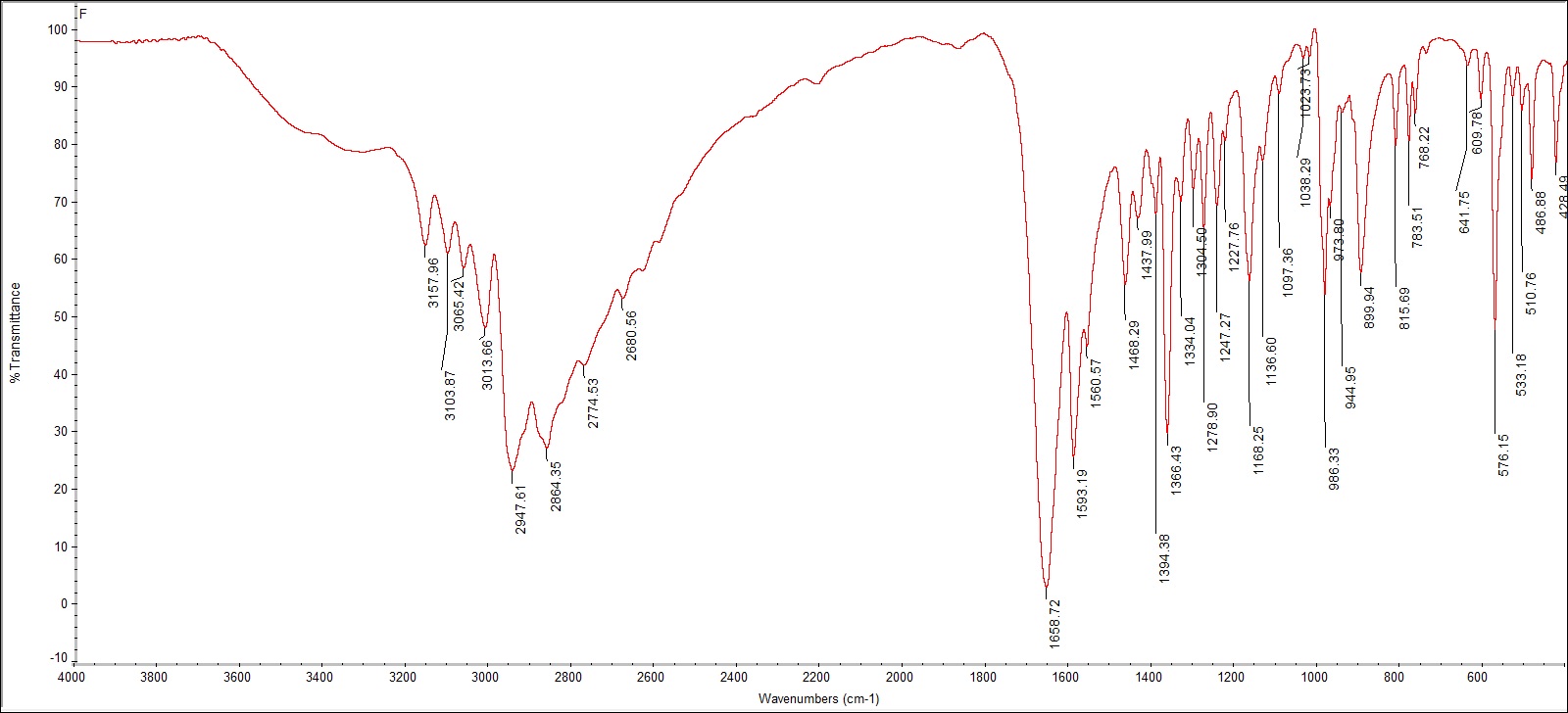
**

**Figure S11**: IR spectrum of compound **3**

**
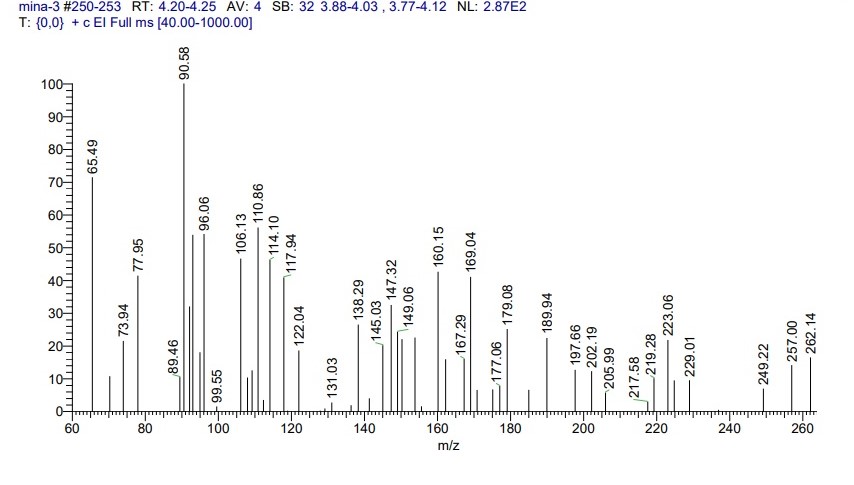
**

**Figure S12**: Mass spectrum of compound **3**

**
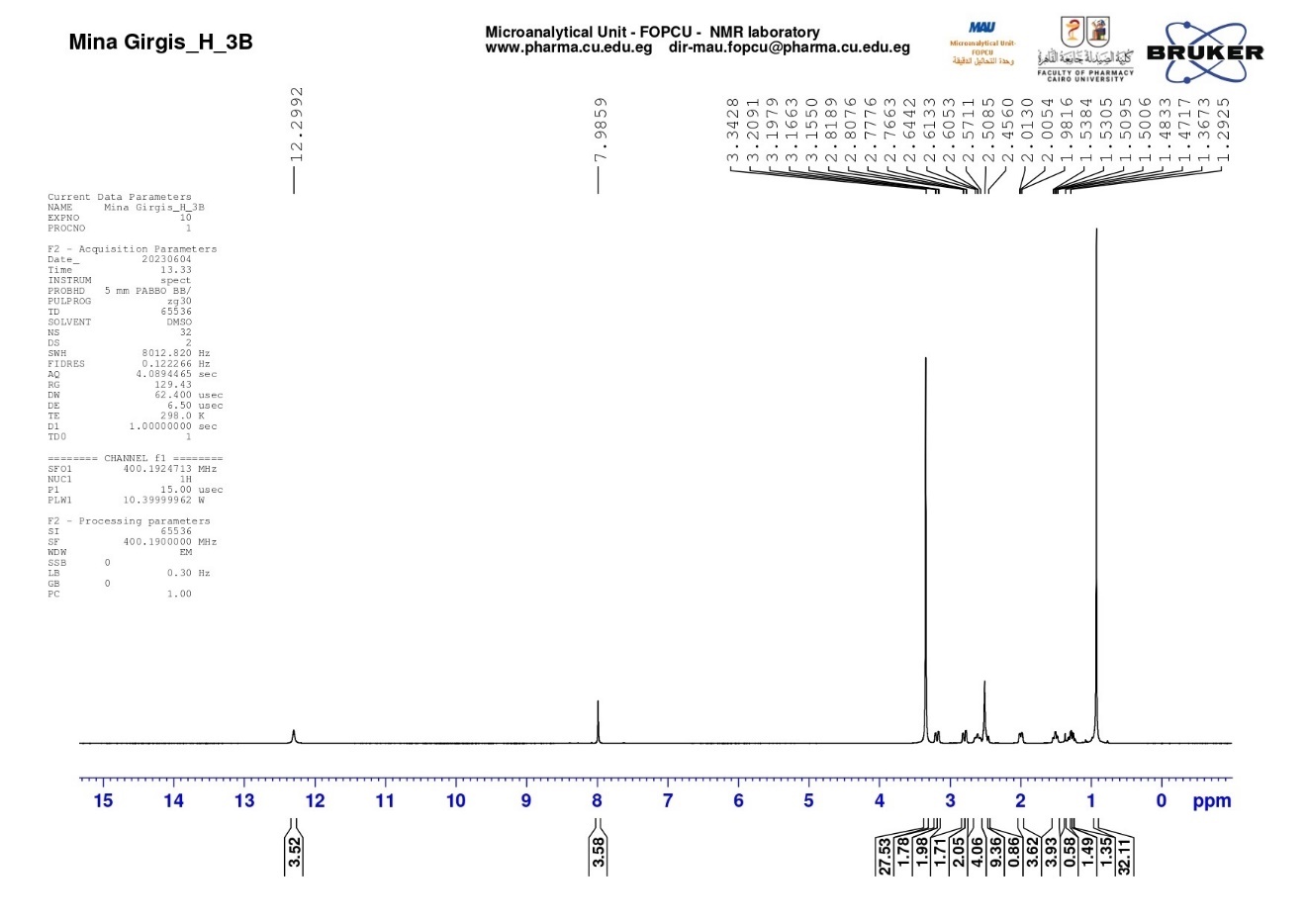
**

**Figure S13**: ^1^H-NMR (DMSO-*d*6) spectrum of compound **3**

**
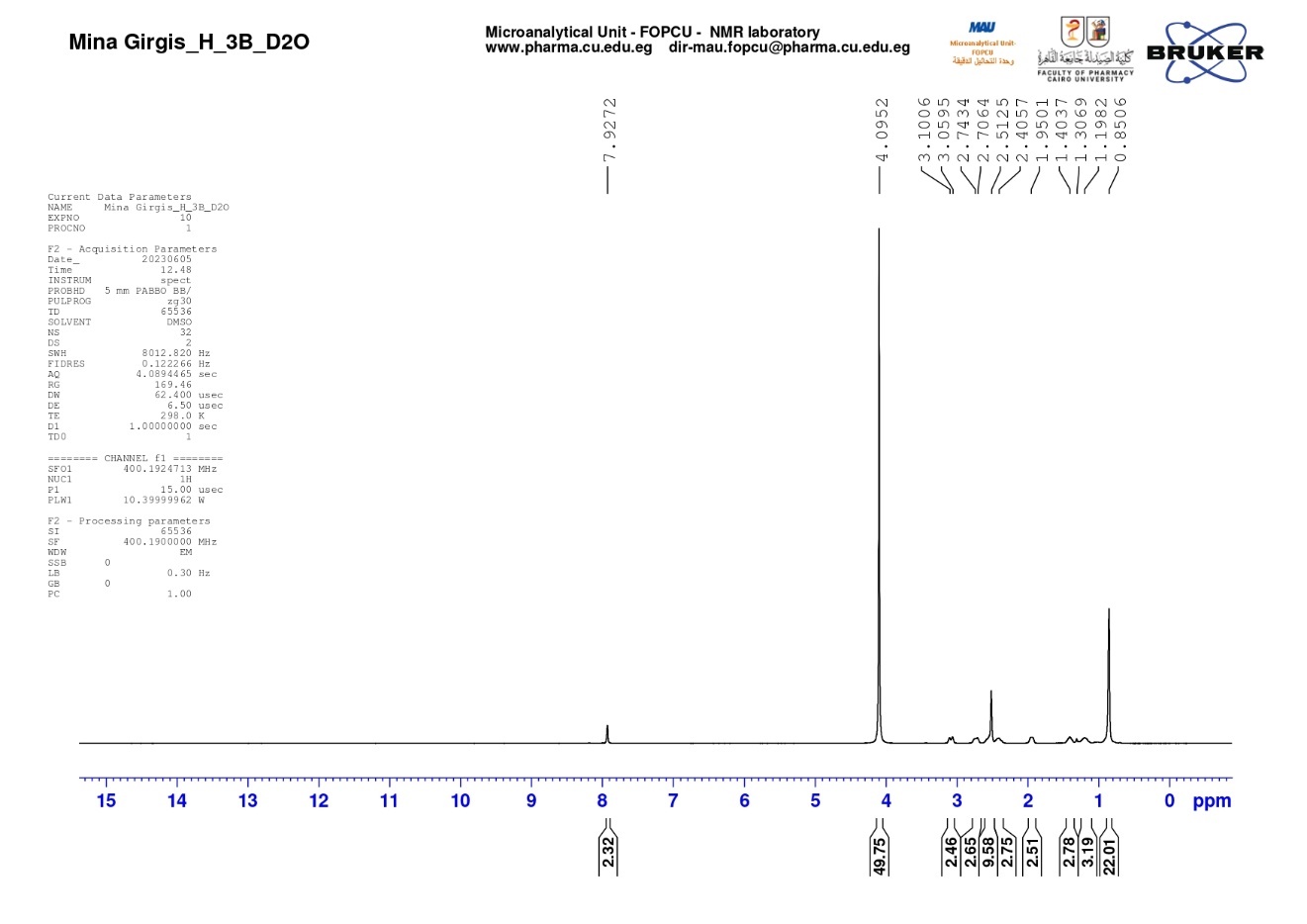
**

**Figure S14**: ^1^H-NMR (DMSO-*d*6 +D_2_O) spectrum of compound **3**

**
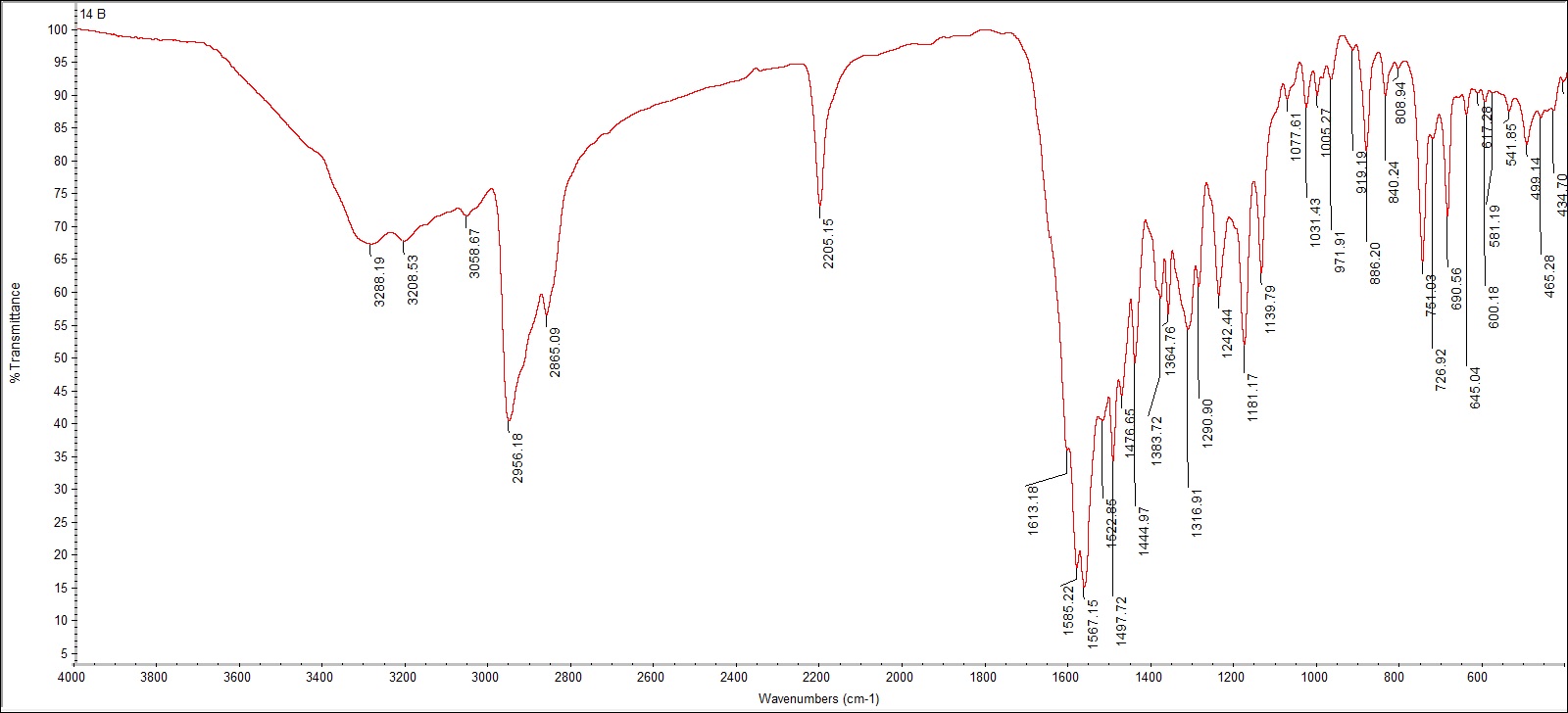
**

**Figure S15**: IR spectrum of compound **4**

**
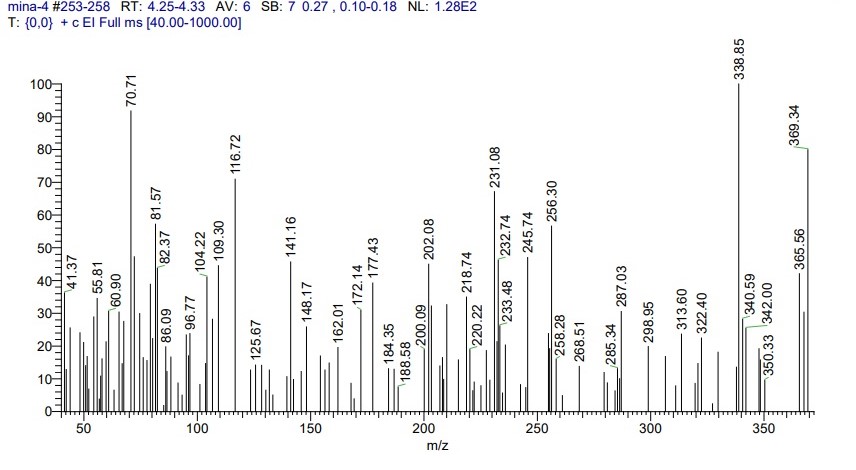
**

**Figure S16**: Mass spectrum of compound **4**

**
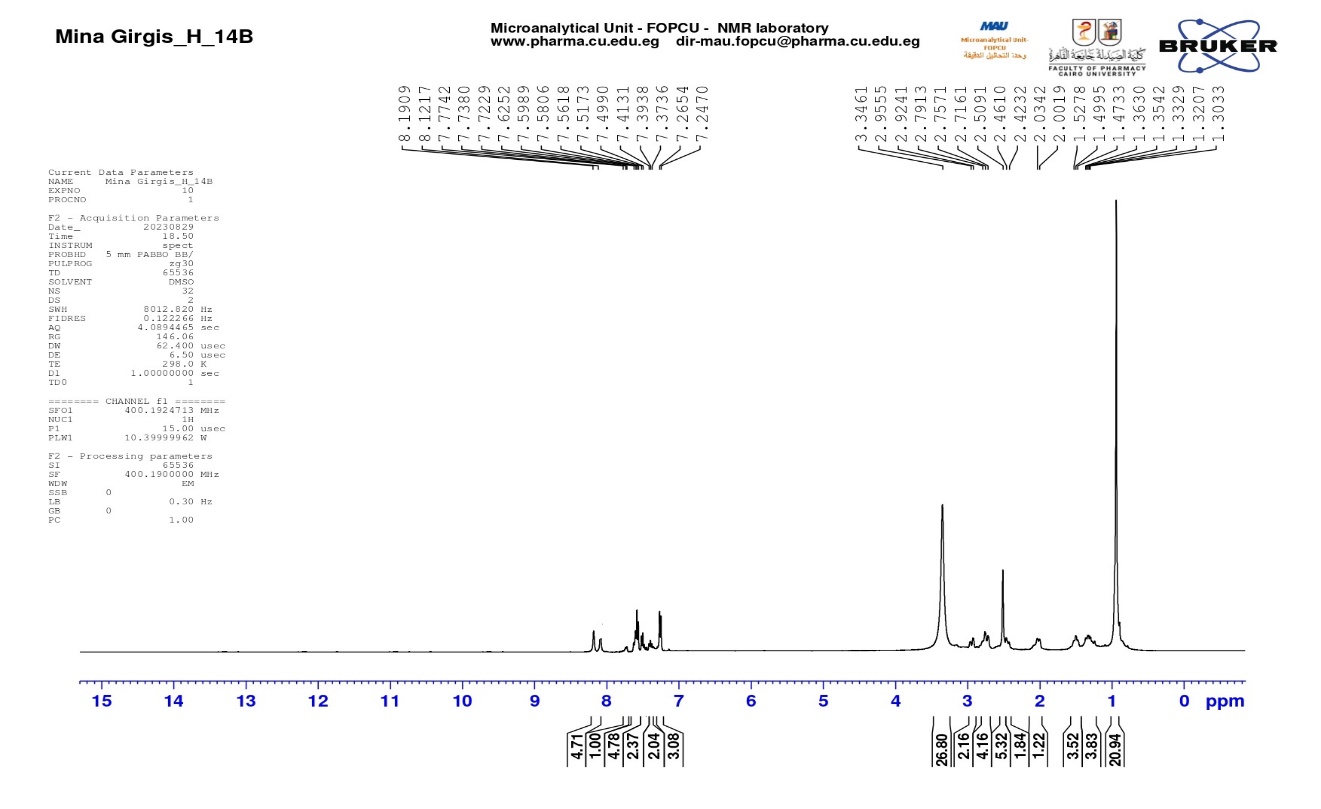
**

**Figure S17**: ^1^H-NMR (DMSO-*d*6) spectrum of compound **4**

**
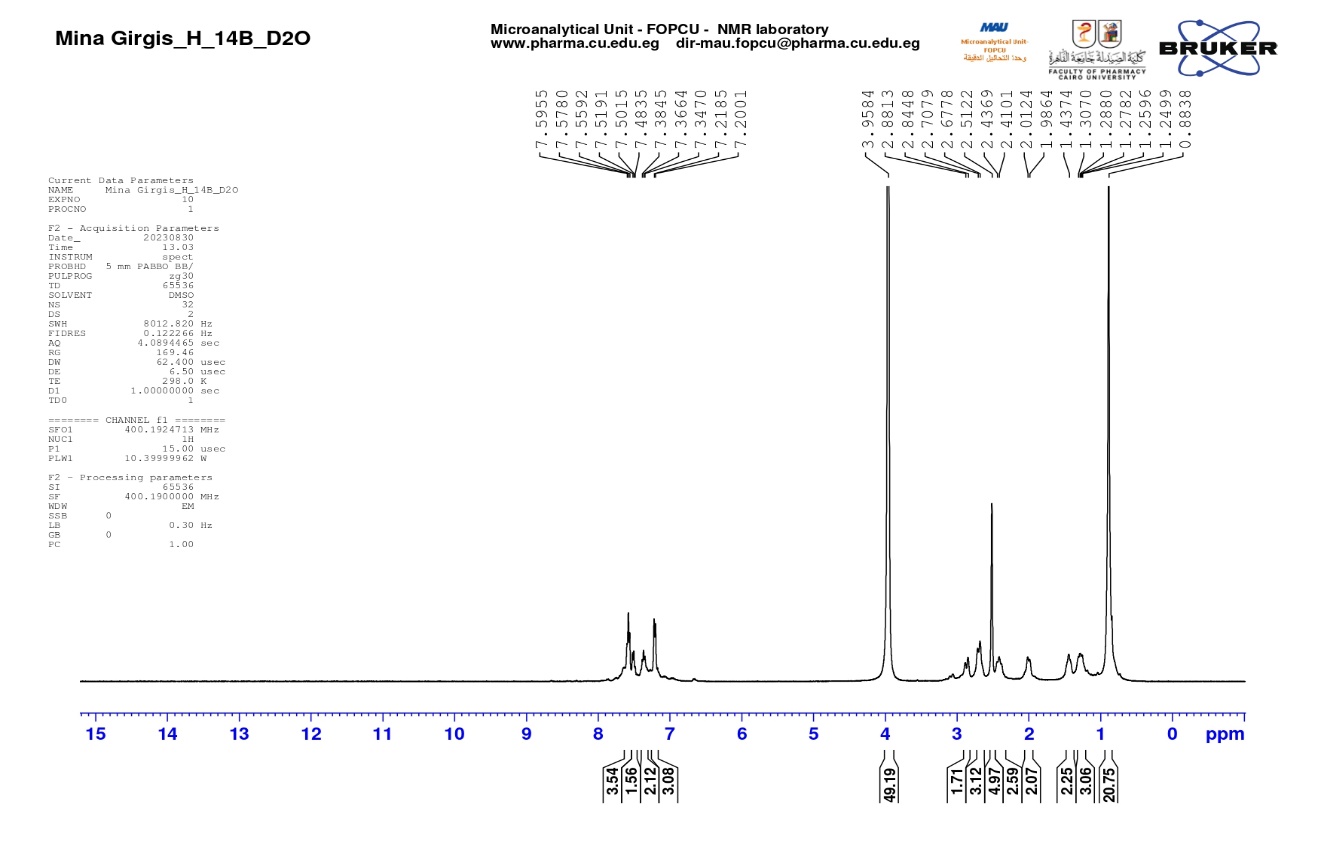
**

**Figure S18**: ^1^H-NMR (DMSO-*d*6+ D_2_O) spectrum of compound **4**

**
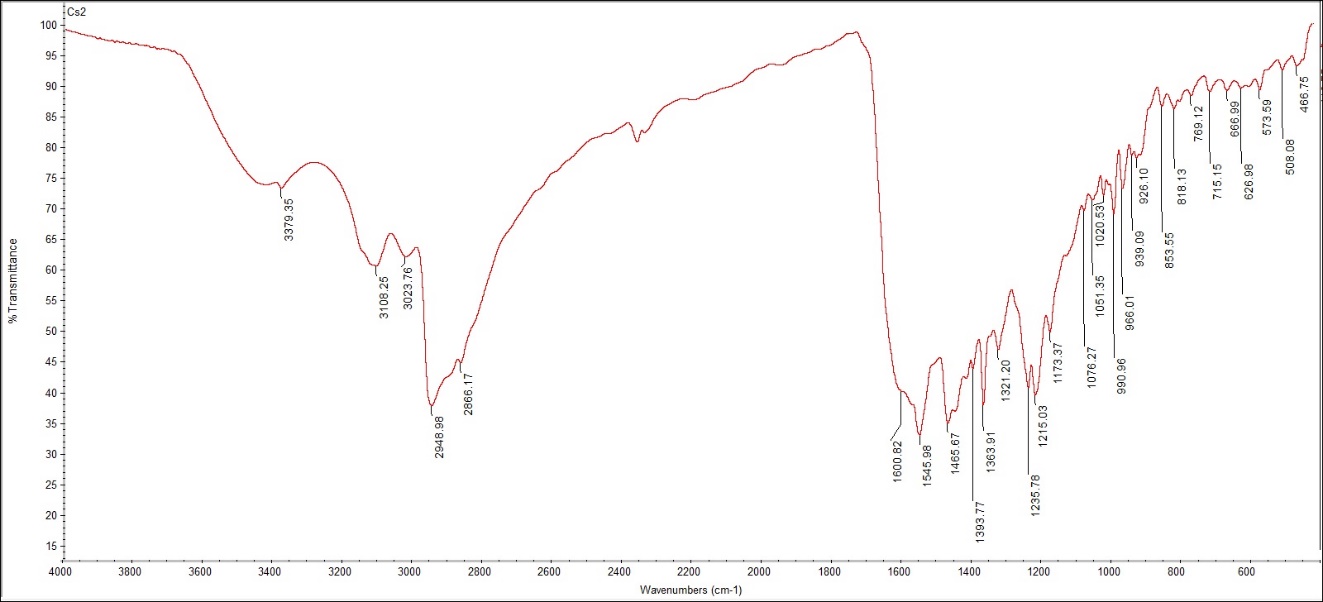
**

**Figure S19**: IR spectrum of compound **5**

**
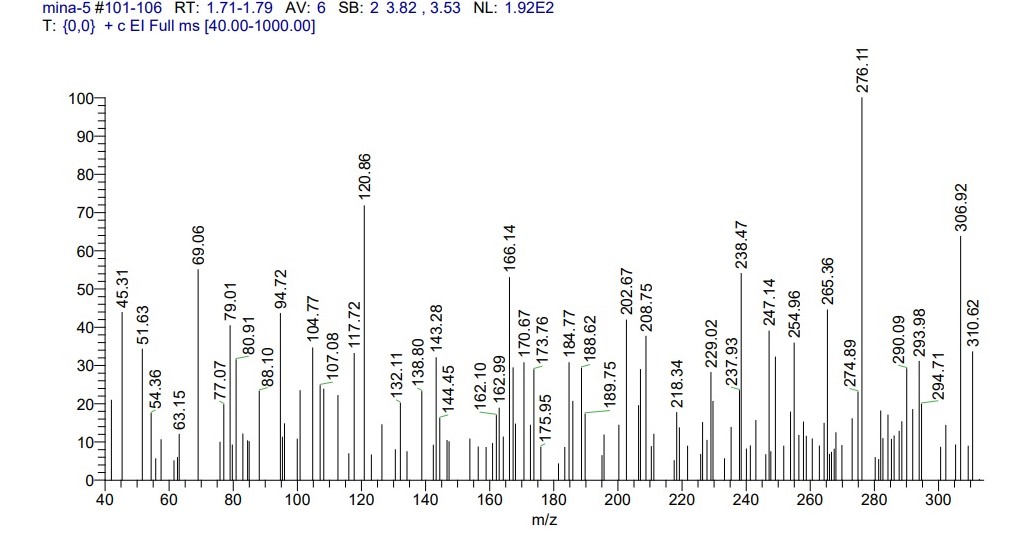
**

**Figure S20**: Mass spectrum of compound **5**

**
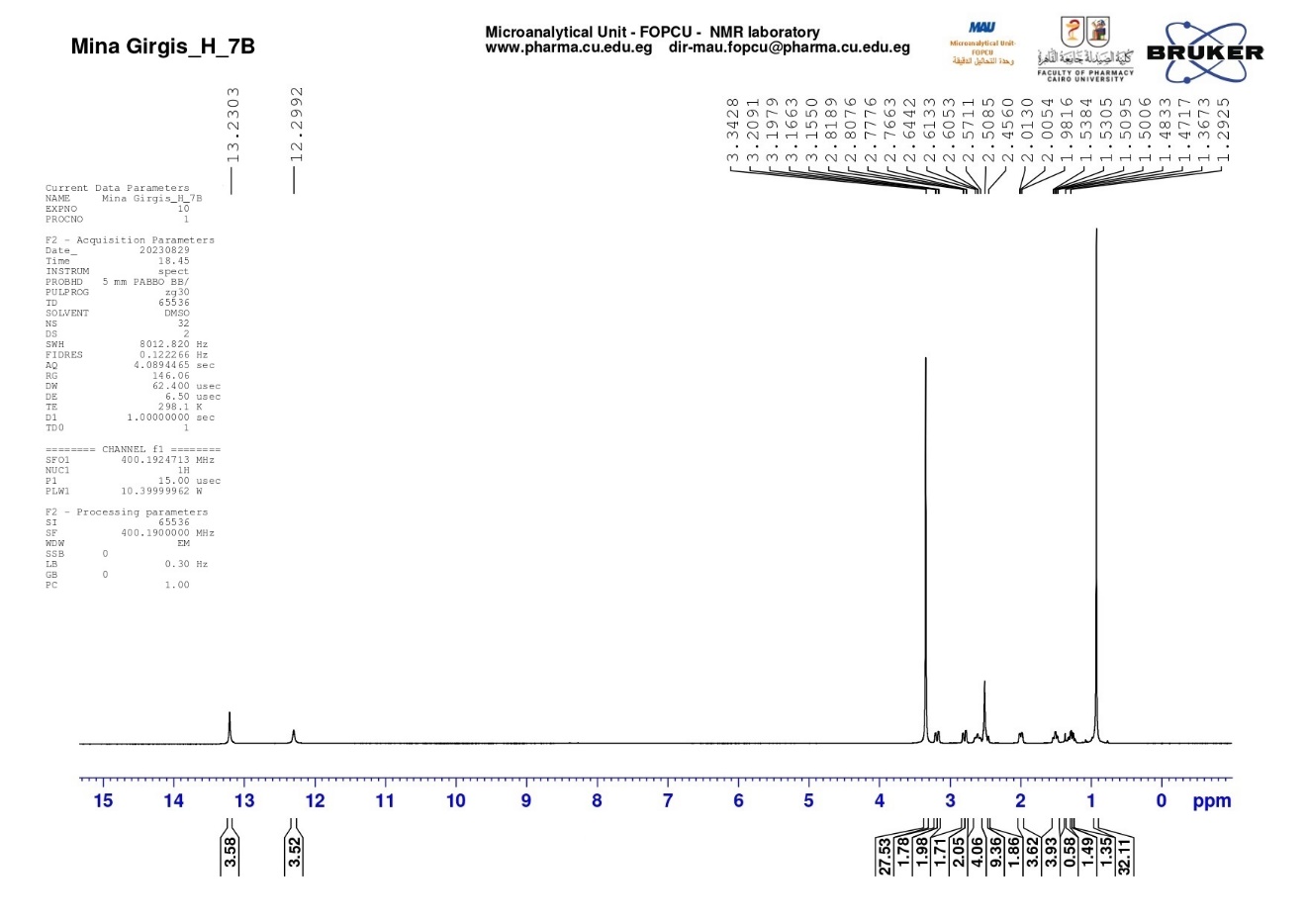
**

**Figure S21**: ^1^H-NMR (DMSO-*d*6) spectrum of compound **5**

**
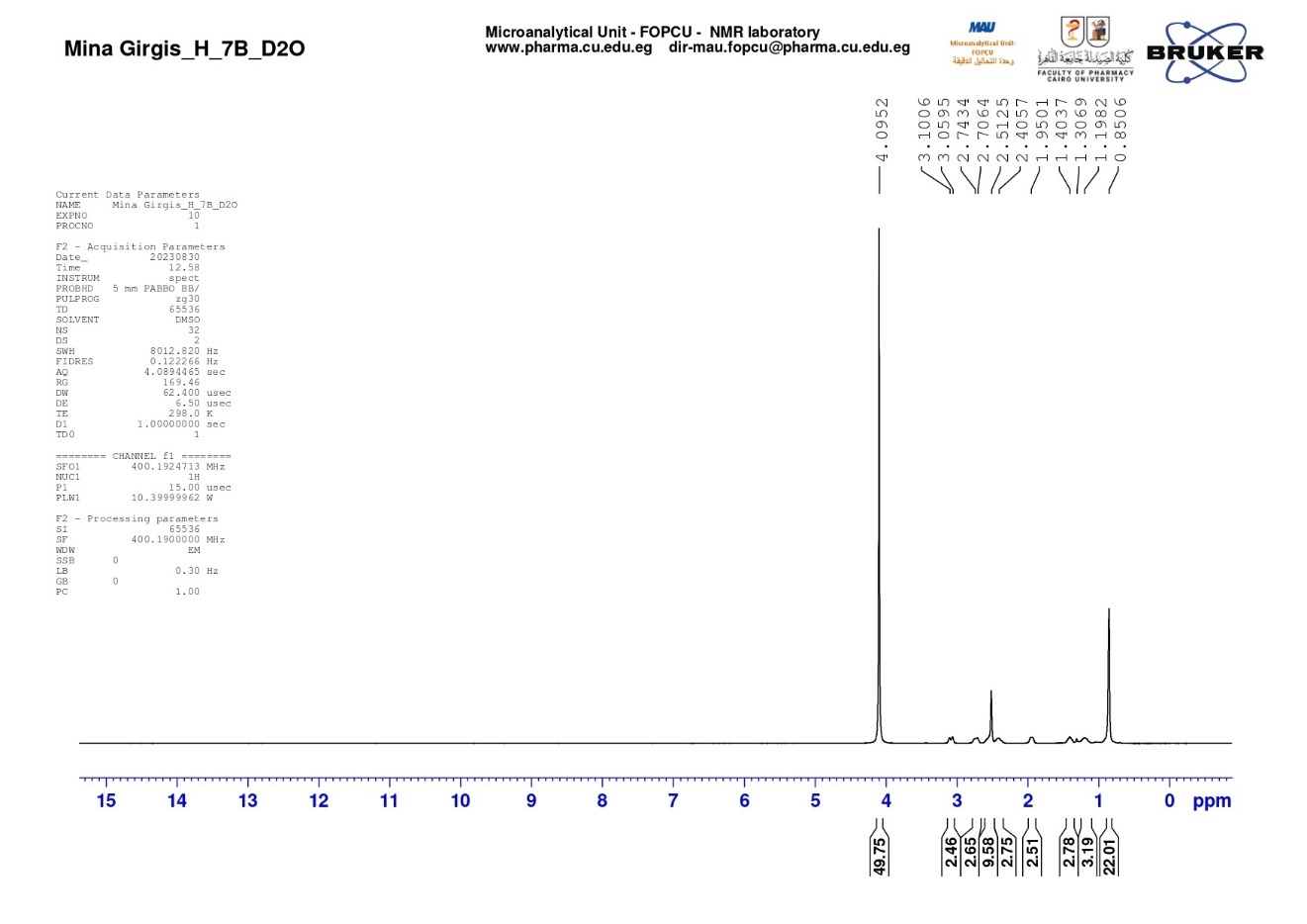
**

**Figure S22**: ^1^H-NMR (DMSO-*d*6+ D_2_O) spectrum of compound **5**

**
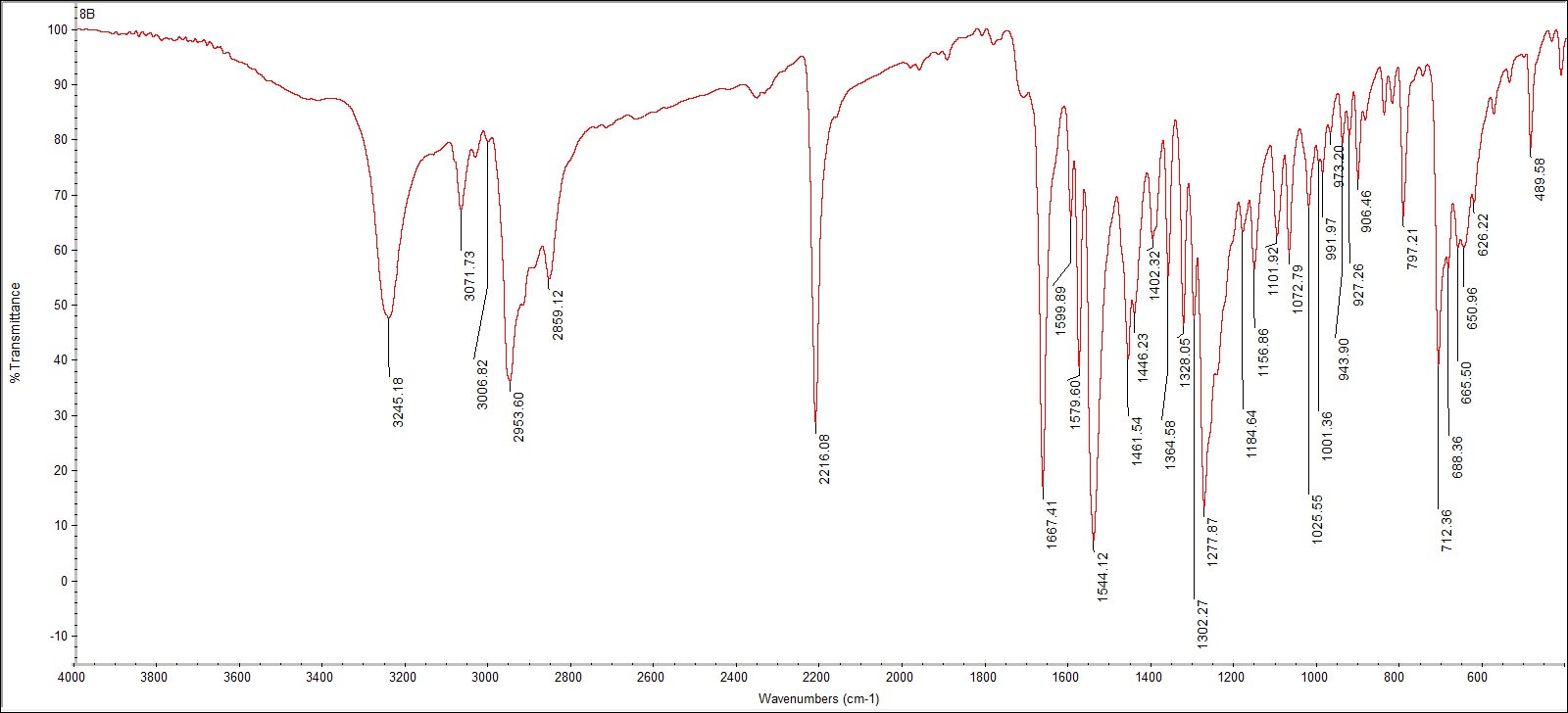
**

**Figure S23**: IR spectrum of compound **6**

**
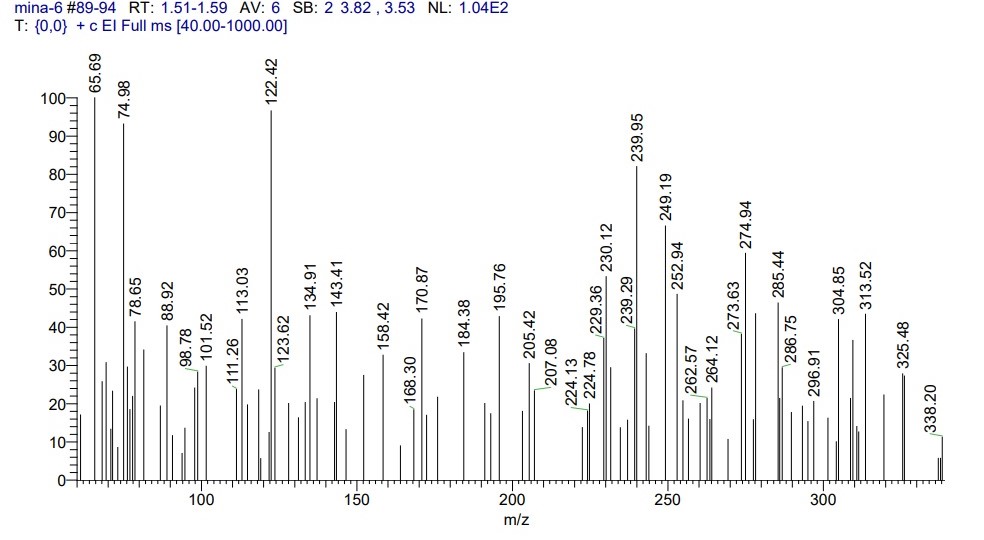
**

**Figure S24**: Mass spectrum of compound **6**

**
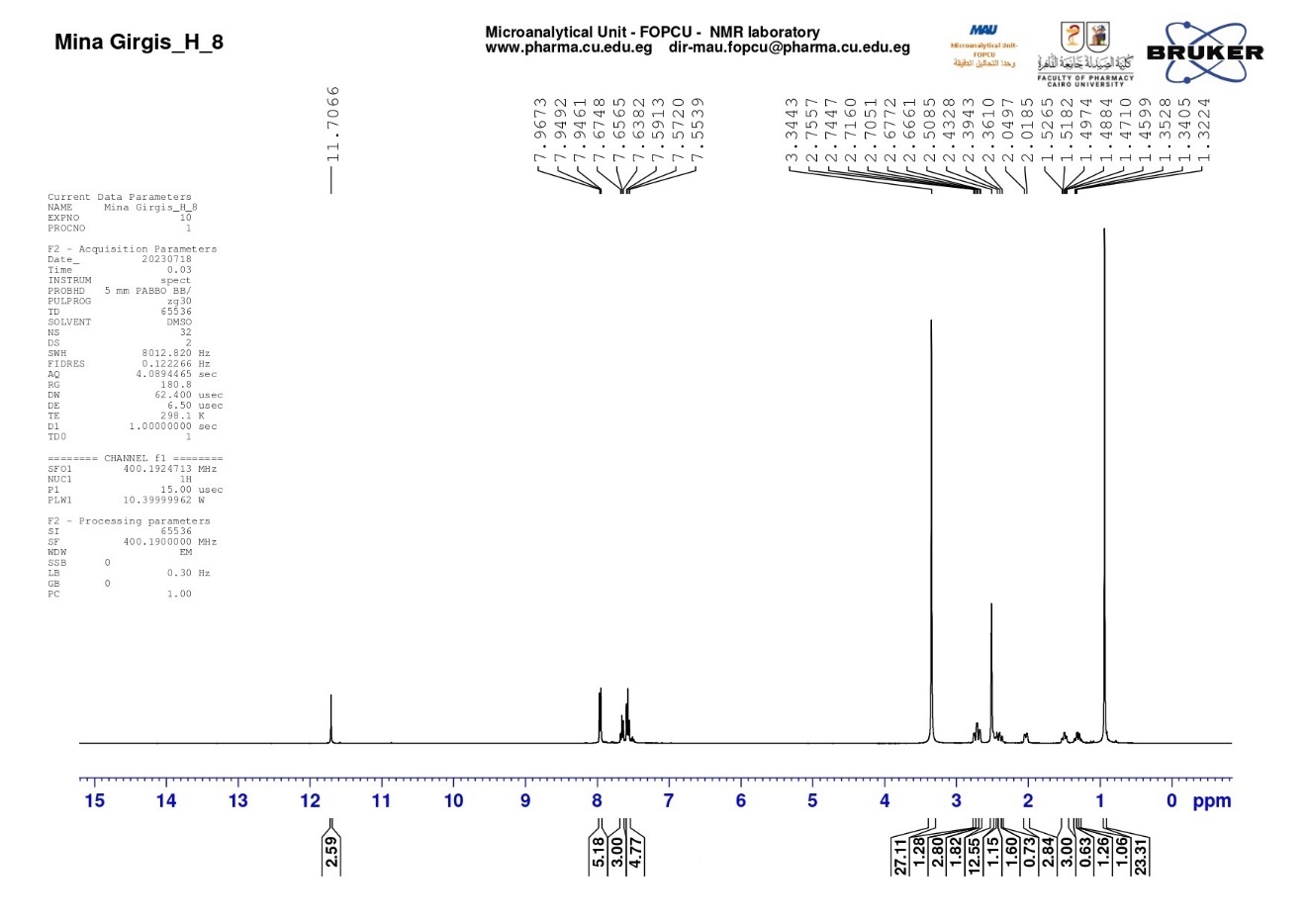
**

**Figure S25**: ^1^H-NMR (DMSO-*d*6) spectrum of compound **6**

**
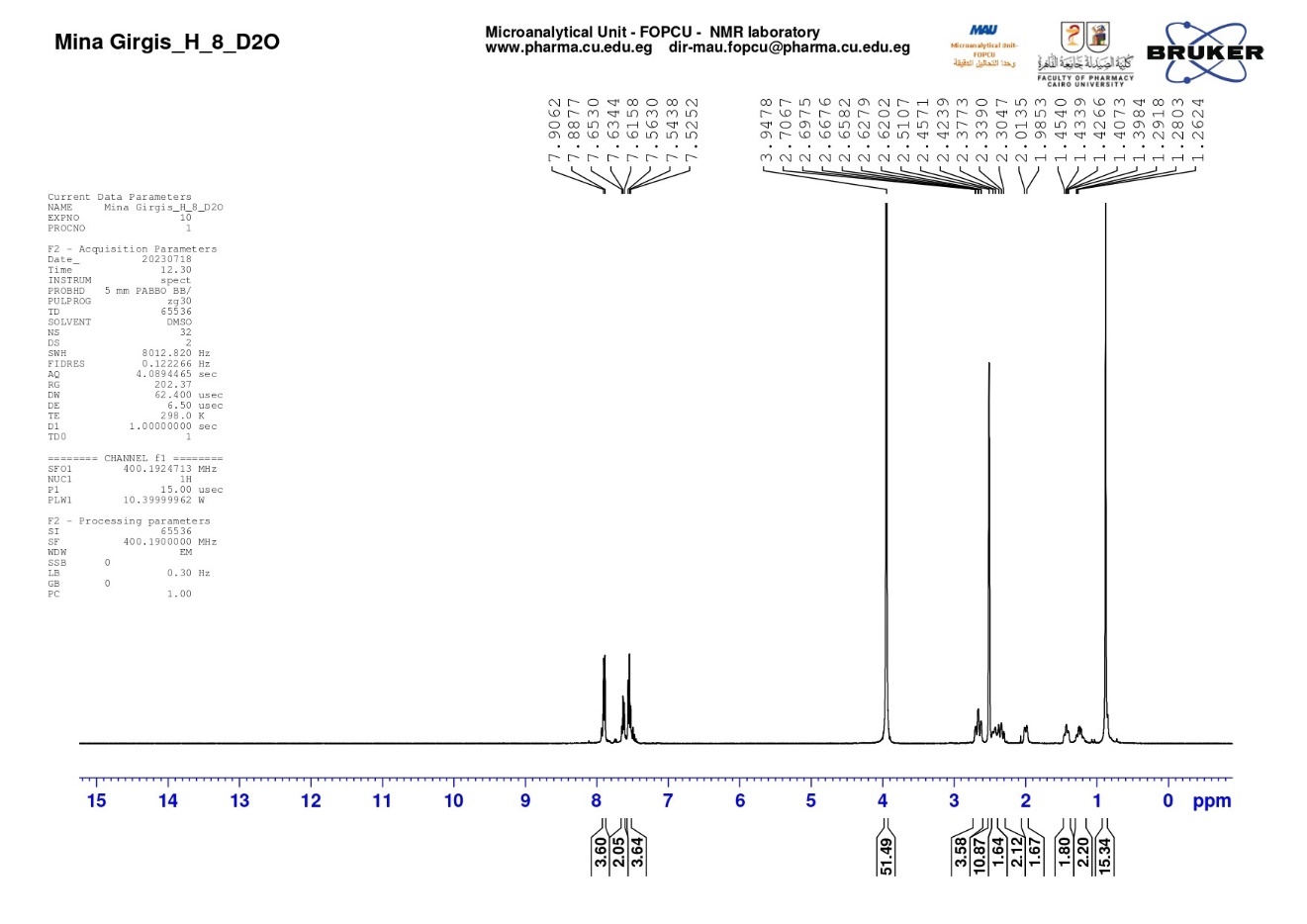
**

**Figure S26**: ^1^H-NMR (DMSO-*d*6+ D_2_O) spectrum of compound **6**

**
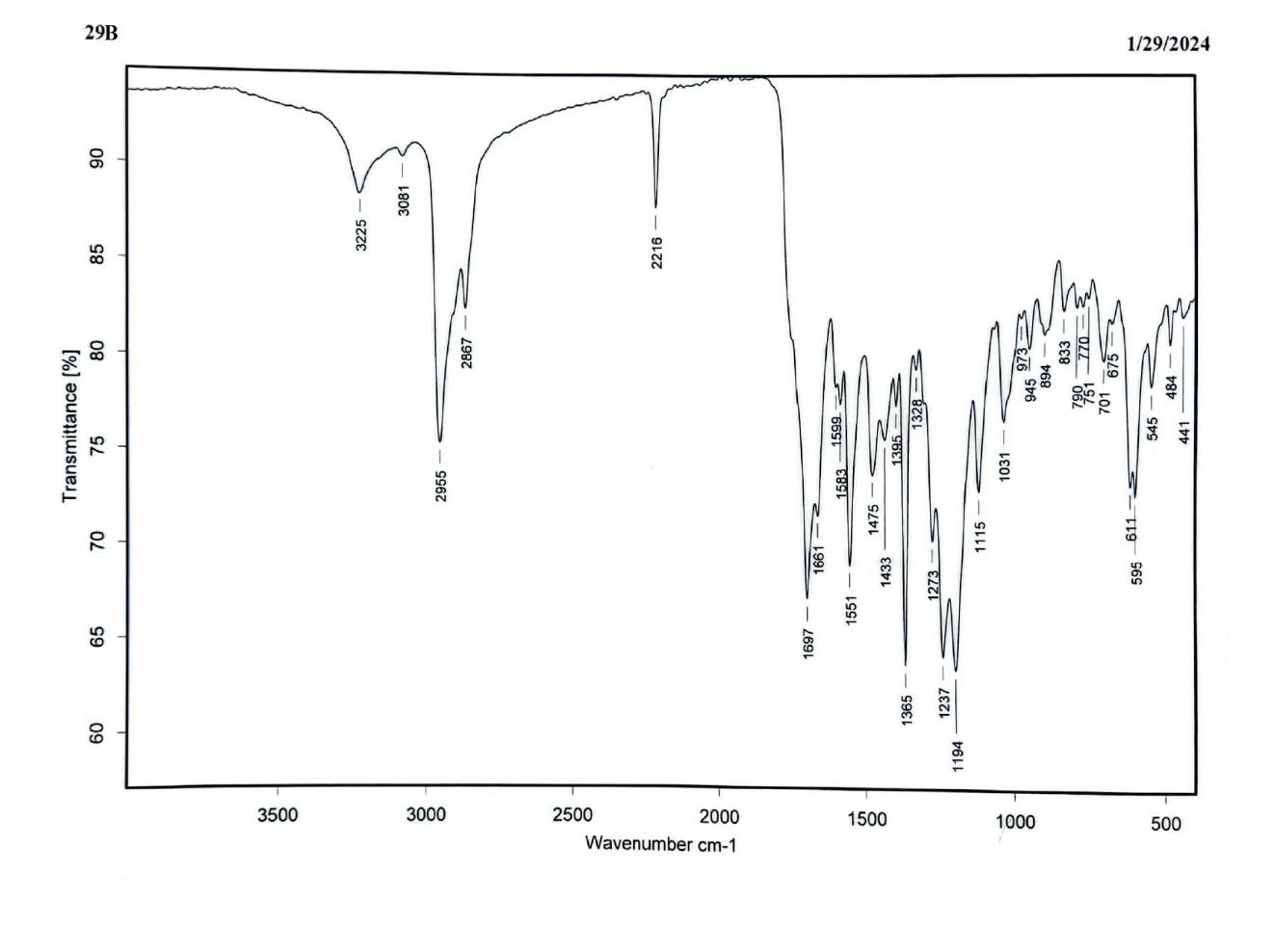
**

**Figure S27**: IR spectrum of compound **7**

**
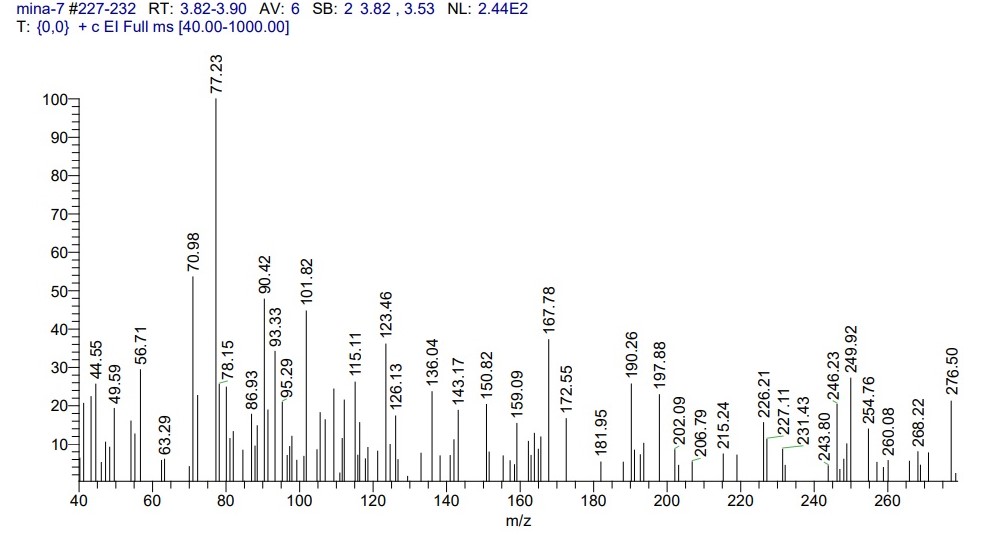
**

**Figure S28**: Mass spectrum of compound **7**

**
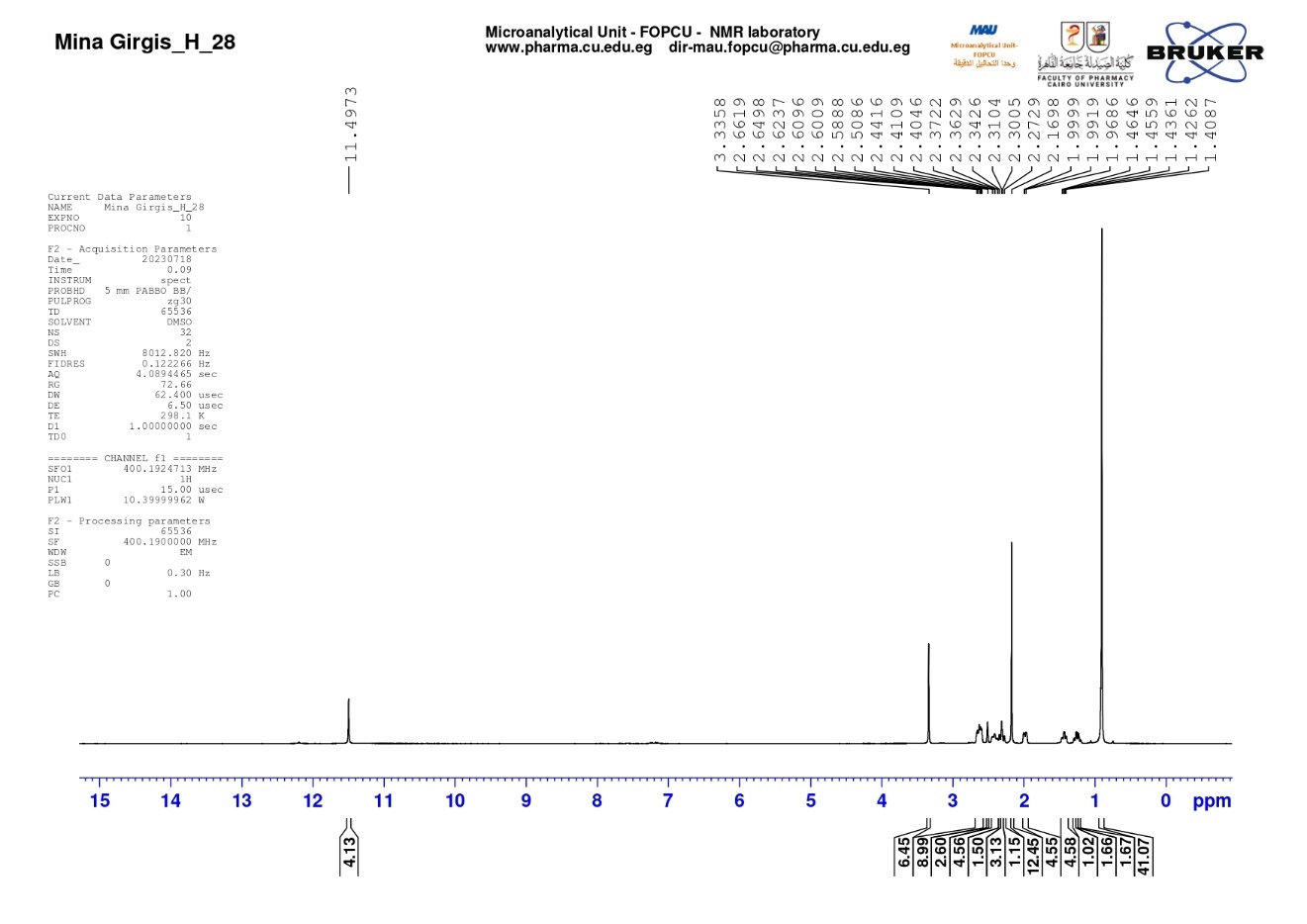
**

**Figure S29**: ^1^H-NMR (DMSO-*d*6) spectrum of compound **7**

**
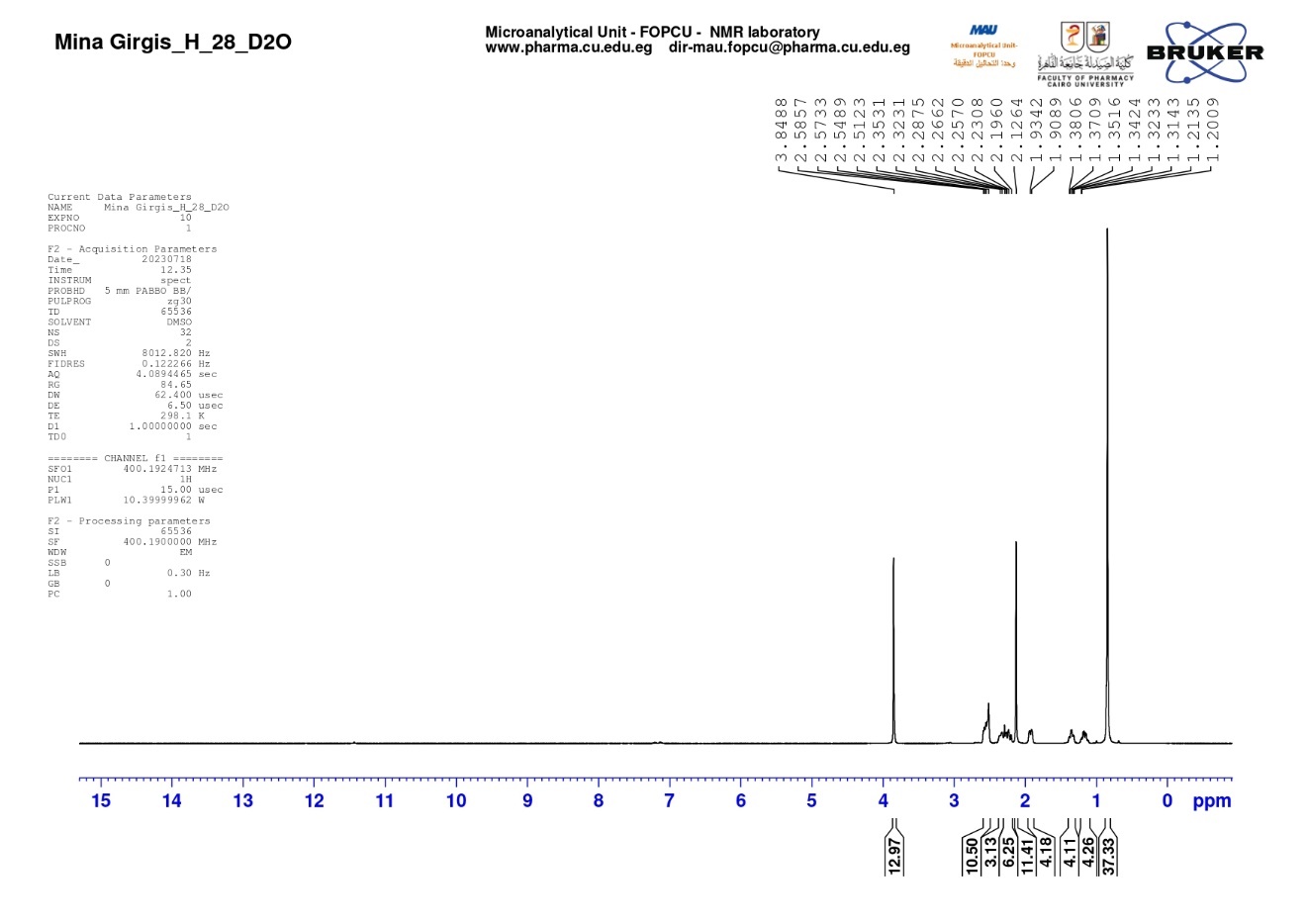
**

**Figure S30**: ^1^H-NMR (DMSO-*d*6+ D_2_O) spectrum of compound **7**

**
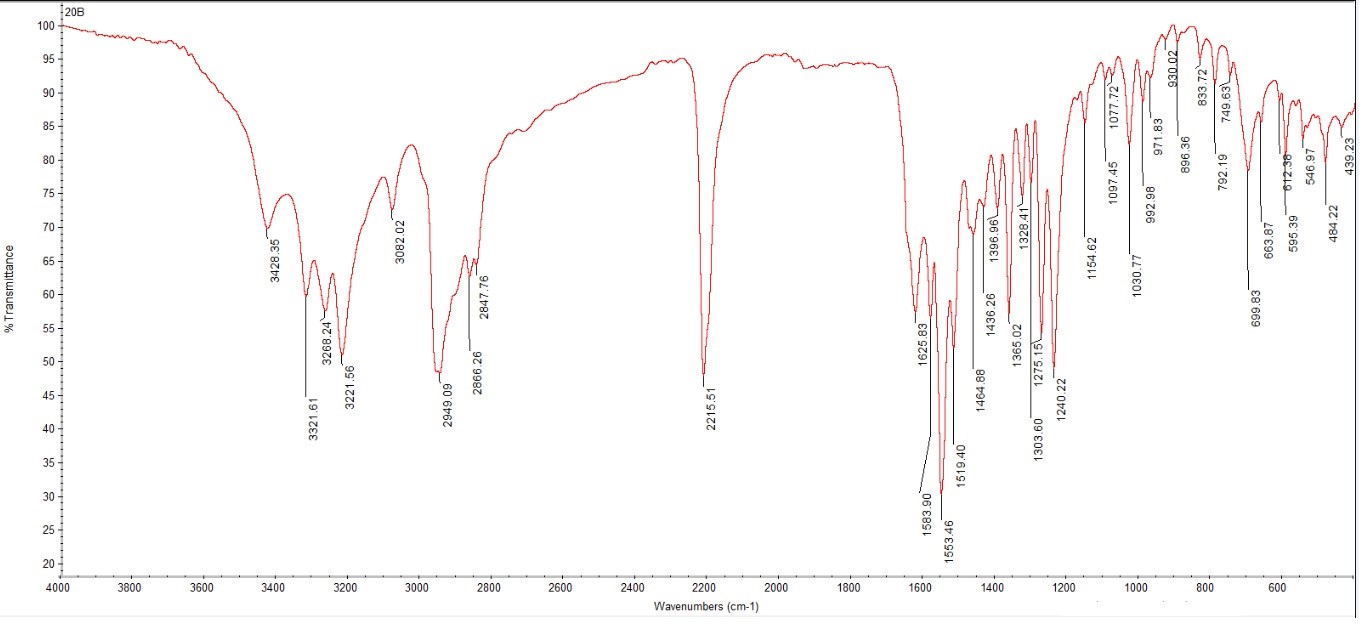
**

**Figure S31**: IR spectrum of compound **8**

**
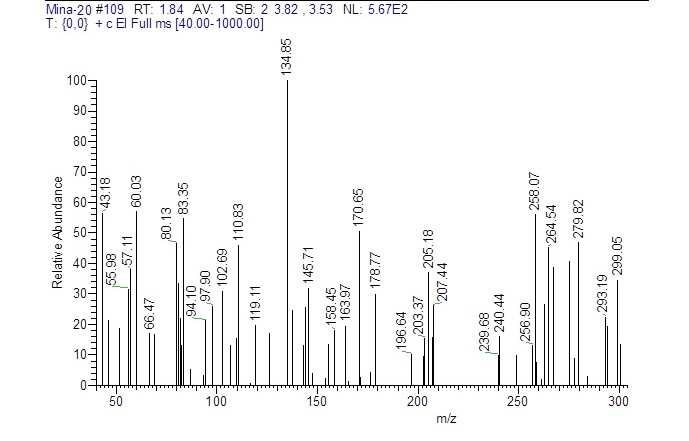
**

**Figure S32**: Mass spectrum of compound **8**

**
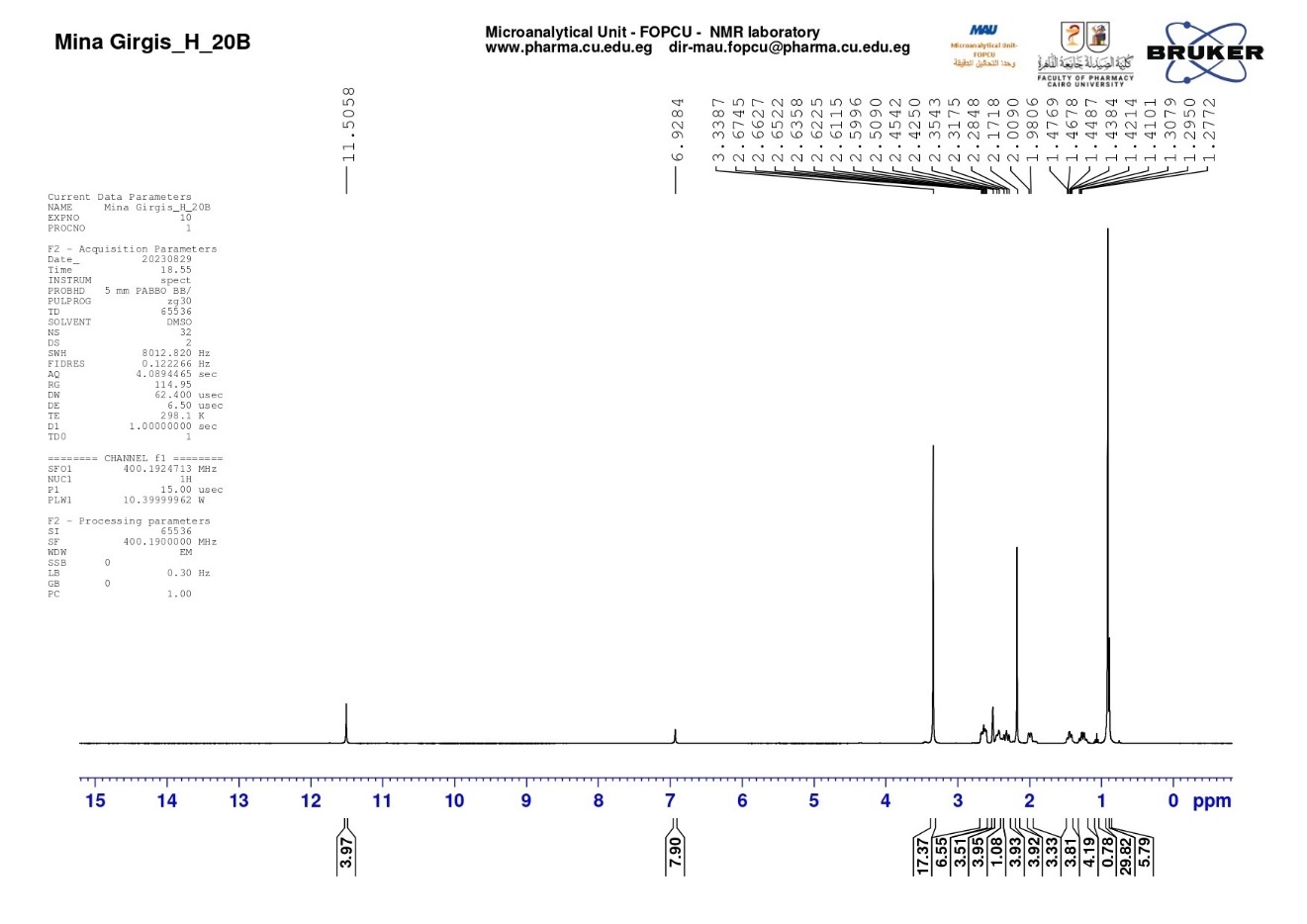
**

**Figure S33**: ^1^H-NMR (DMSO-*d*6) spectrum of compound **8**

**
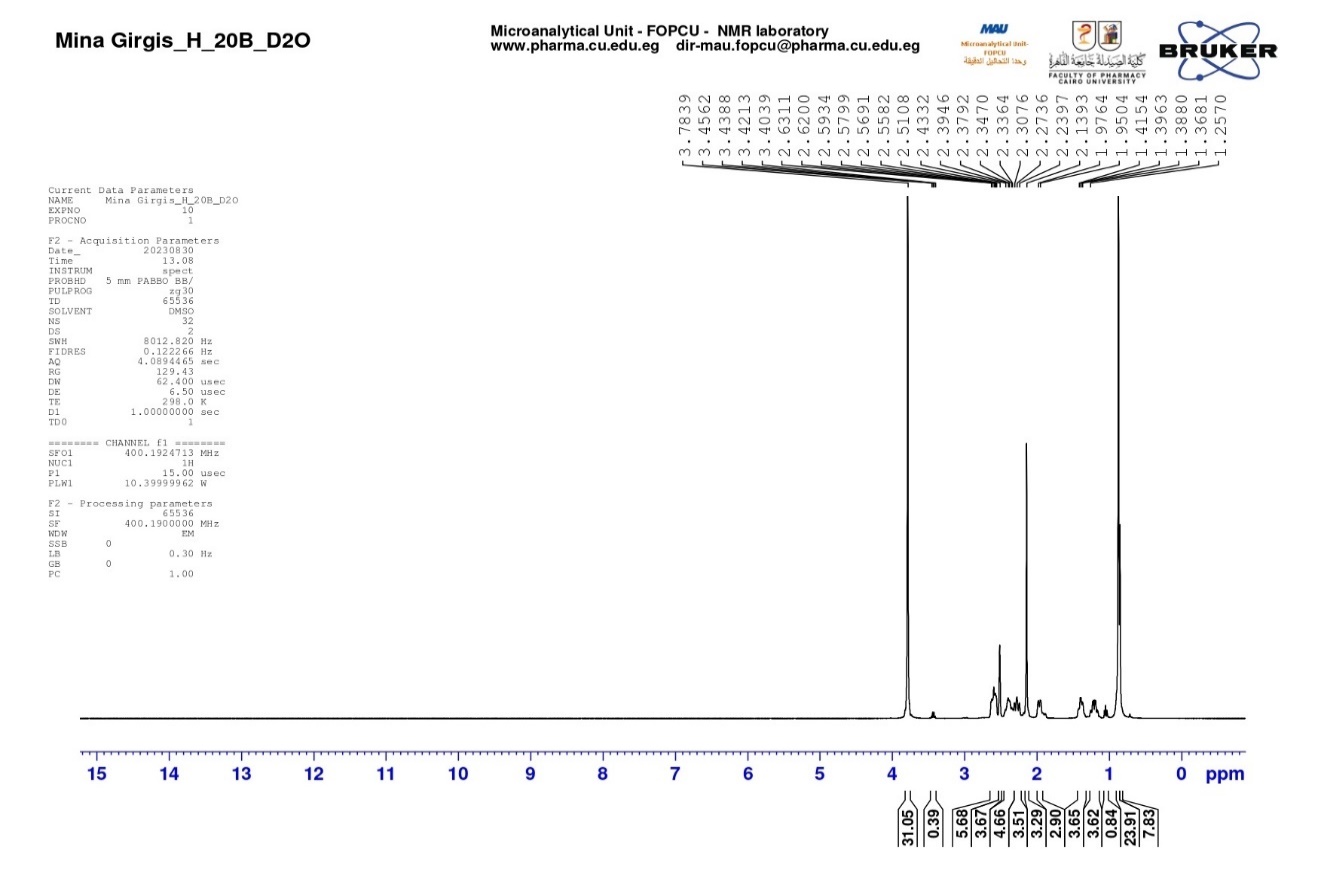
**

**Figure S34**: ^1^H-NMR (DMSO-*d*6+ D_2_O) spectrum of compound **8**

**
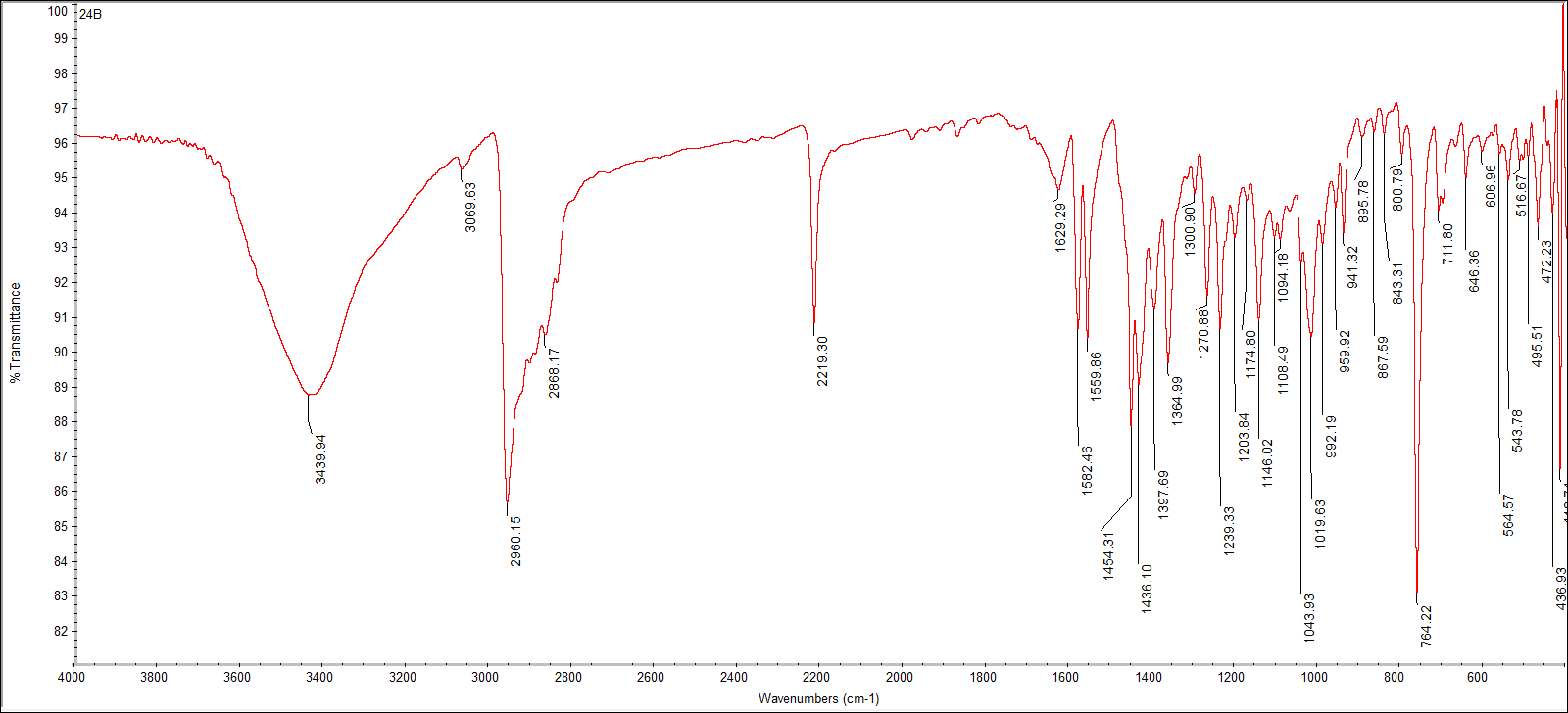
**

**Figure S35**: IR spectrum of compound **9**

**
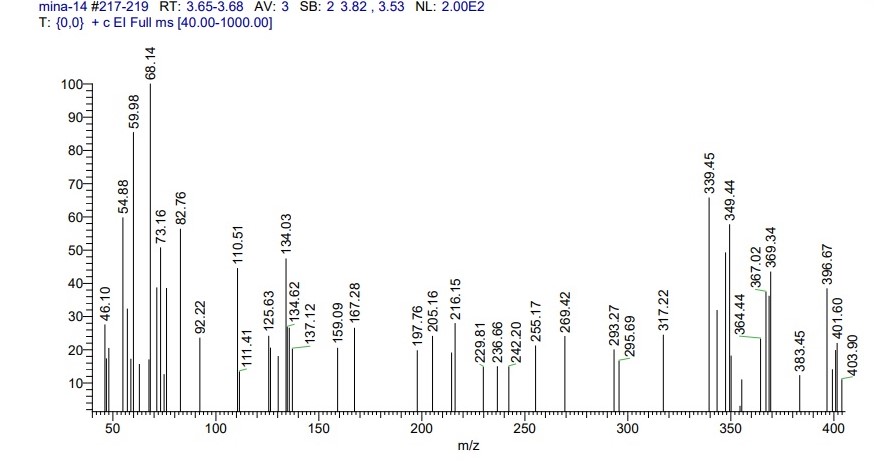
**

**Figure S36**: Mass spectrum of compound **9**

**
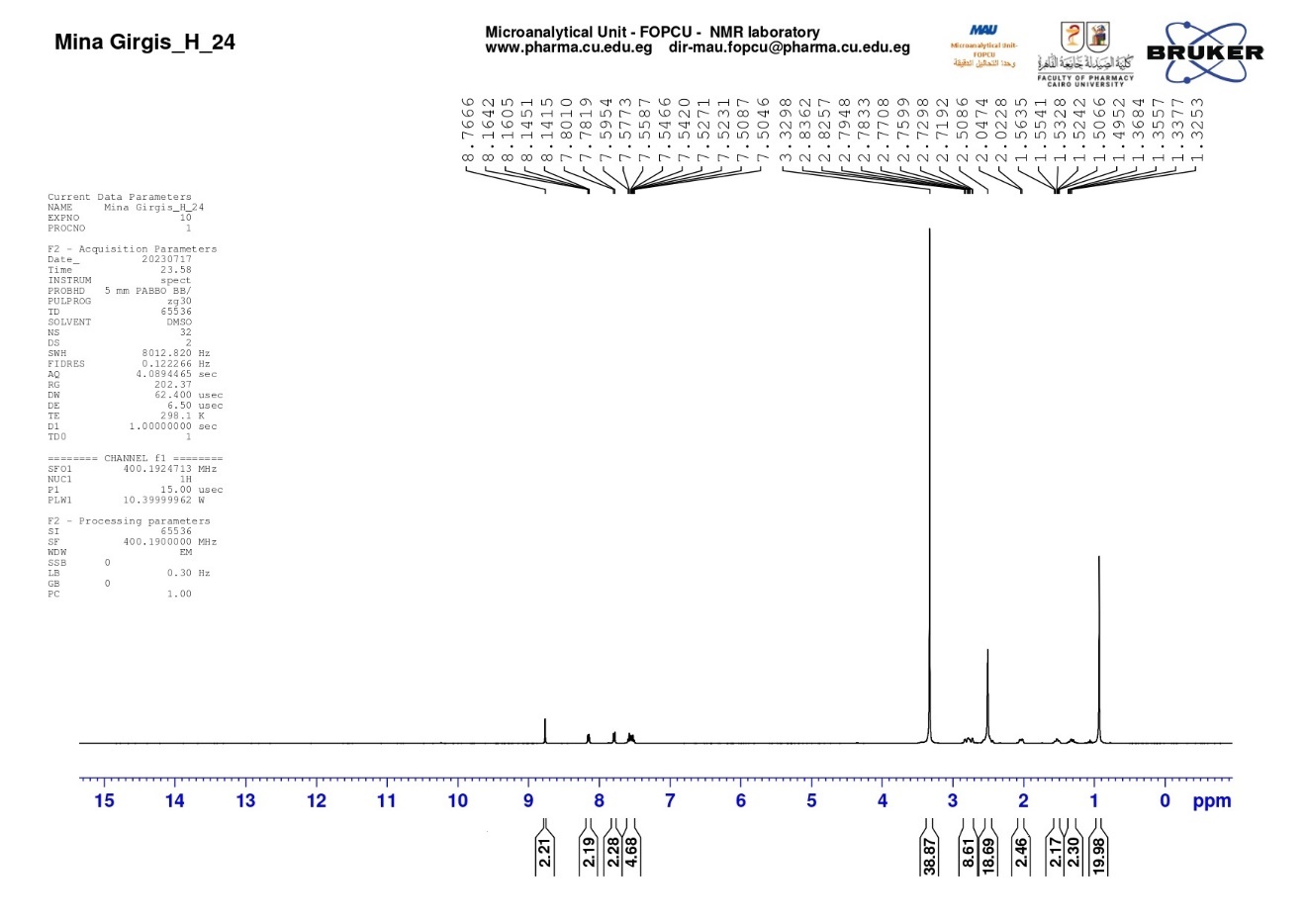
**

**Figure S37**: ^1^H-NMR (DMSO-*d*6) spectrum of compound **9**

**
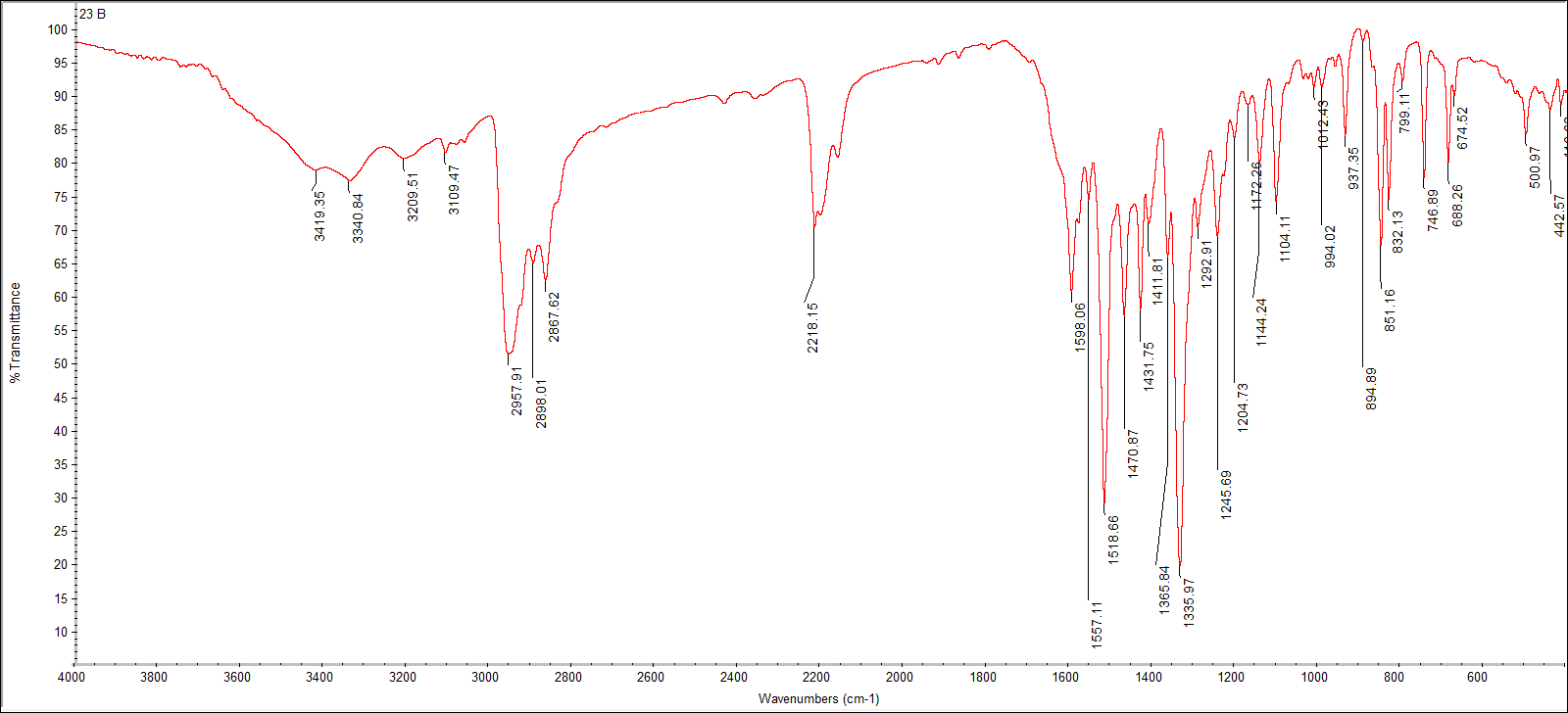
**

**Figure S38**: IR spectrum of compound **10**

**
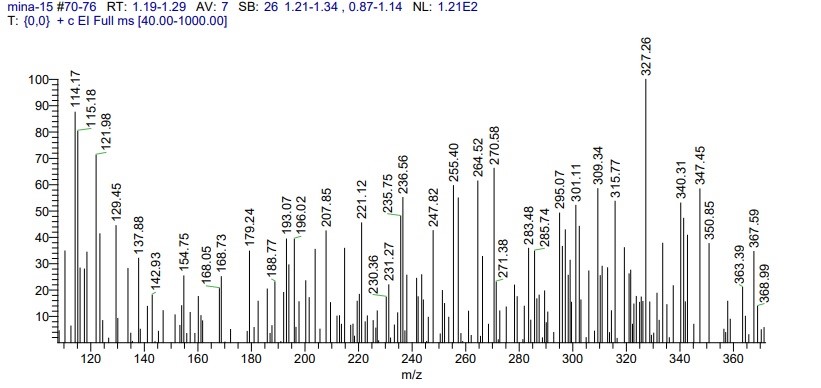
**

**Figure S39**: Mass spectrum of compound **10**

**
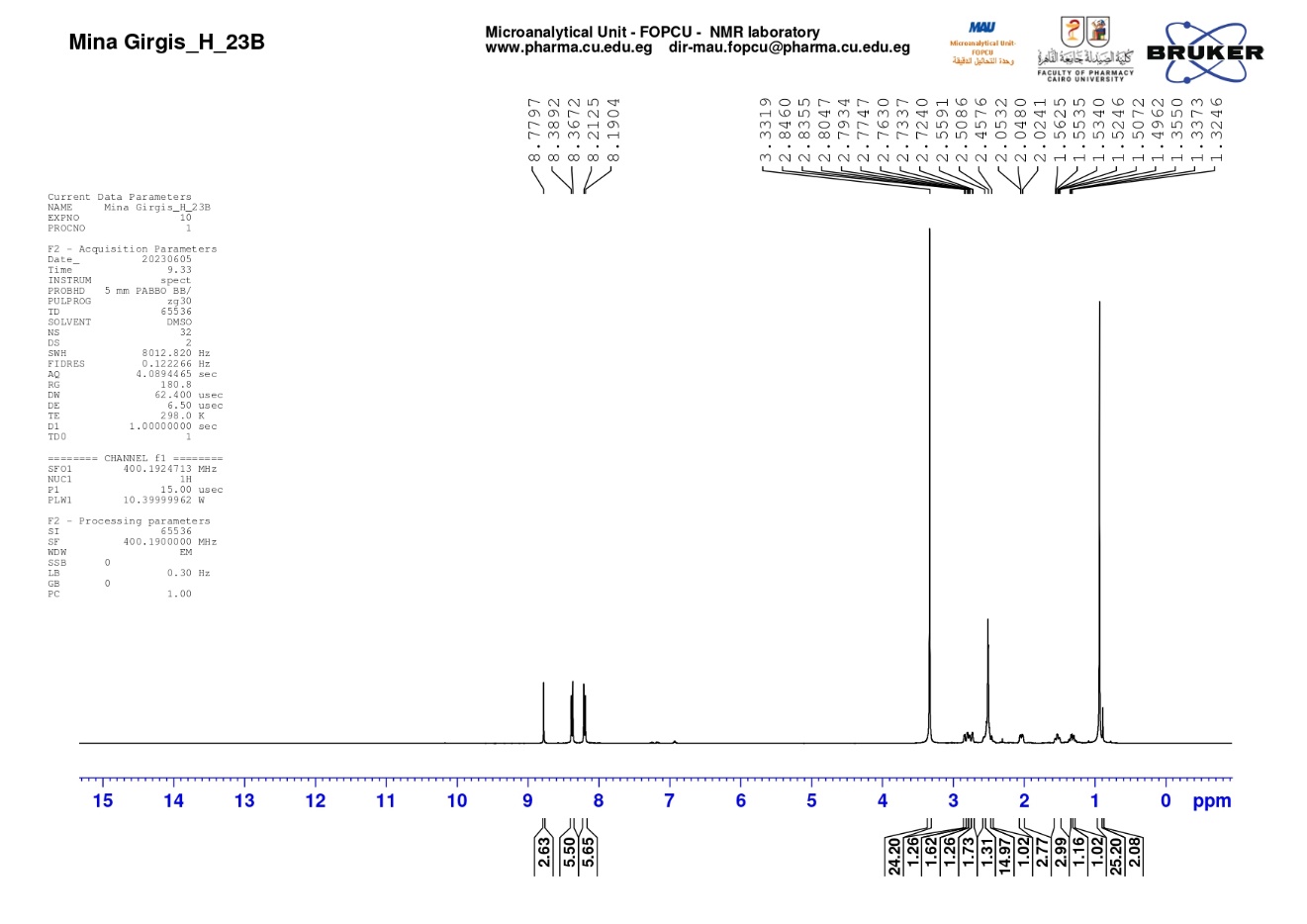
**

**Figure S40**: ^1^H-NMR (DMSO-*d*6) spectrum of compound **10**

**
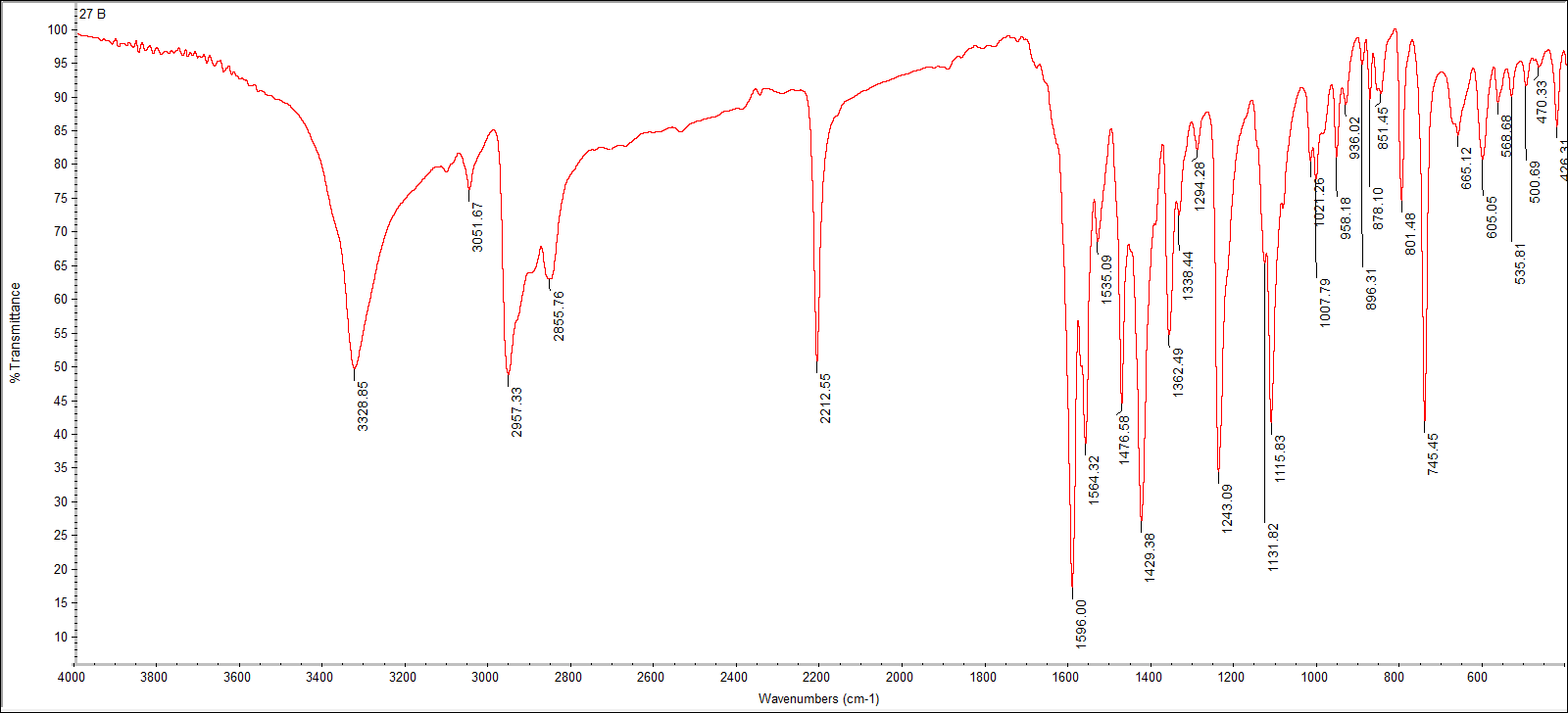
**

**Figure S41**: IR spectrum of compound **11**

**
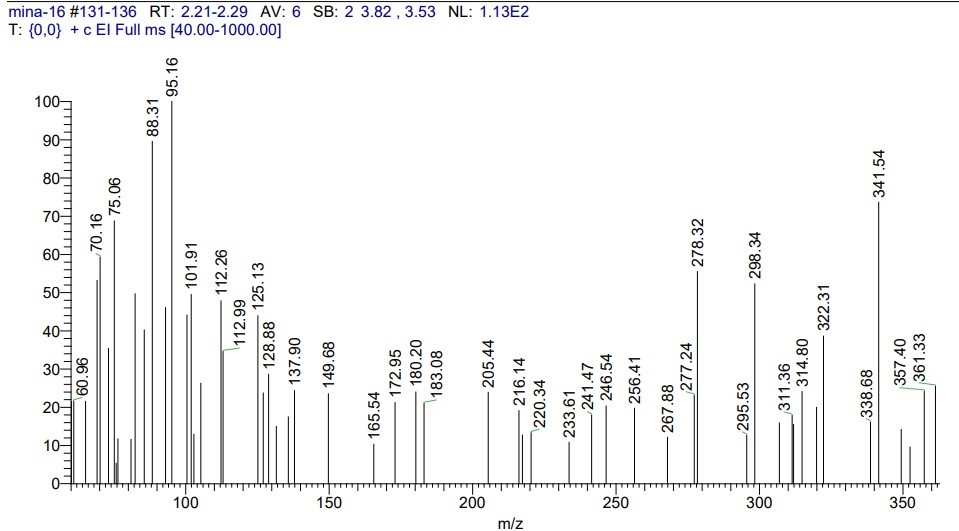
**

**Figure S42**: Mass spectrum of compound **11**

**
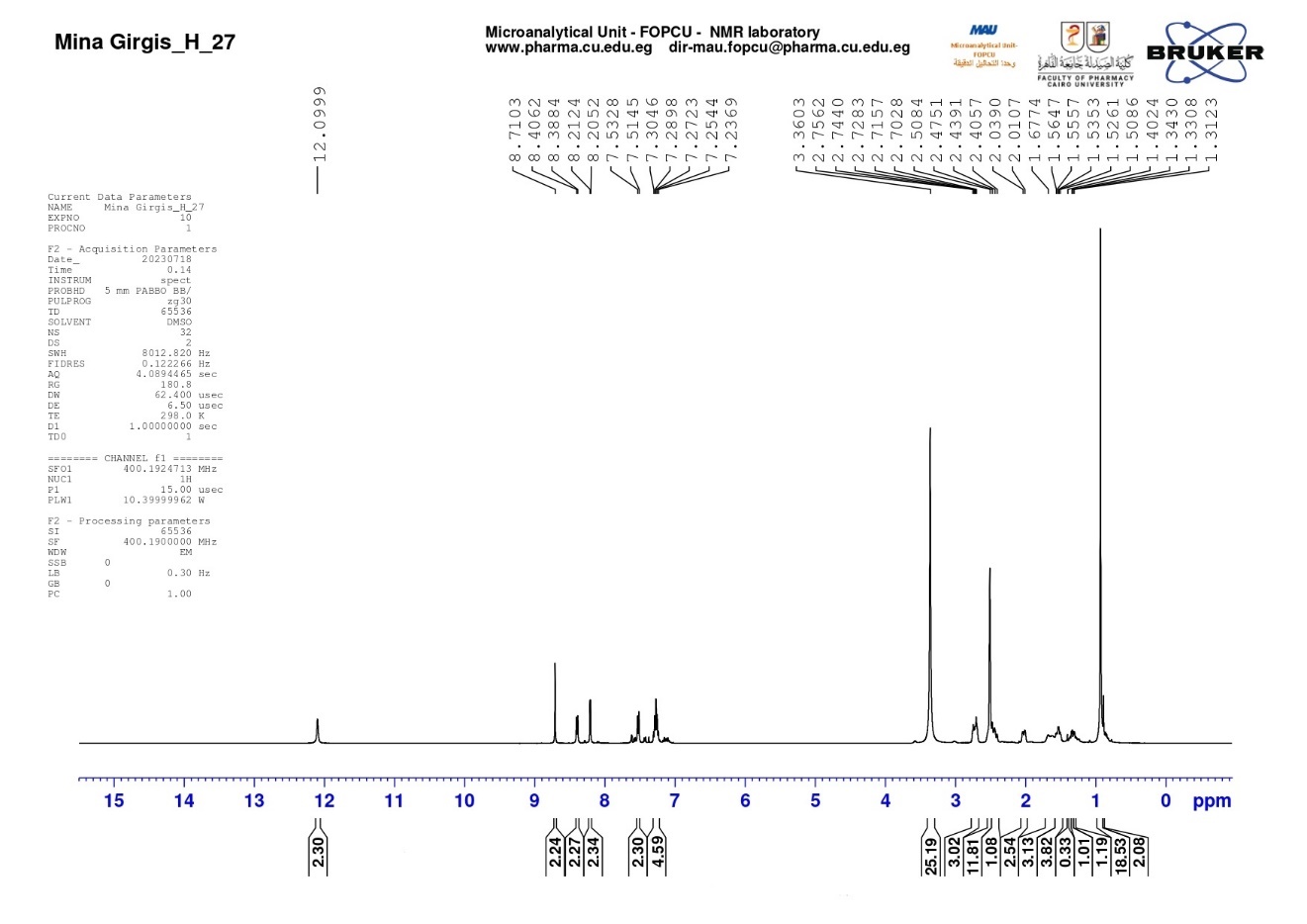
**

**Figure S43**: ^1^H-NMR (DMSO-*d*6) spectrum of compound **11**

**
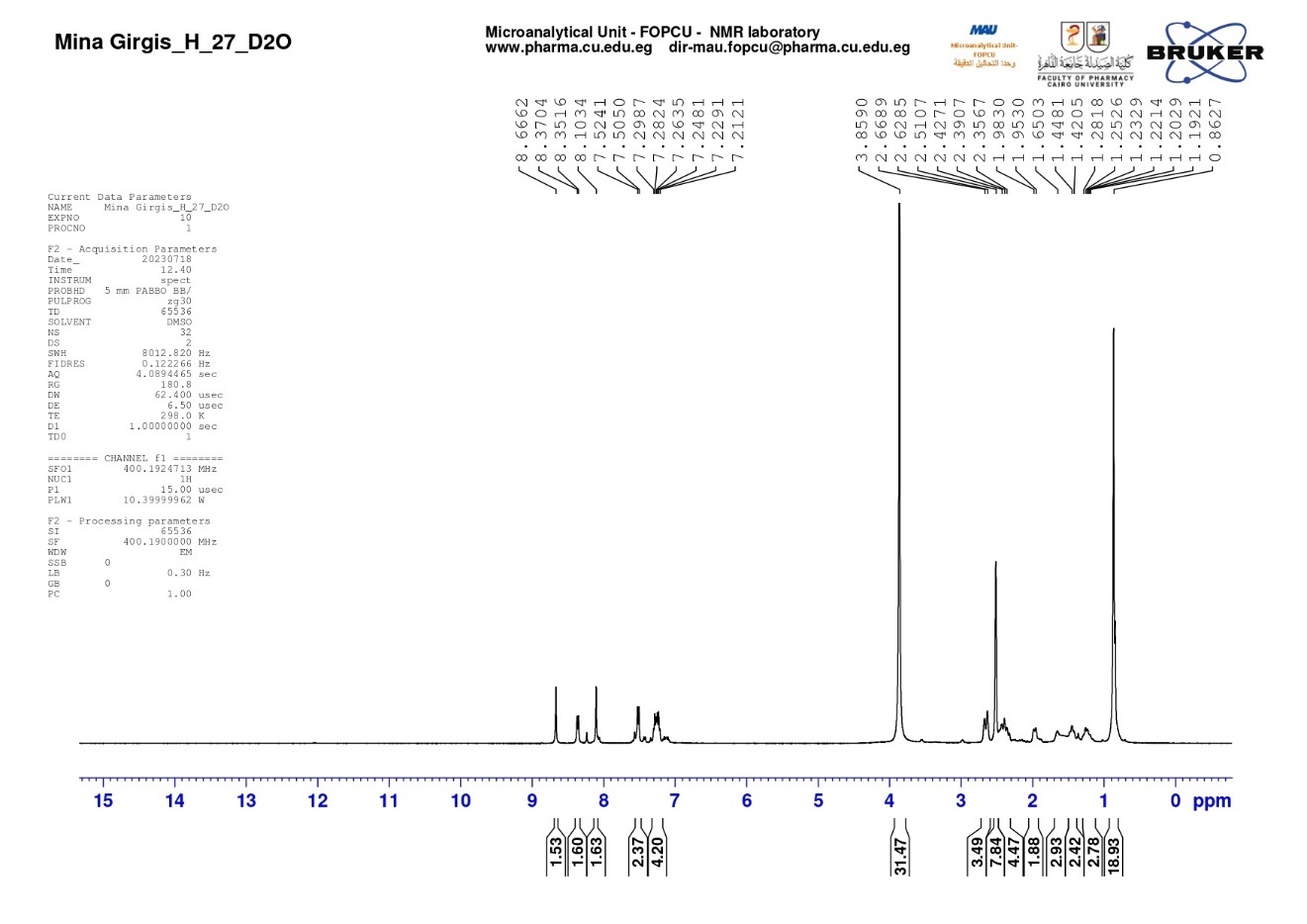
**

**Figure S44**: ^1^H-NMR (DMSO-*d*6+ D_2_O) spectrum of compound **11**

**
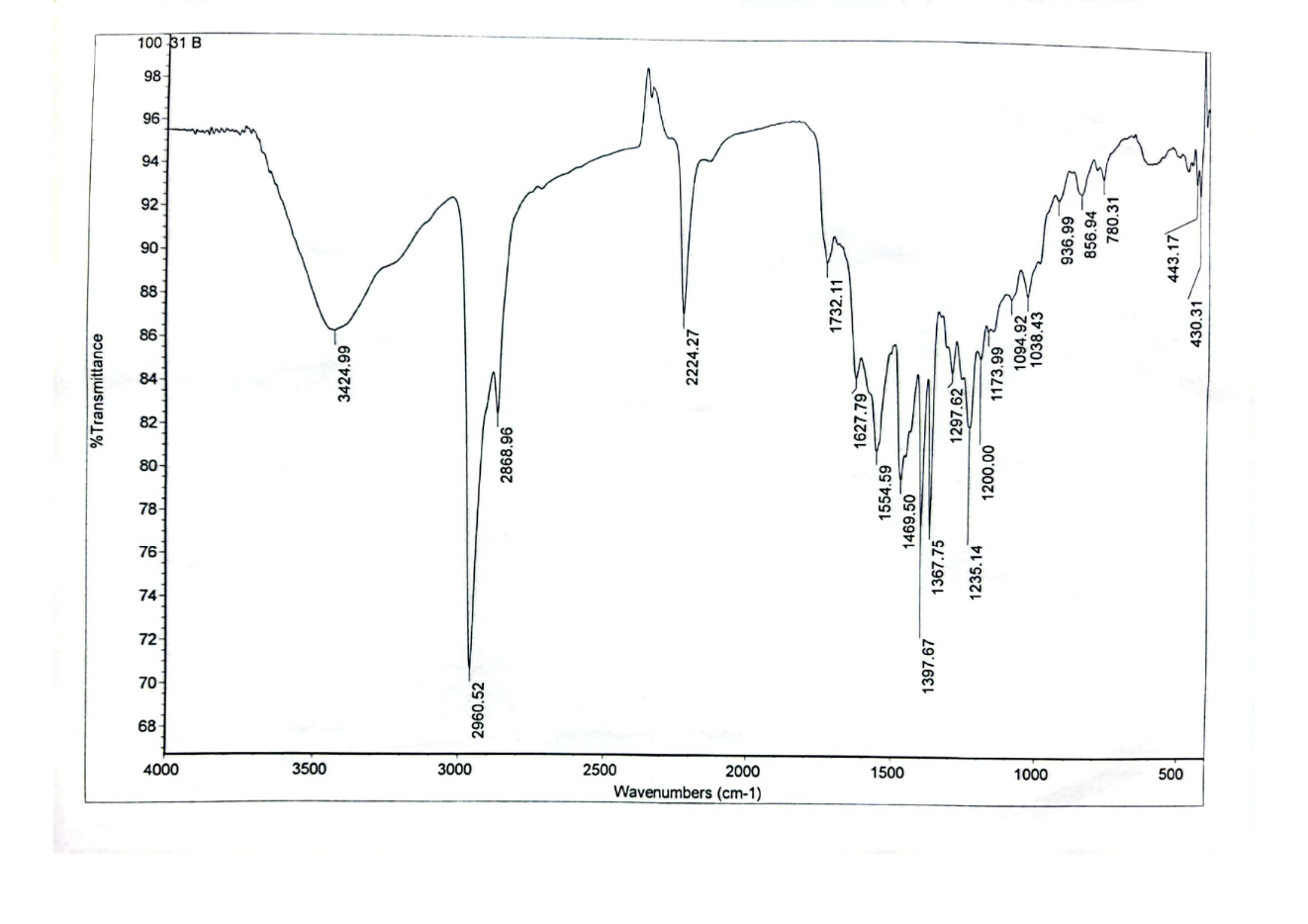
**

**Figure S45**: IR spectrum of compound **13**

**
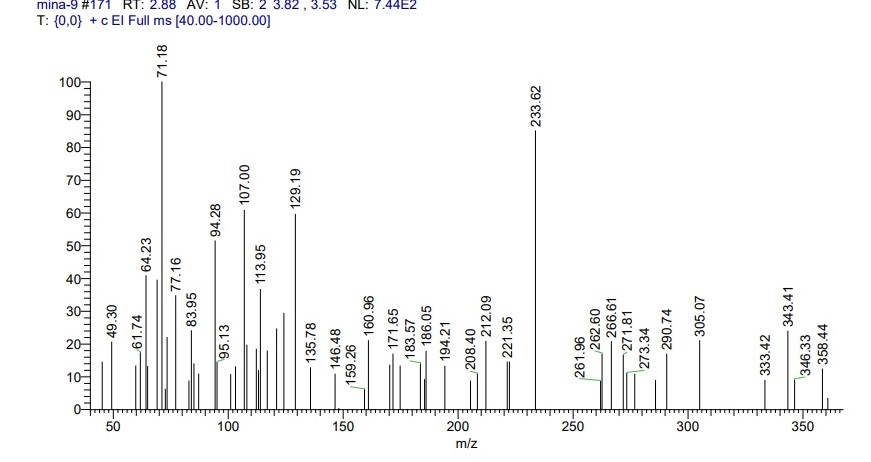
**

**Figure S46**: Mass spectrum of compound **13**

**
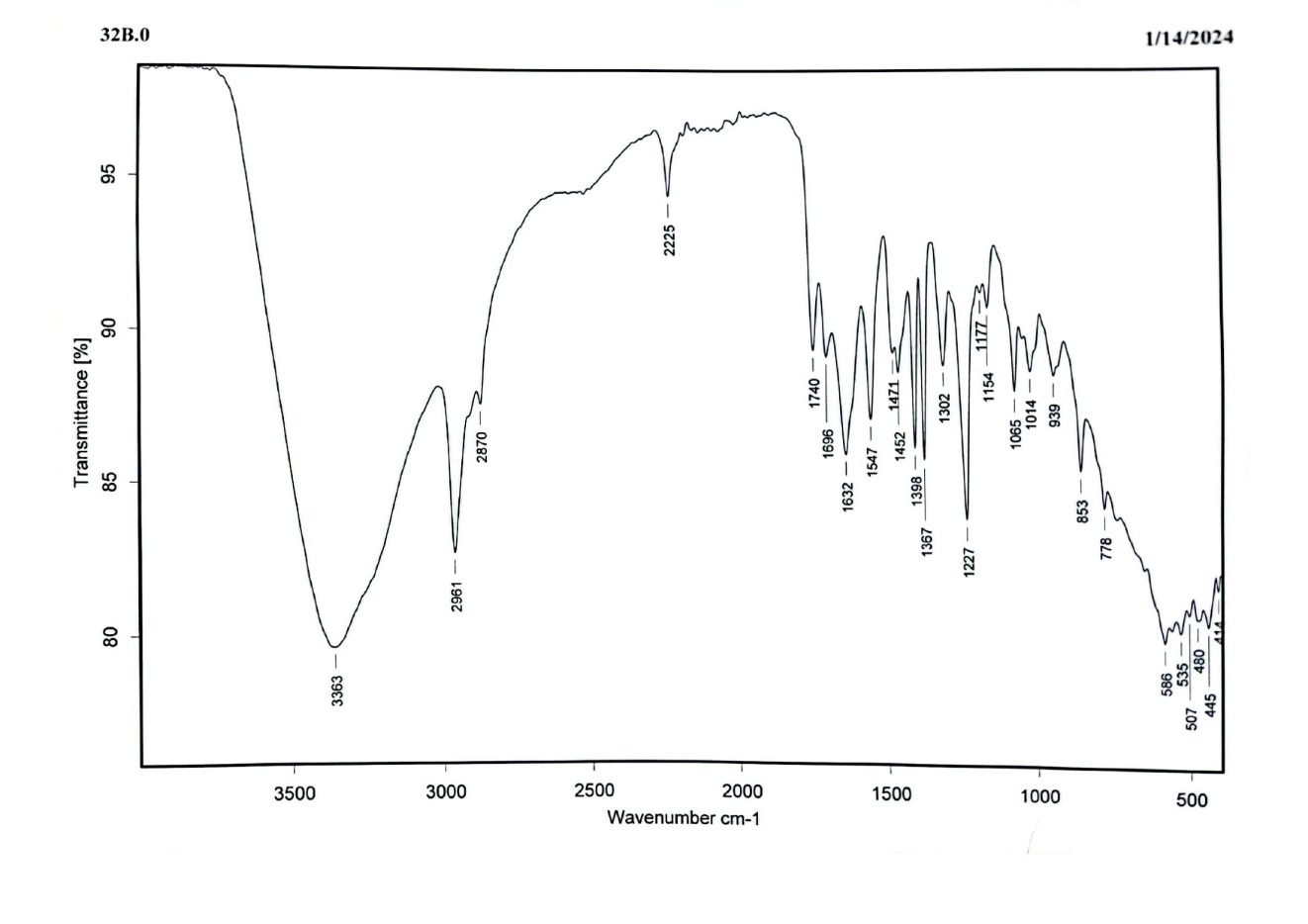
**

**Figure S47**: IR spectrum of compound **14**

**
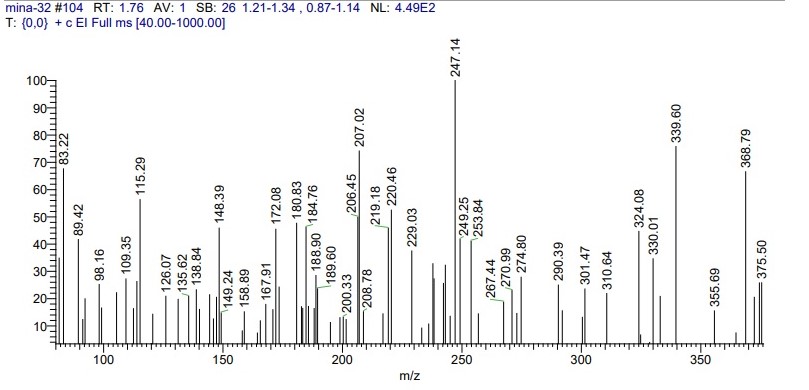
**

**Figure S48**: Mass spectrum of compound **14**

**
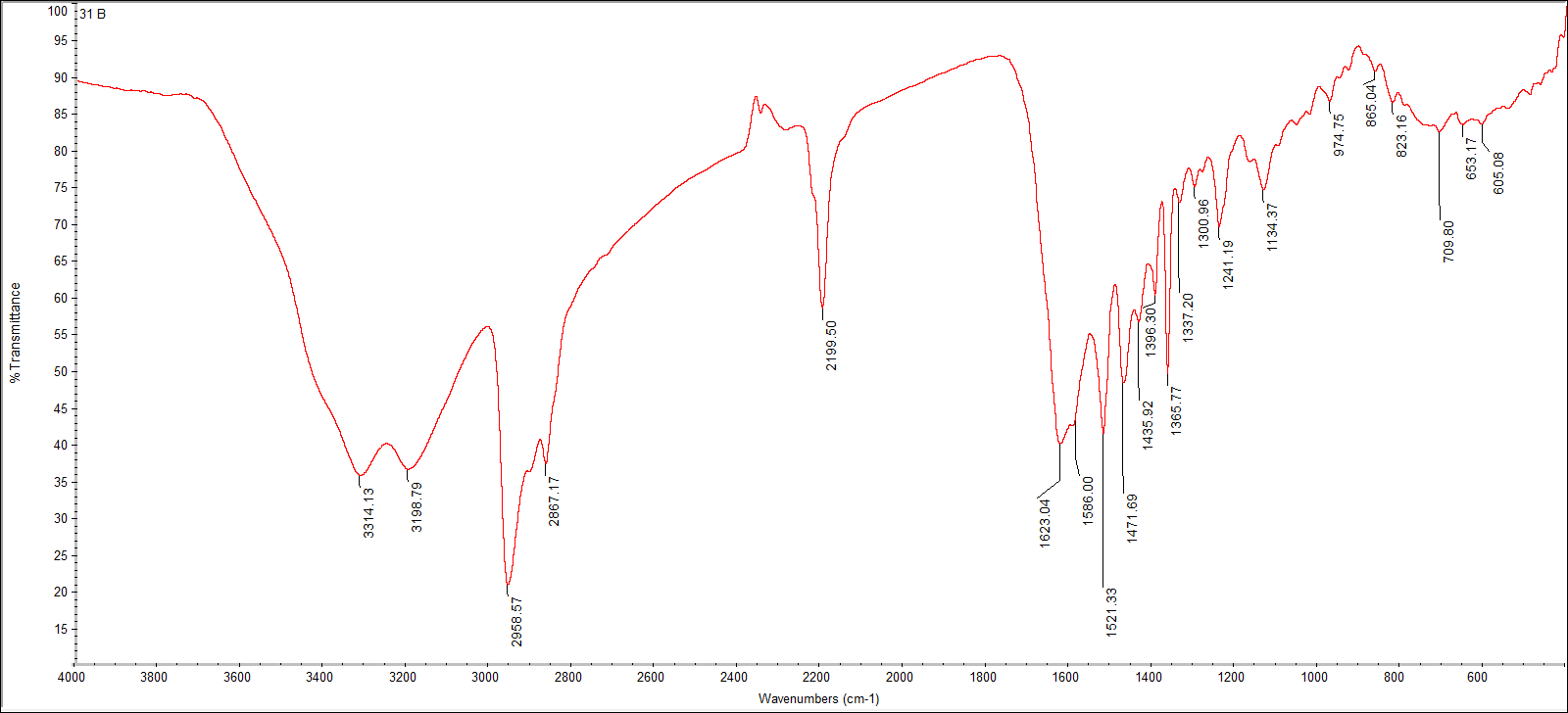
**

**Figure S49**: IR spectrum of compound **15**

**
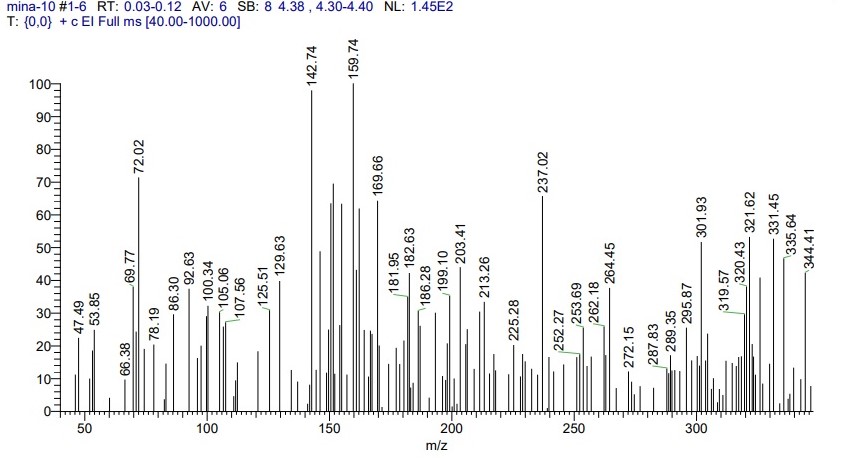
**

**Figure S50**: Mass spectrum of compound **15**

**
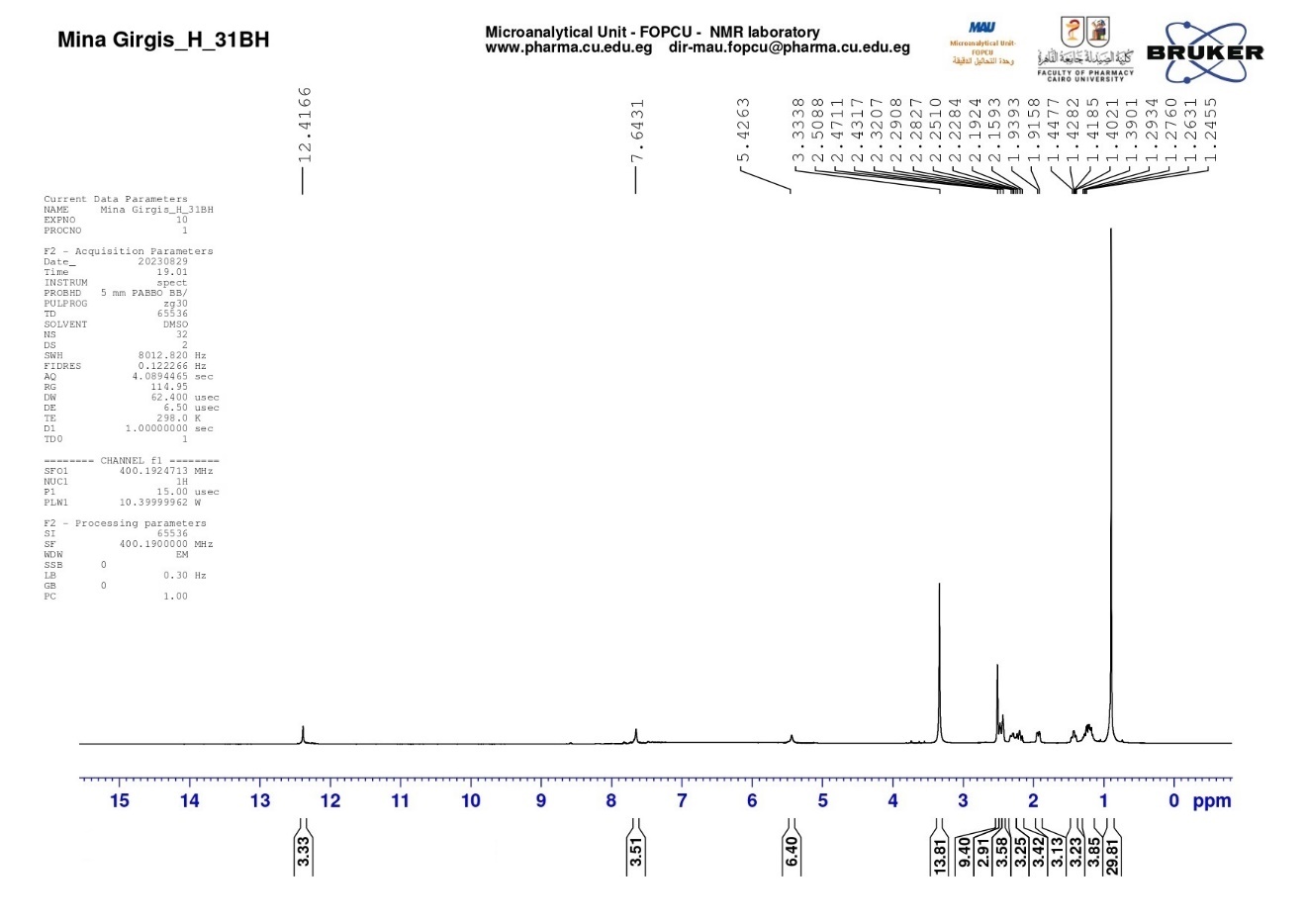
**

**Figure S51**: ^1^H-NMR (DMSO-*d*6) spectrum of compound **15**

**
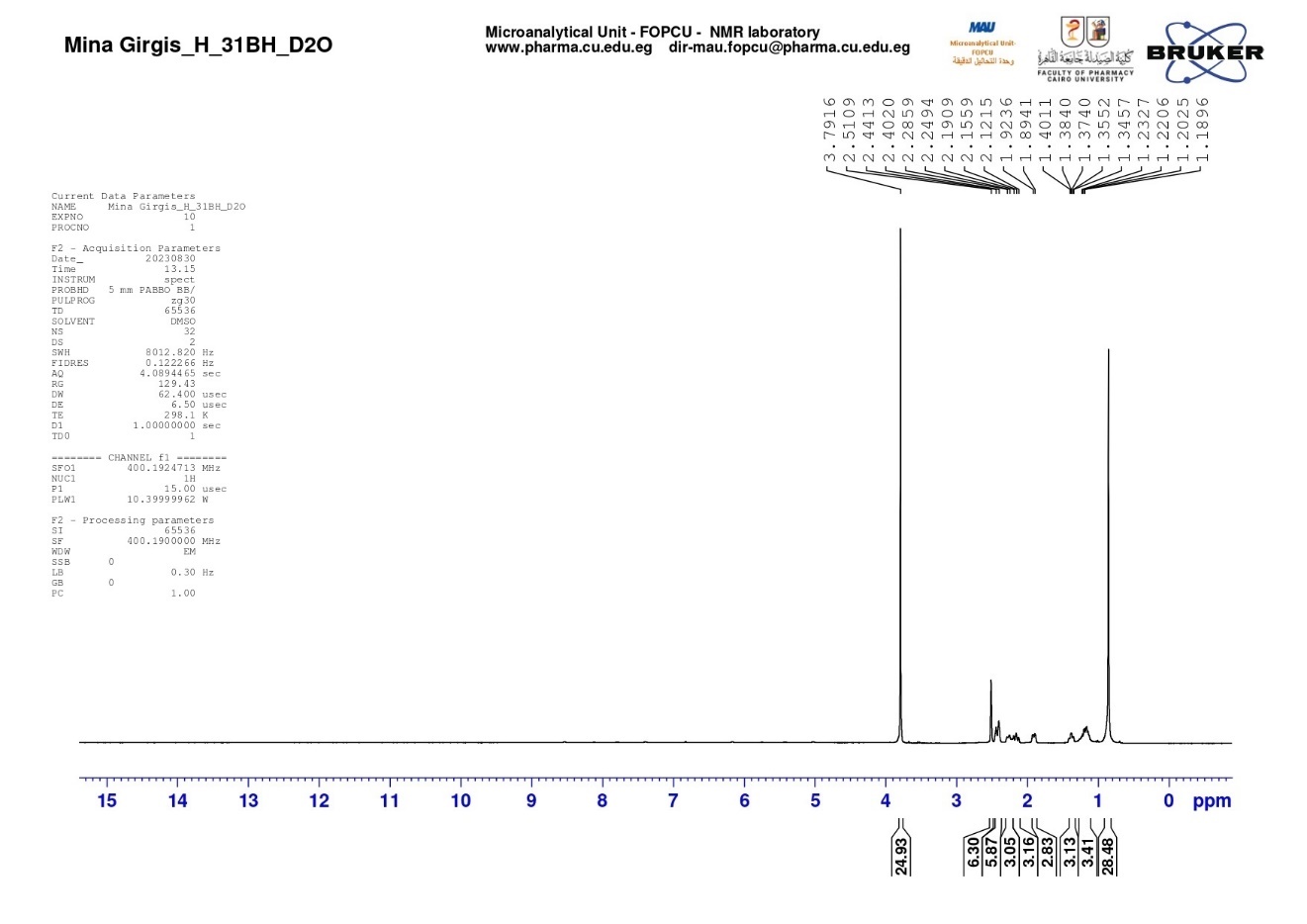
**

**Figure S52**: ^1^H-NMR (DMSO-*d*6+ D_2_O) spectrum of compound **15**

**
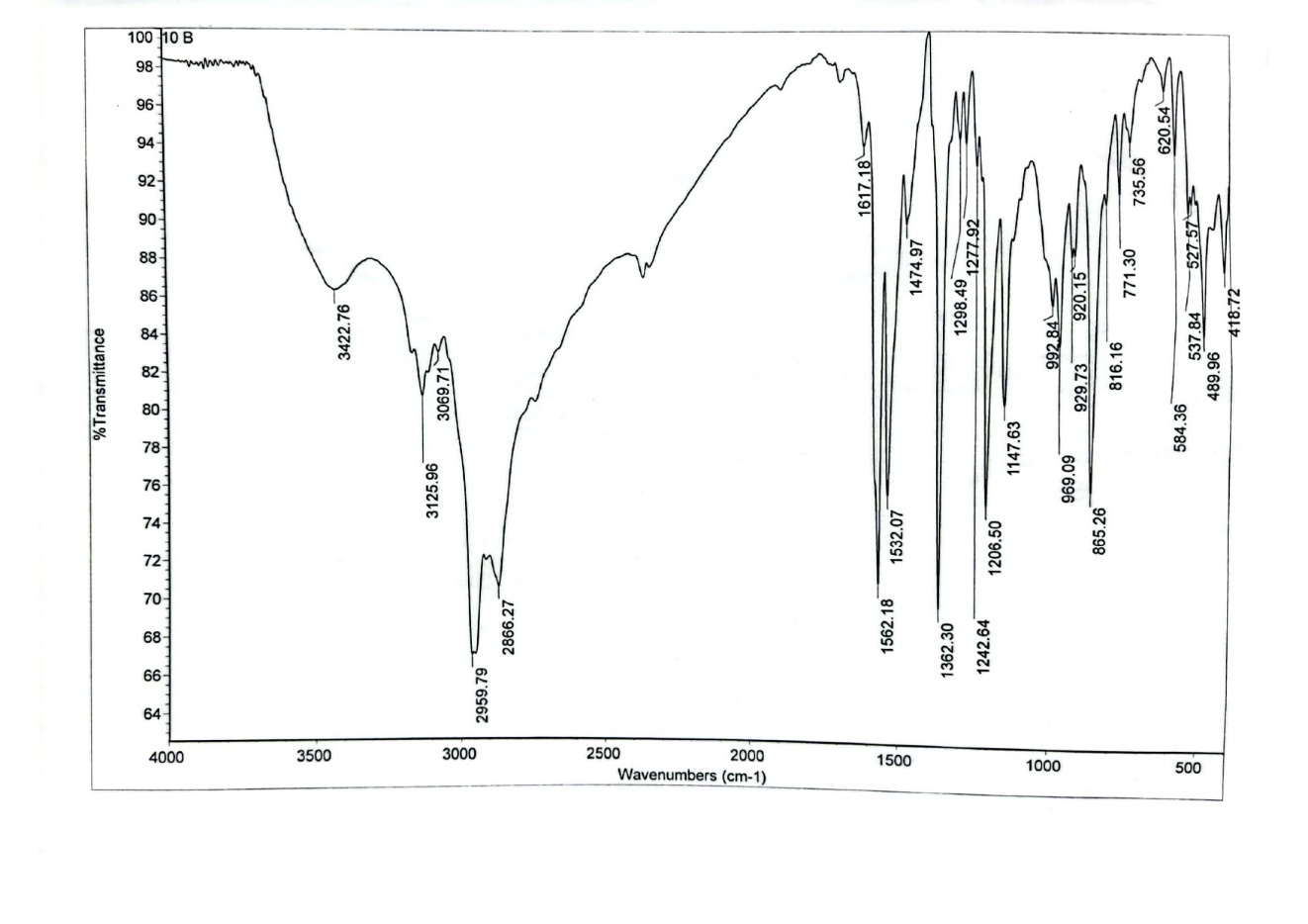
**

**Figure S53**: IR spectrum of compound **16**

**
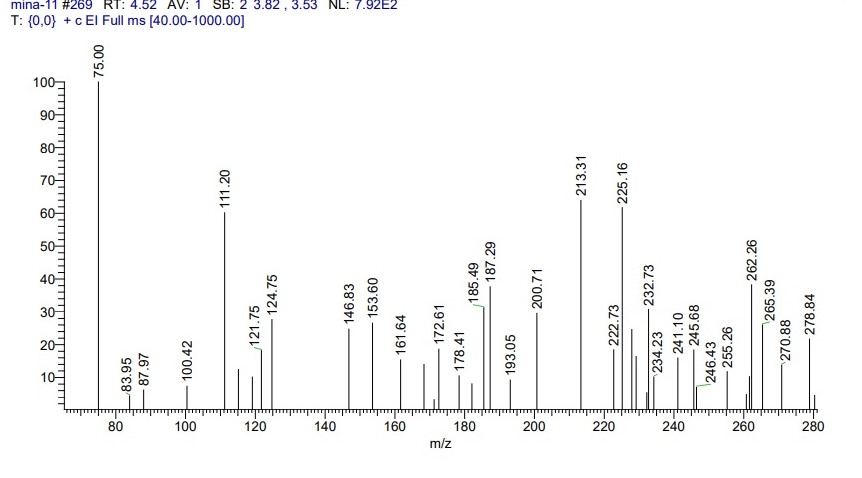
**

**Figure S54**: Mass spectrum of compound **16**

**
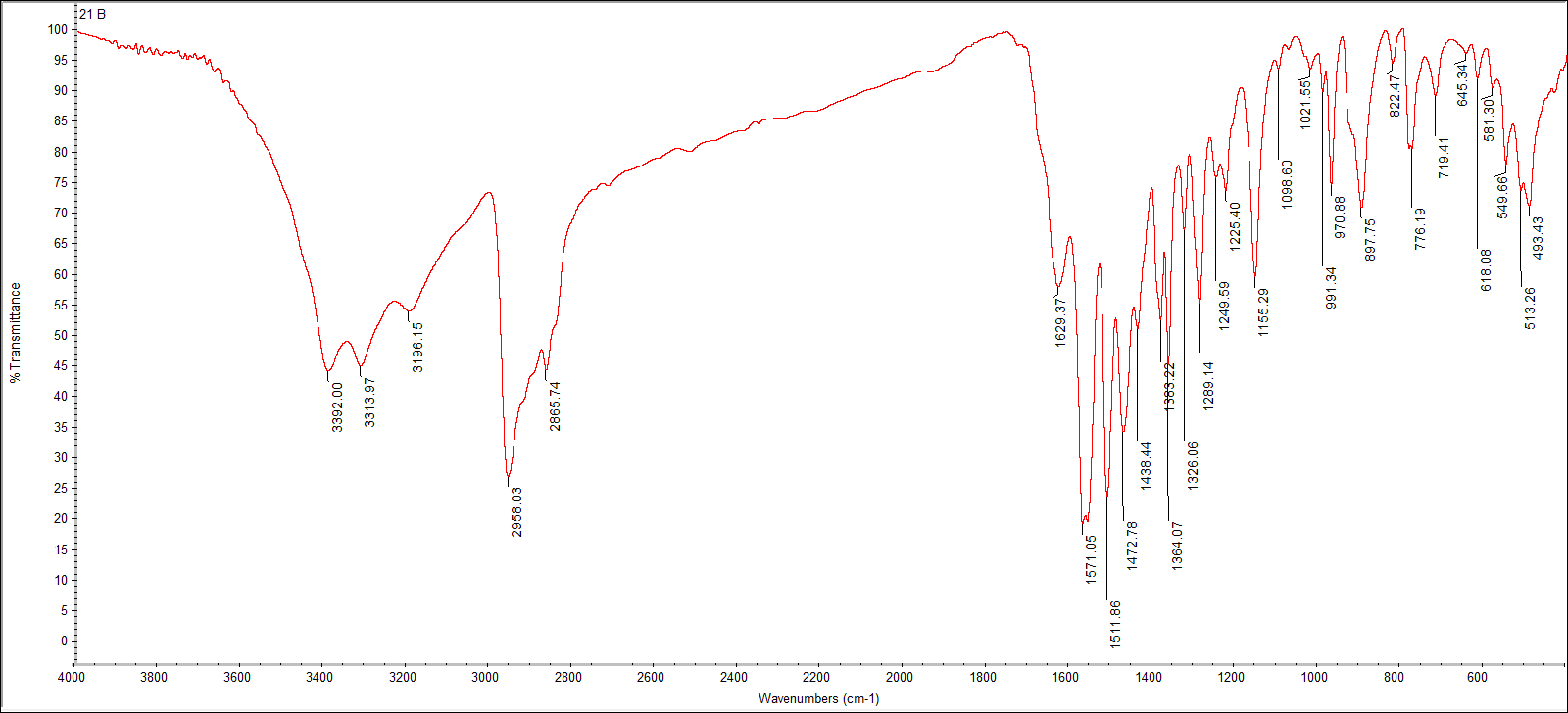
**

**Figure S55**: IR spectrum of compound **17**

**
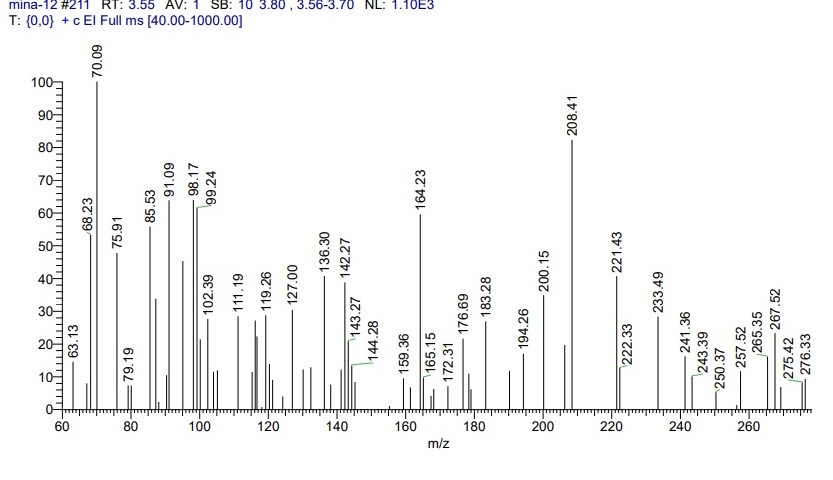
**

**Figure S56**: Mass spectrum of compound **17**

**
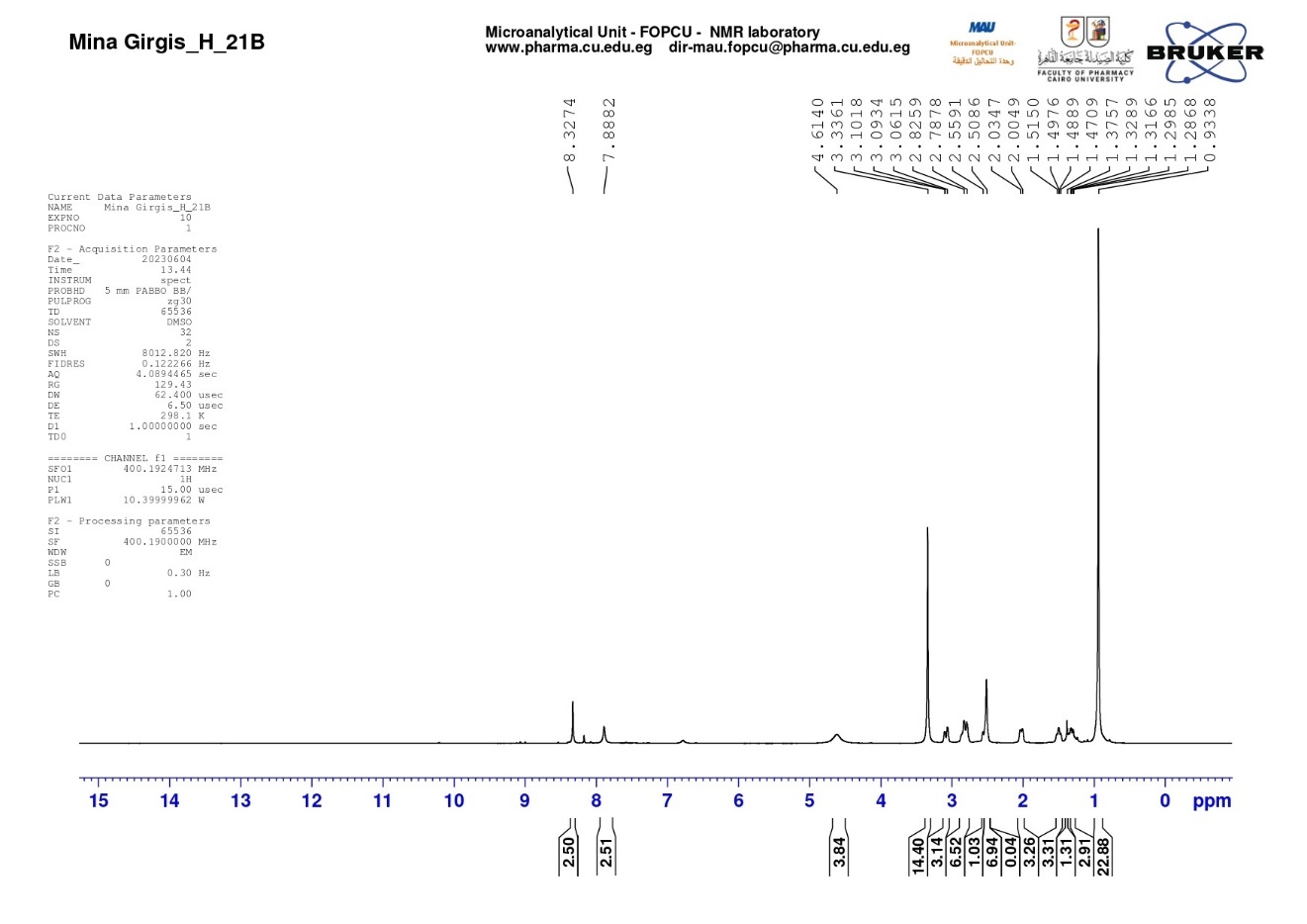
**

**Figure S57**: ^1^H-NMR (DMSO-*d*6) spectrum of compound **17**

**
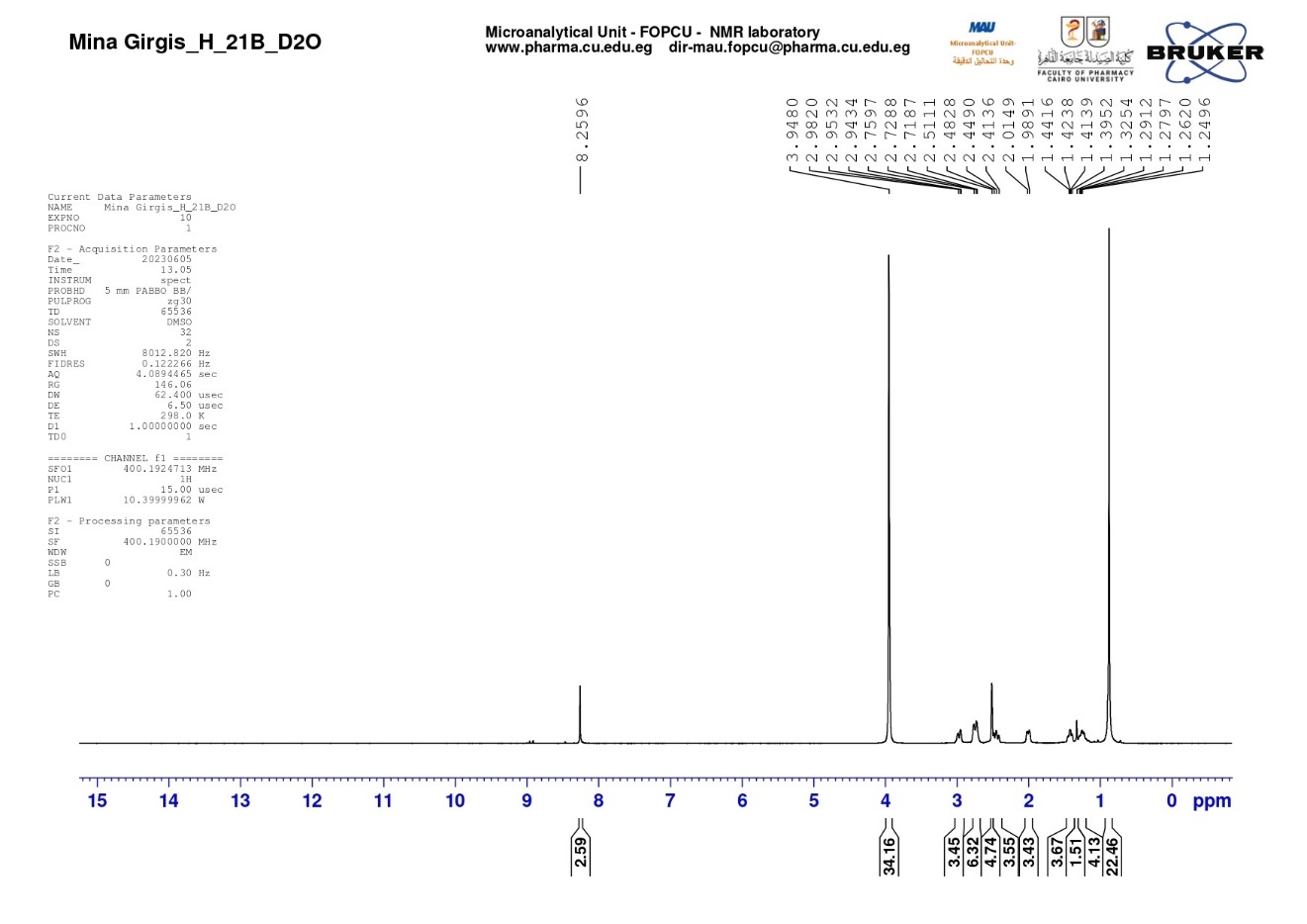
**

**Figure S58**: ^1^H-NMR (DMSO-*d*6+ D_2_O) spectrum of compound **17**

**
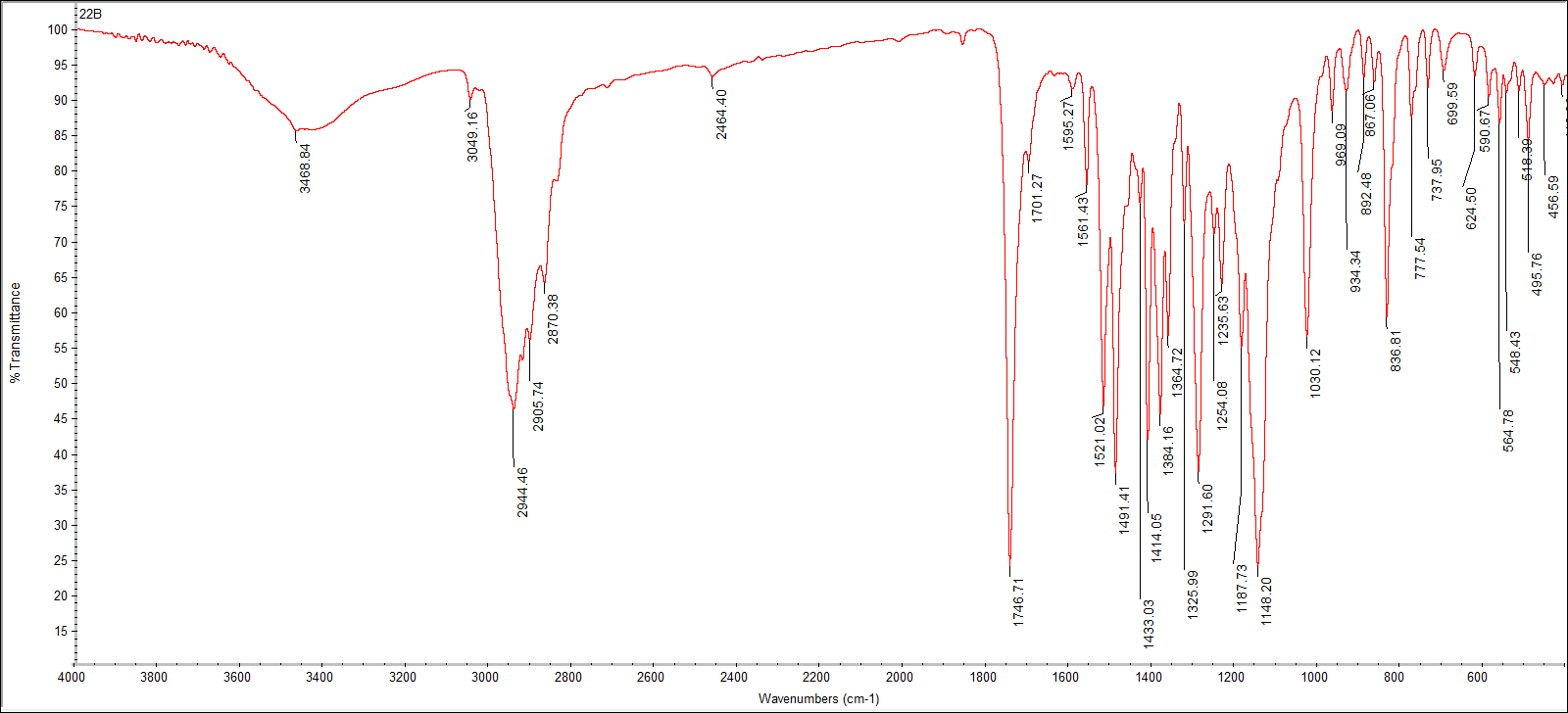
**

**Figure S59**: IR spectrum of compound **18**

**
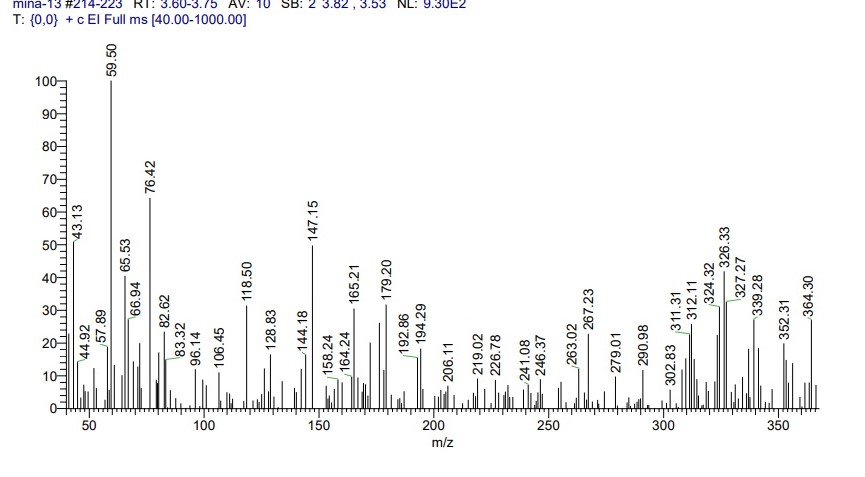
**

**Figure S60**: Mass spectrum of compound **18**

**
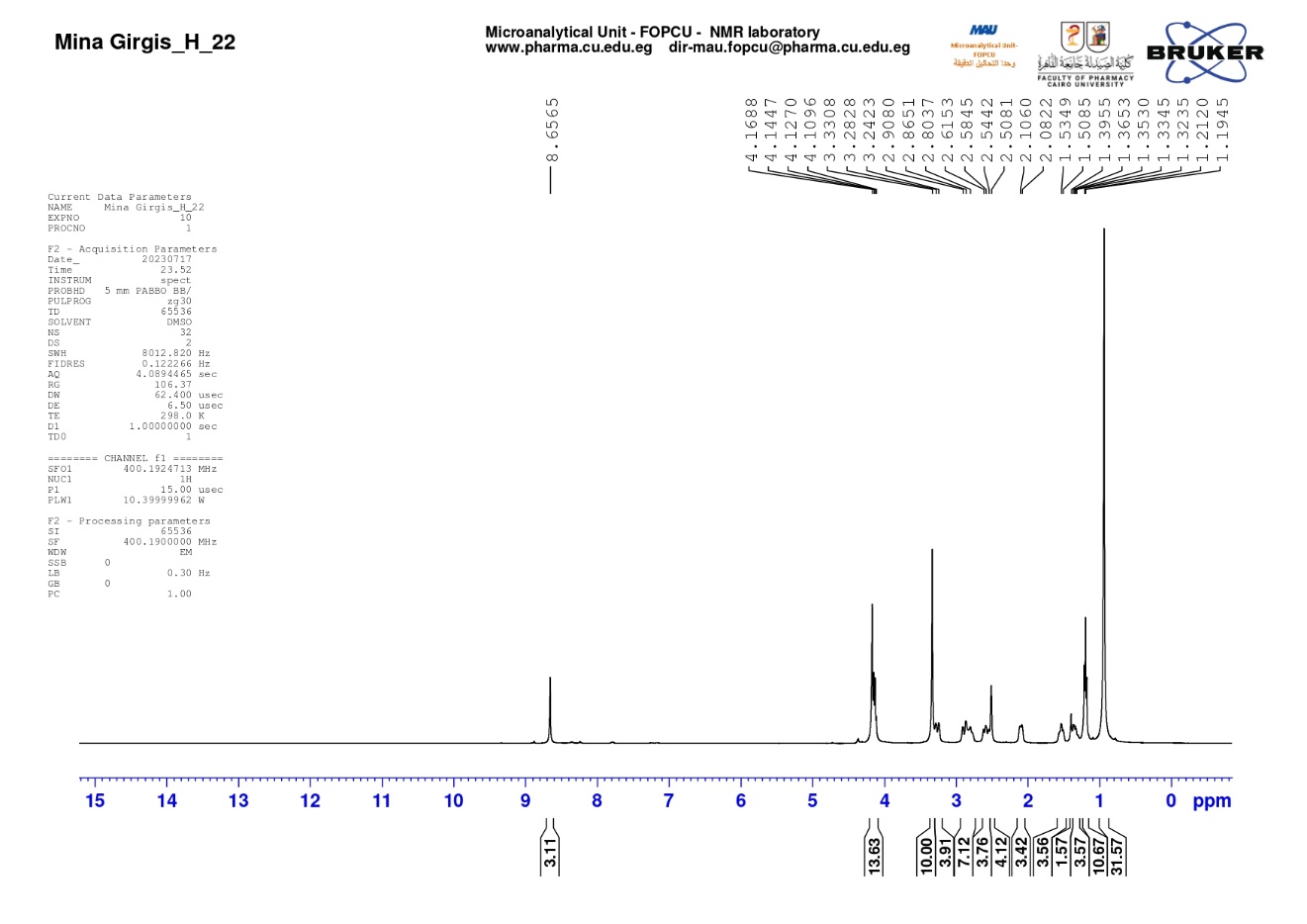
**

**Figure S61**: ^1^H-NMR (DMSO-*d*6) spectrum of compound **18**

**
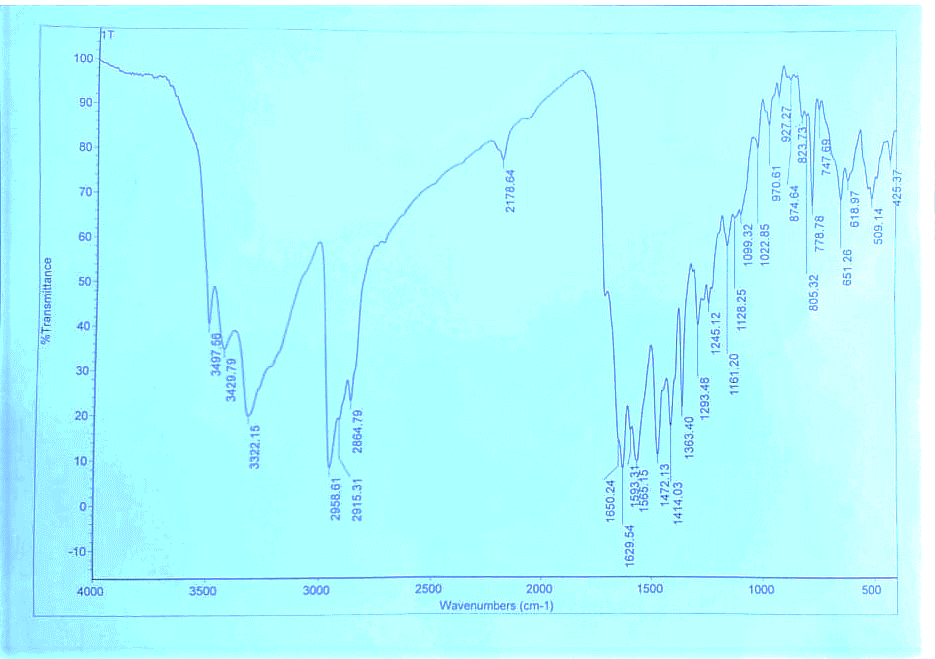
**

**Figure S62**: IR spectrum of compound **19**

**
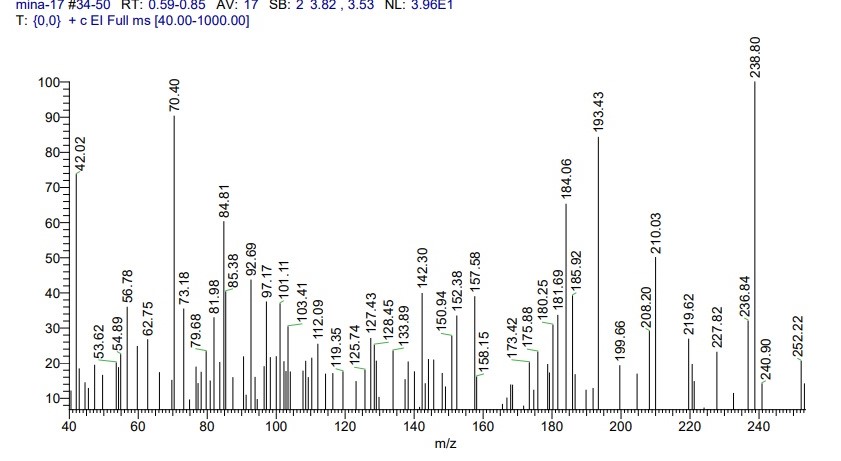
**

**Figure S63**: Mass spectrum of compound **19**


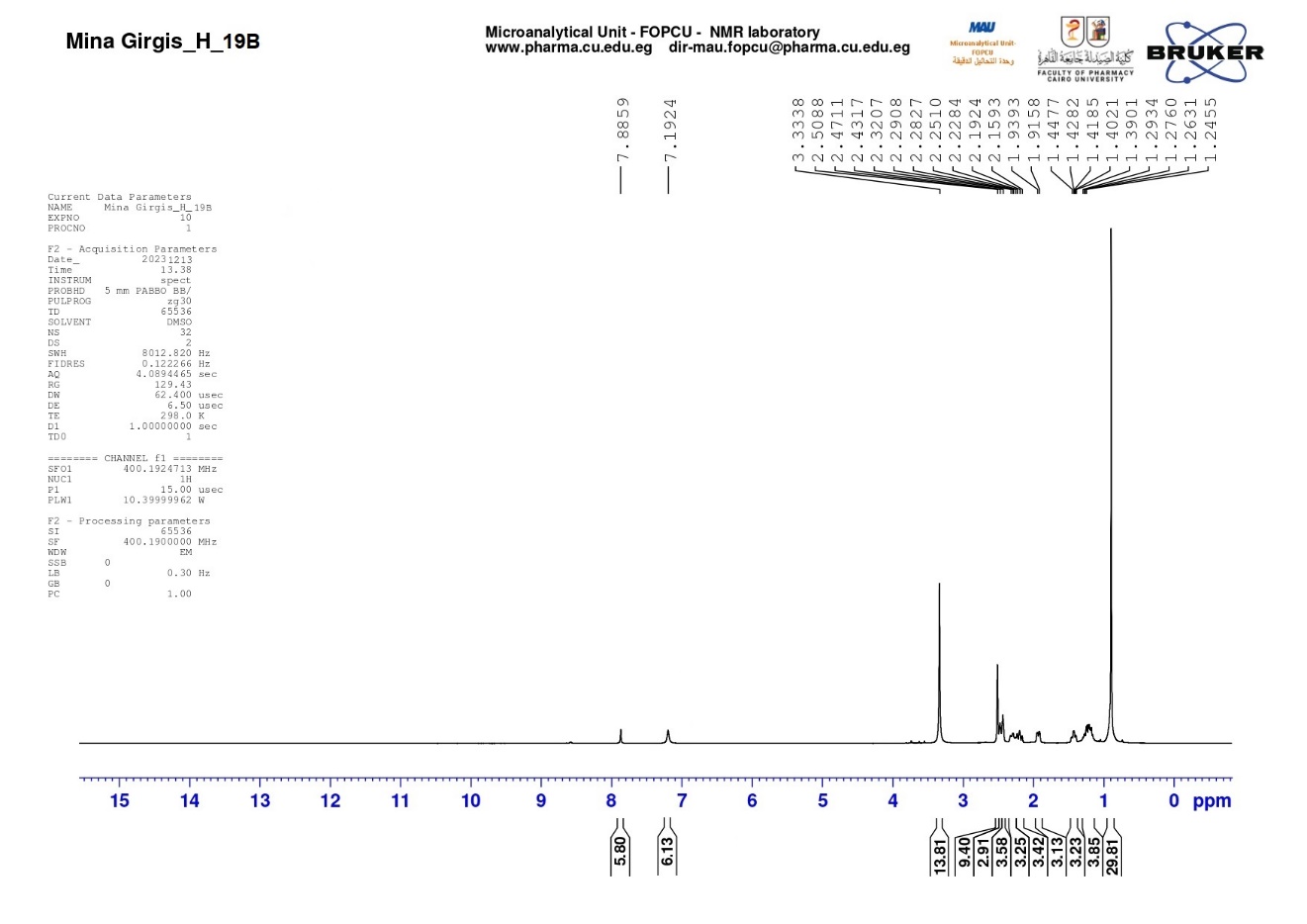


**Figure S64**: ^1^H-NMR (DMSO-*d*6) spectrum of compound **19**


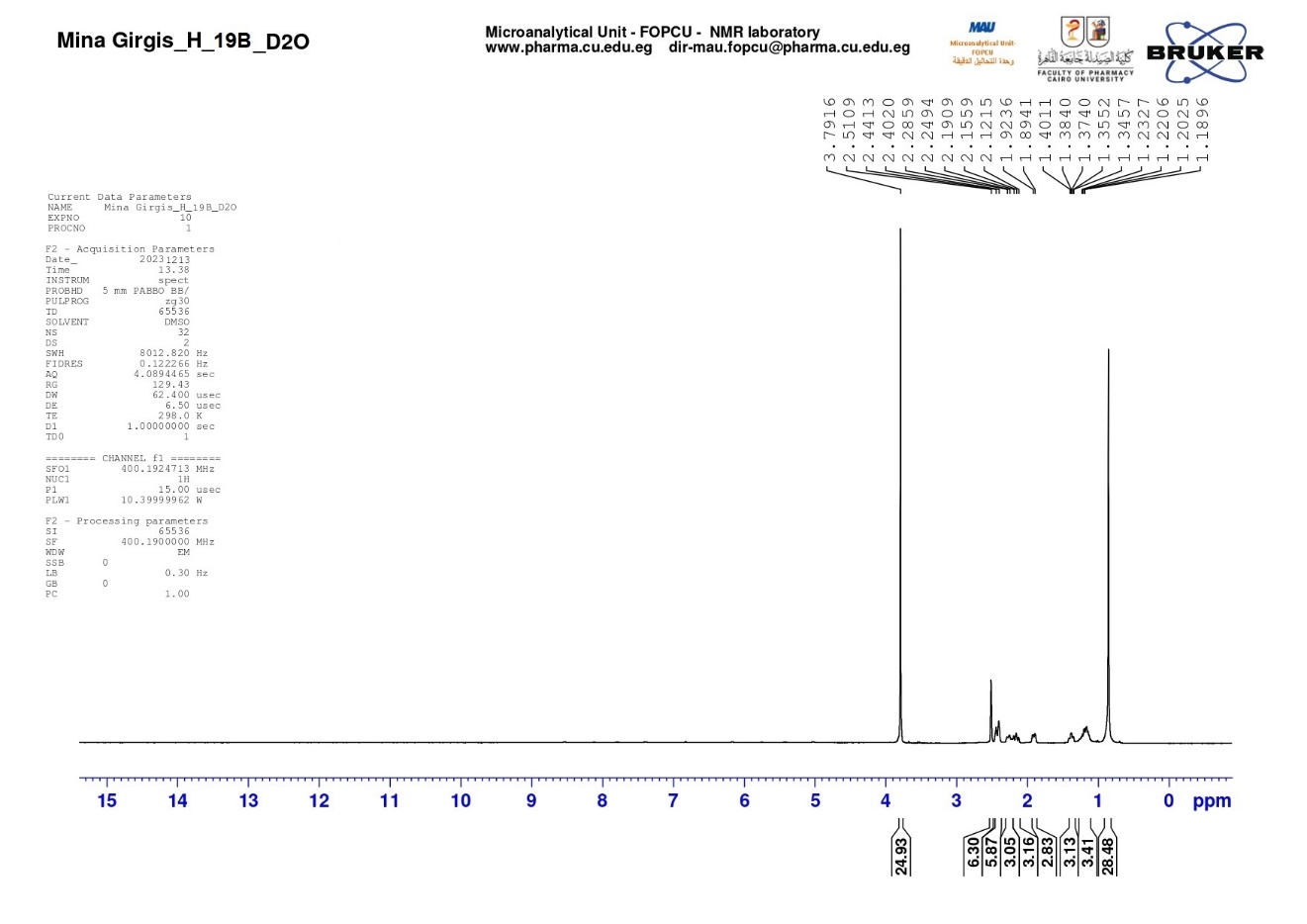


**Figure S65**: ^1^H-NMR (DMSO-*d*6+D_2_O) spectrum of compound **19**

**
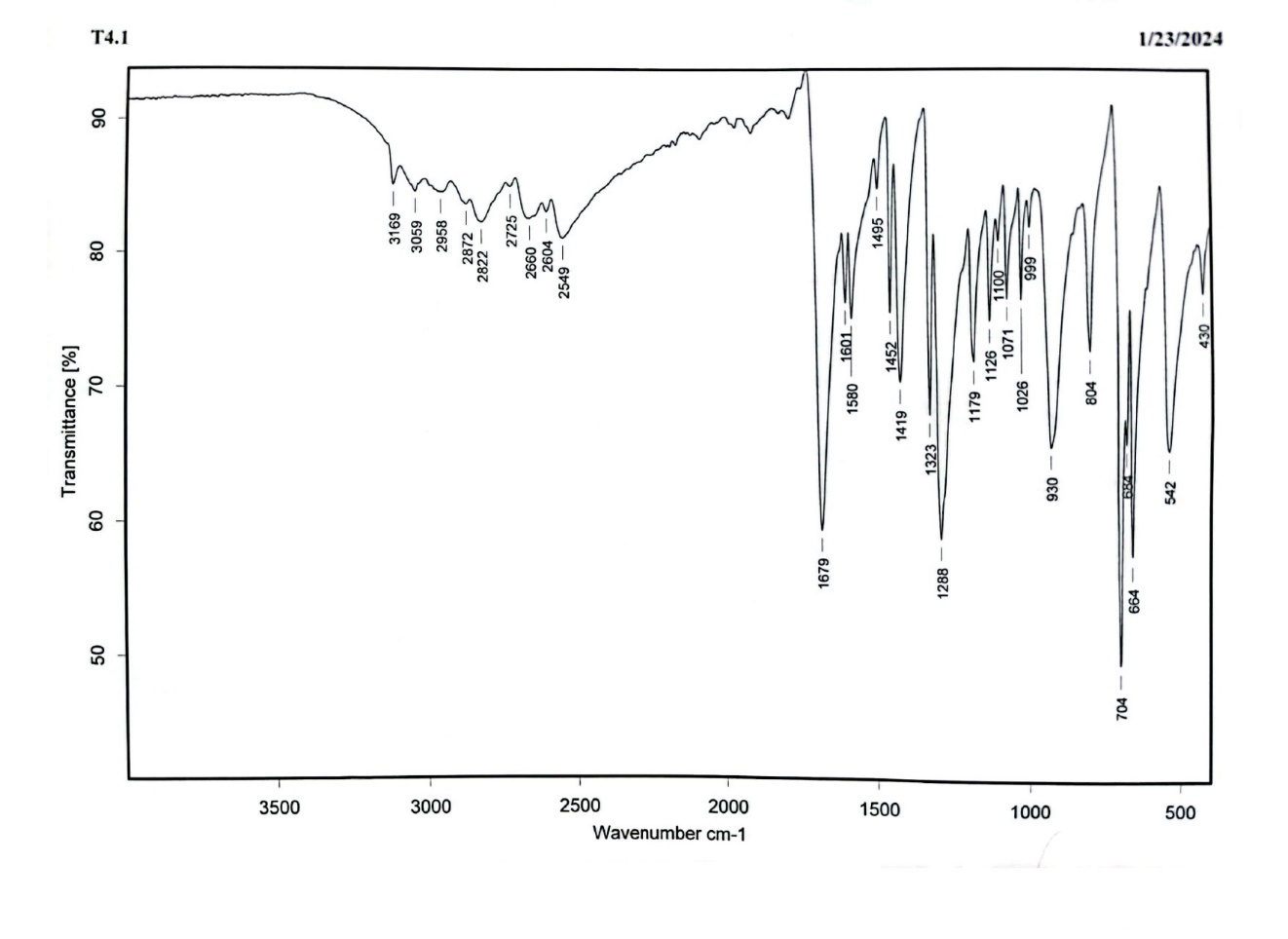
**

**Figure S66**: IR spectrum of compound **20**

**
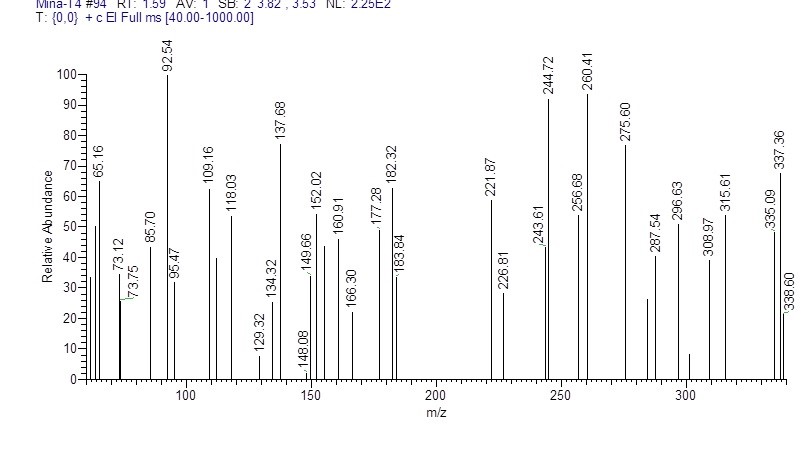
**

**Figure S67**: Mass spectrum of compound **20**

**
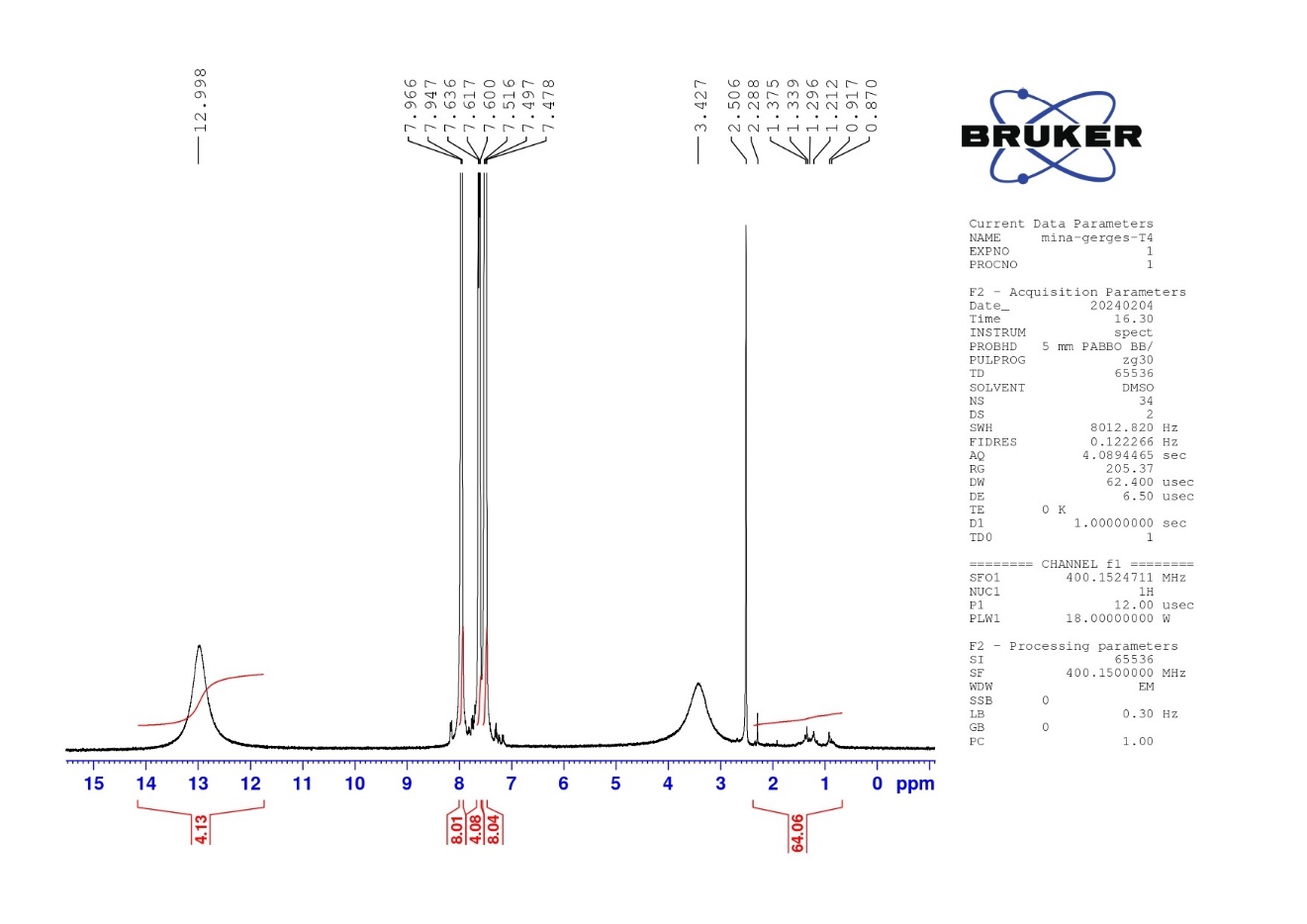
**

**Figure S68**: ^1^H-NMR (DMSO-*d*6) spectrum of compound **20**

**
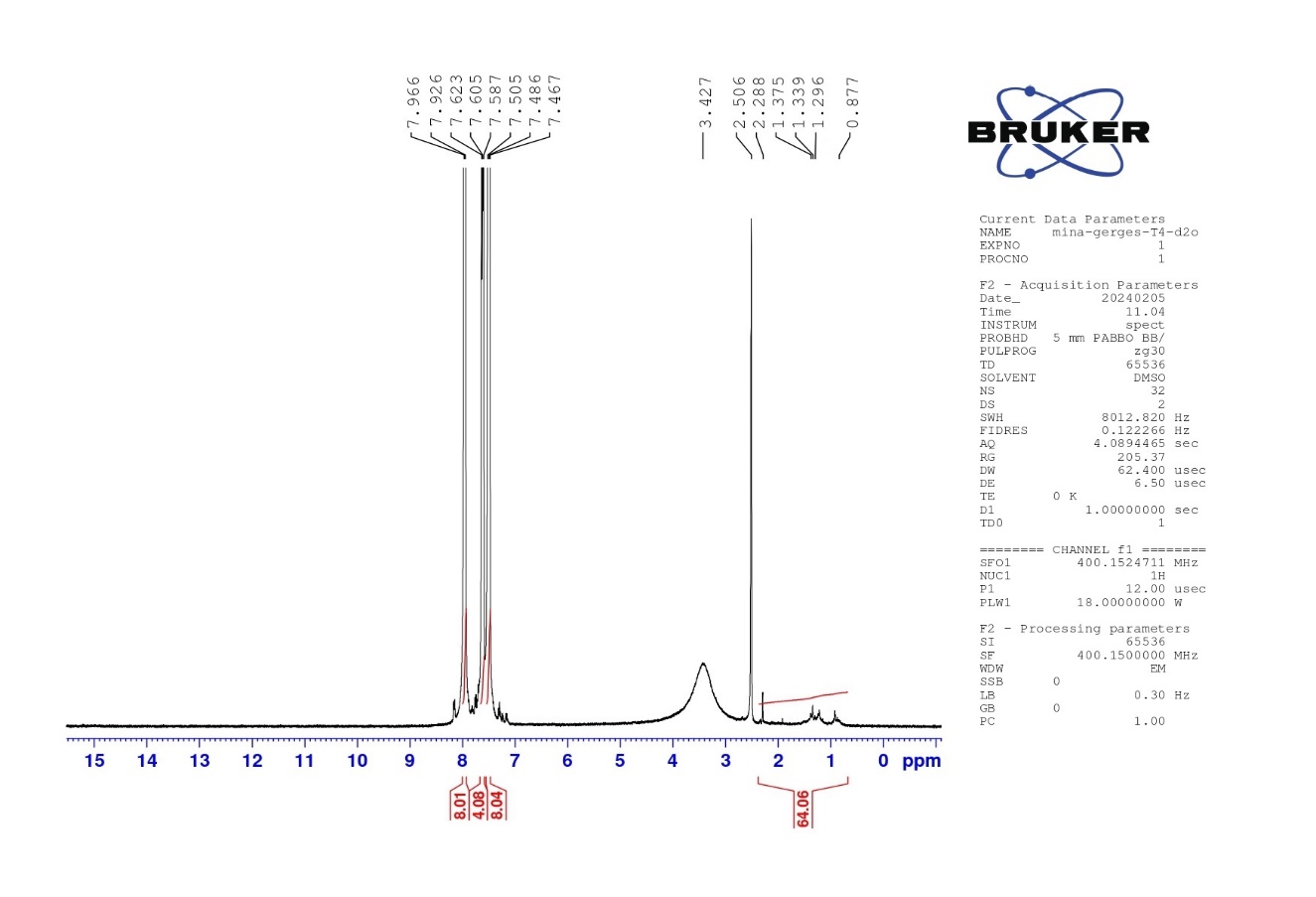
**

**Figure S69**: ^1^H-NMR (DMSO-*d*6+D_2_O) spectrum of compound **20**

**
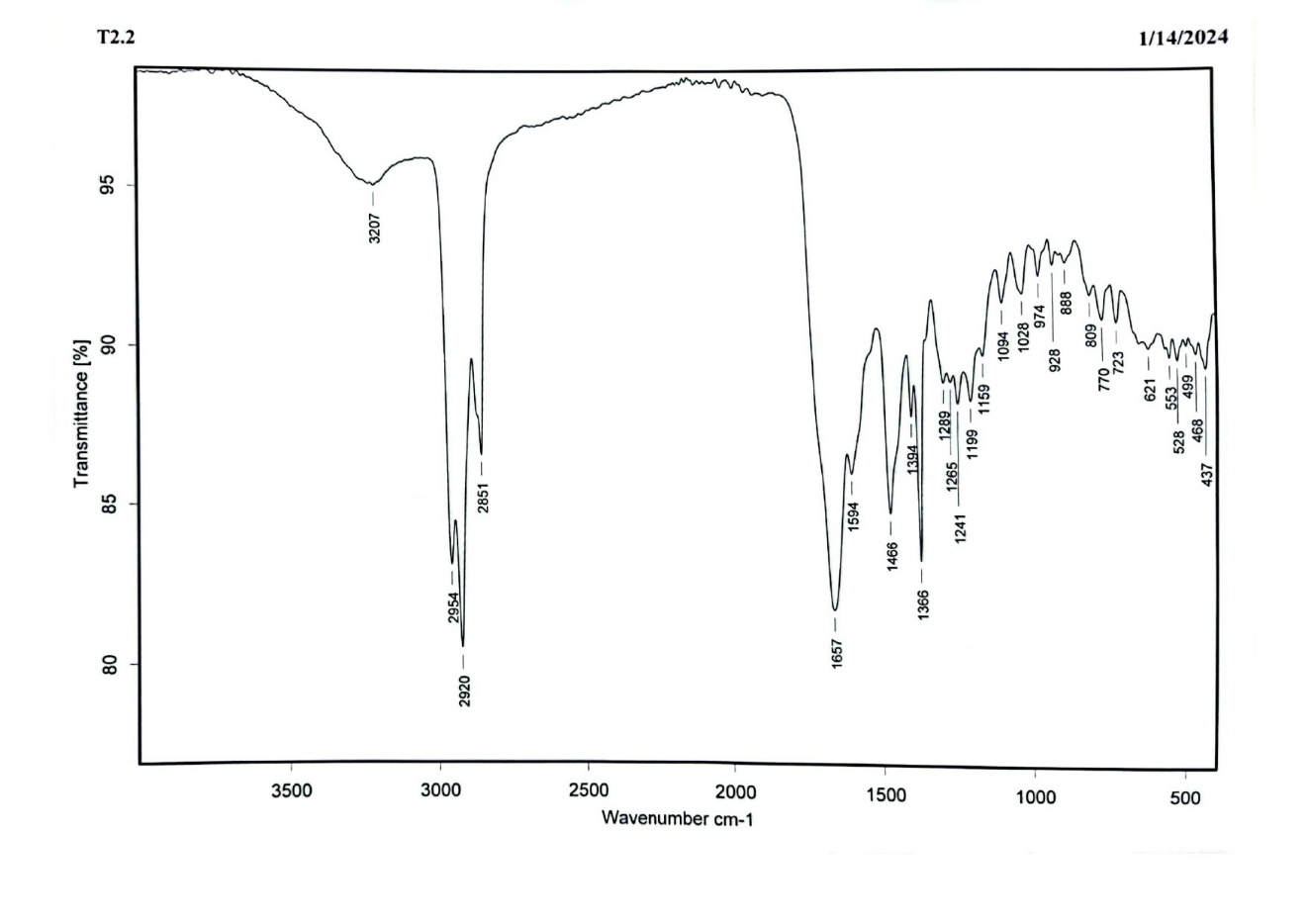
**

**Figure S70**: IR spectrum of compound **21**

**
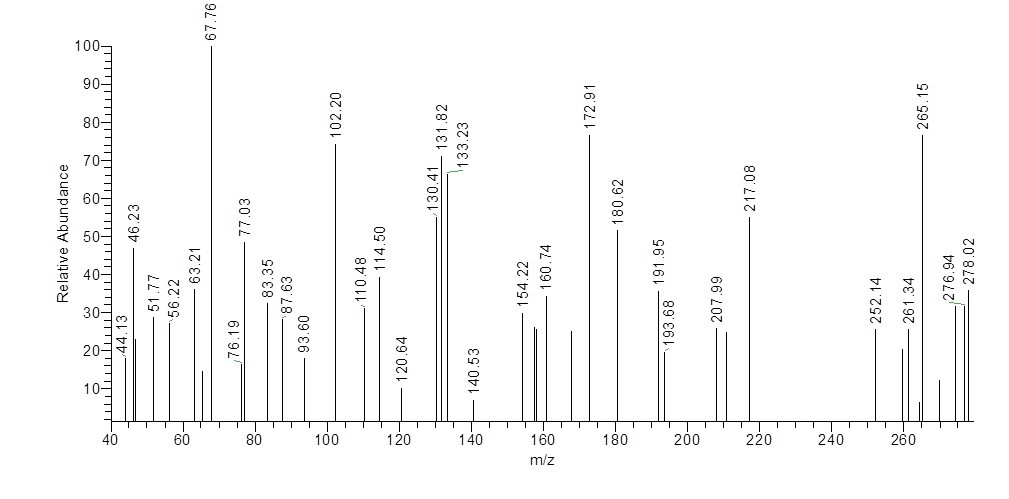
**

**Figure S71**: Mass spectrum of compound **21**

**
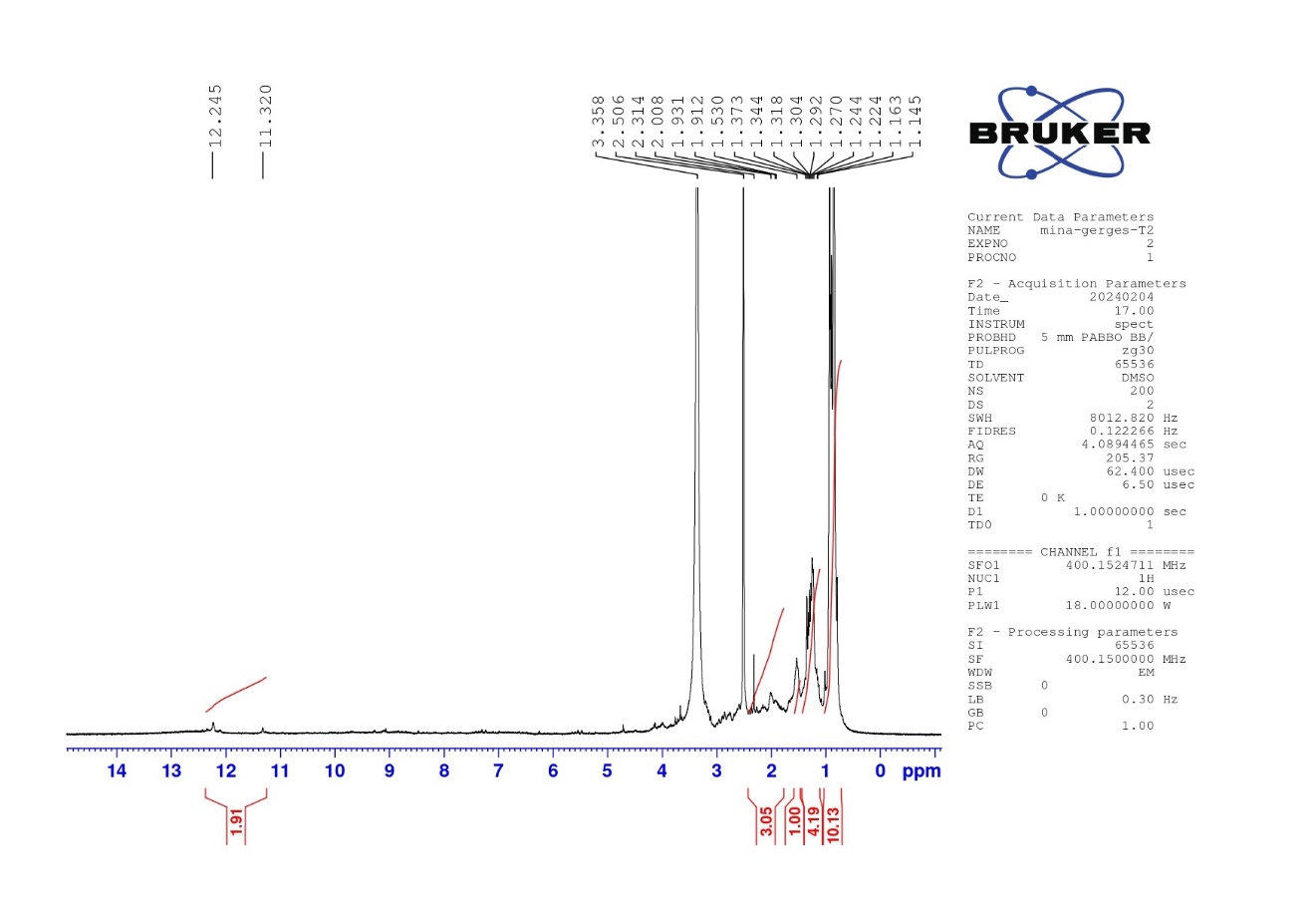
**

**Figure S72**: ^1^H-NMR (DMSO-*d*6) spectrum of compound **21**

**
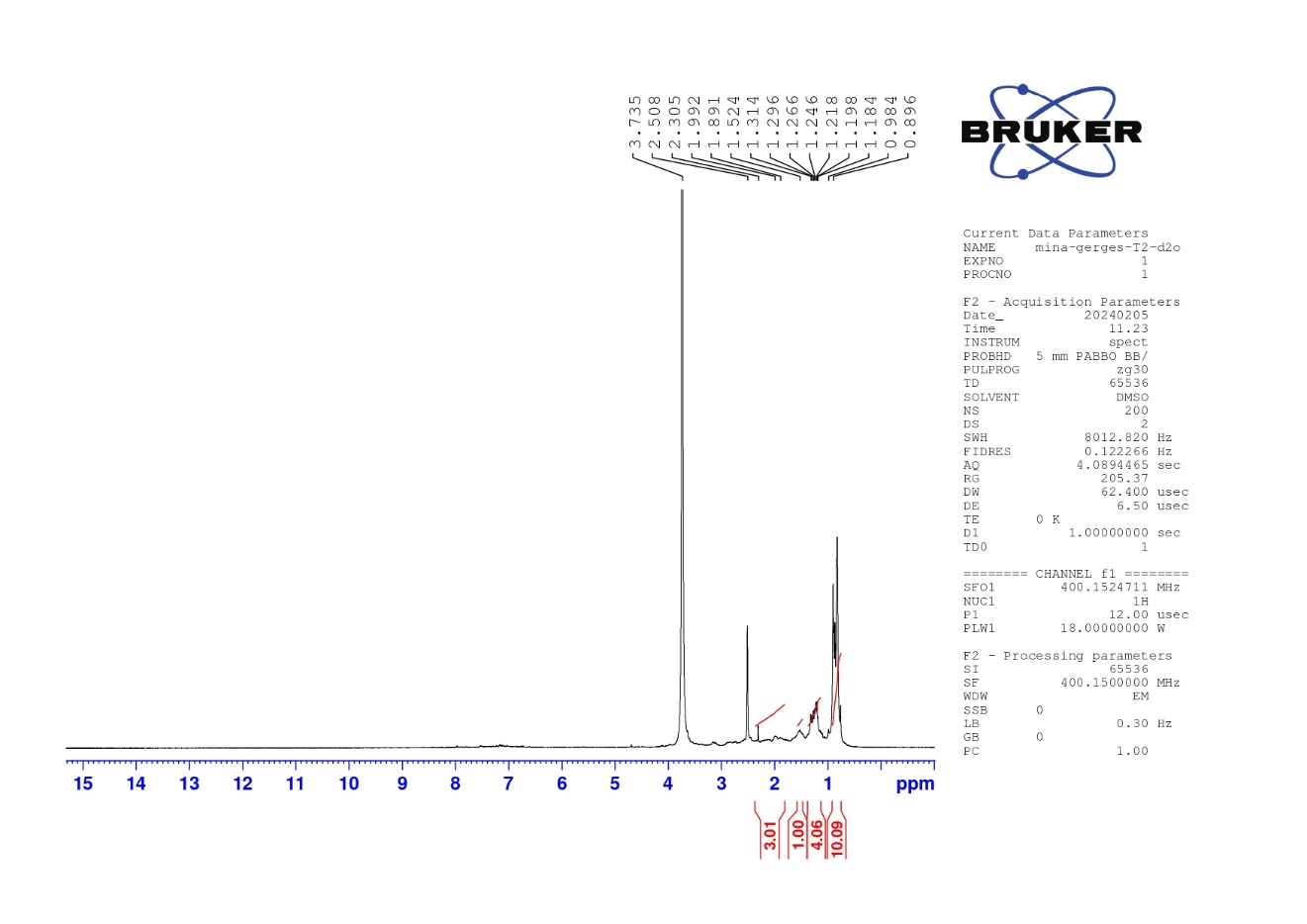
**

**Figure S73**: ^1^H-NMR (DMSO-*d*6+D_2_O) spectrum of compound **21**
